# Quantitative iTRAQ Proteomics Revealed Possible Roles for Antioxidant Proteins in Sorghum Aluminum Tolerance

**DOI:** 10.3389/fpls.2016.02043

**Published:** 2017-01-09

**Authors:** Dangwei Zhou, Yong Yang, Jinbiao Zhang, Fei Jiang, Eric Craft, Theodore W. Thannhauser, Leon V. Kochian, Jiping Liu

**Affiliations:** ^1^Robert W. Holley Center for Agriculture and Health, United States Department of Agriculture - Agricultural Research Service, Cornell UniversityIthaca, NY, USA; ^2^Center of Plateau Ecology, Northwest Institute of Plateau Biology, Chinese Academy of SciencesXining, China; ^3^College of Life Sciences, Fujian Agriculture and Forestry UniversityFuzhou, China; ^4^Agricultural Biotechnology Center, Chengdu Institute of Biology, Chinese Academy of SciencesChengdu, China

**Keywords:** aluminum tolerance, antioxidant system, lignin biosynthesis, proteomics, sorghum

## Abstract

Aluminum (Al) toxicity inhibits root growth and limits crop yields on acid soils worldwide. However, quantitative information is scarce on protein expression profiles under Al stress in crops. In this study, we report on the identification of potential Al responsive proteins from root tips of Al sensitive BR007 and Al tolerant SC566 sorghum lines using a strategy employing iTRAQ and 2D-liquid chromatography (LC) coupled to MS/MS (2D-LC-MS/MS). A total of 771 and 329 unique proteins with abundance changes of >1.5 or <0.67-fold were identified in BR007 and SC566, respectively. Protein interaction and pathway analyses indicated that proteins involved in the antioxidant system were more abundant in the tolerant line than in the sensitive one after Al treatment, while opposite trends were observed for proteins involved in lignin biosynthesis. Higher levels of ROS accumulation in root tips of the sensitive line due to decreased activity of antioxidant enzymes could lead to higher lignin production and hyper-accumulation of toxic Al in cell walls. These results indicated that activities of peroxidases and the balance between production and consumption of ROS could be important for Al tolerance and lignin biosynthesis in sorghum.

## Introduction

Aluminum (Al) is the most abundant metal in the earth's crust, constituting ~7% of the soil (Von Uexküll and Mutert, 1995). At neutral pH, Al primarily exists as relatively insoluble aluminum oxides (Kochian et al., 2015). However, at low pH (pH < 5), rhizotoxic Al^3+^ ions are solubilized into soil solutions from aluminosilicate clays, which inhibits crop root growth and function, leading to significant crop yield losses on acid soils (Von Uexküll and Mutert, 1995). As ~30% of the world's total land area and over 50% of the world's potentially arable lands are acidic, Al toxicity is a major limitation to crop production on acid soils worldwide (Von Uexküll and Mutert, 1995; Kochian et al., 2015).

Plants have evolved several adaptation mechanisms to cope with Al stress. The well-documented Al exclusion mechanism involves a release of organic acids (OAs) from roots into rhizosphere to prevent toxic Al ions from entering into root cells. True Al tolerance mechanisms involve the entry of Al ions into root cells which are then sequestered into the root-cell vacuole and/or translocated to the shoot and stored in leaf cell vacuoles (Kochian et al., 2015).

Over the past decade, the molecular mechanisms underlying Al resistance in plants have begun to be elucidated. Not only have several key Al resistance genes been cloned, but also their functions have been characterized. It has been well-documented that Al exclusion via Al-activated root exudation of OAs, mainly malate and citrate, is widely used by many plant species for dealing with Al stress, including wheat (*Triticum aestivum*) (Delhaize et al., 1993), sorghum (*Sorghum bicolor*) (Magalhaes et al., 2007), barley (*Hordeum vulgare*) (Furukawa et al., 2007), Arabidopsis (*Arabidopsis thaliana*) (Hoekenga et al., 2006; Liu et al., 2009, 2012), and maize (*Zea mays*) (Pellet et al., 1995). Thus, it is not surprising that the first Al resistance genes cloned encode two families of OA efflux transporters: the ALMT (Al-activated malate transporter) family of malate transporters in wheat and Arabidopsis (Sasaki et al., 2004; Hoekenga et al., 2006; Liu et al., 2009), and the MATE (multidrug and toxic compound extrusion) family of citrate efflux transporters in sorghum, barley, and Arabidopsis (Furukawa et al., 2007; Magalhaes et al., 2007; Liu et al., 2009).

As much as 90% of the root Al is localized to negatively charged carboxyl residues in the cell wall and Al disruption of cell wall structure and function could be one of the major causative factors for Al toxicity in root-tip region (Horst et al., 2010; Sivaguru et al., 2013). Recently, increasing lines of evidence indicate that Al tolerance mechanisms underlying modifications of root cell wall carbohydrates (pectins and hemicellulose) to limit the binding of toxic Al ions to the cell wall could play an important role in plants' Al tolerance (Zhu et al., 2012, 2014). The constituents of cell wall polysaccharides, especially pectins and hemicelluloses which are the major Al-binding substrates in cell walls, affect Al binding capacity, and thus Al sensitivity of cultivars upon Al stress (Yang J. L. et al., 2011). In addition, some cell wall modification enzymes are involved in Al tolerance in plants (Zhu et al., 2014).

Aluminum toxicity also causes mitochondrial dysfunction and thus elicits the generation of reactive oxygen species (ROS) and the associated oxidative stress in roots (Horst et al., 1992; Yamamoto et al., 2002; Kochian et al., 2004; Sharma et al., 2012). The Al induced oxidative stress has been considered as a consequence but not a cause of Al toxicity (Navascués et al., 2012). It has been suggested that antioxidant functions can contribute to overall Al resistance (Yamamoto et al., 2002; Sharma et al., 2012).

Sorghum is a staple food for many developing countries, especially those located in semi-arid and tropic areas of the world, where acid soils are prevailed (Dicko et al., 2006). The induction and development of Al resistance in sorghum is a slow process, which is positively and closely associated with the induction of SbMATE gene and protein expression as well as Al-activated and SbMATE-mediated citrate exudation in the root-tip region (Magalhaes et al., 2007; Sivaguru et al., 2013). In Al tolerant sorghum lines, the effects of Al resistance are typically manifested after 3 days of Al treatment (Magalhaes et al., 2007; Sivaguru et al., 2013).

Although the physiological, cellular responses, and gene expression profiles in response to Al toxicity have been studied in sorghum, the cellular, and genetic components that constitute Al-tolerance pathways and networks are still unclear. Quantitative studies of dynamic changes in protein expression profiles during the processes of induction and development of Al resistance in sorghum might provide us new insights into the cellular components that constitute the metabolic pathways, and genetic networks that underlie Al resistance in sorghum.

In recent years, iTRAQ labeling coupled with 2D-LC-MS/MS has provided a platform for simultaneous identification and quantification of proteins through measuring peak intensity of unique, isotope coded reporter ions associated with each sample in the analysis (Ross et al., 2004). As this method can analyze and quantify up to eight phenotypes with high resolution (Ross et al., 2004; Pierce et al., 2008), it has been widely used in model plants such as Arabidopsis (Lan et al., 2011) and rice (Wang et al., 2014), but also has provided a platform to profile and understand the non-model species through comparative proteomics (Yang Y. et al., 2011; Xiong et al., 2015). As a whole system approach, this proteomics technique provides a complementary strategy to unravel the mechanisms underlying plant Al tolerance (Jedmowski et al., 2014; Zheng et al., 2014).

Several proteomics studies on Al stressed roots have been conducted in different plant species such as rice (Yang et al., 2007, 2013; Wang et al., 2014), tomato (Zhou et al., 2009), soybean (Duressa et al., 2011). However, the limitation of these studies is that they did not apply pairwise comparisons between the tolerant and sensitive lines to identify components specifically and differentially induced in the tolerant lines or more advanced proteomics technology was unavailable at the time of the studies. In addition, no biochemical experiments were conducted to verify the involvement and function of the identified putative proteins in Al resistance in these studies.

Here, we used the iTRAQ-labeled quantification technology to integrate metabolic pathway analysis, and biochemical and physiological studies to investigate the dynamic changes of putative Al-responsive proteins from an Al tolerant (SC566) and an Al sensitive (BR007) sorghum line. Our results demonstrate that the tolerant line experienced more dynamic changes in the expression of proteins involved in cellular metabolism and function, while the sensitive line had lower abundance of antioxidant proteins after Al treatment, which is concomitant with higher levels of ROS accumulation and higher levels of lignin deposition and Al accumulation in the root rips of the Al sensitive line. These results suggest that the ROS system, especially the type III peroxidases (PODs), could play an important role in sorghum aluminum tolerance through regulation of lignin biosynthesis and cell wall remodeling which can affect the accumulation of toxic Al in cell walls.

## Results

### Aluminum resistance of two sorghum lines

**Figure 1:**
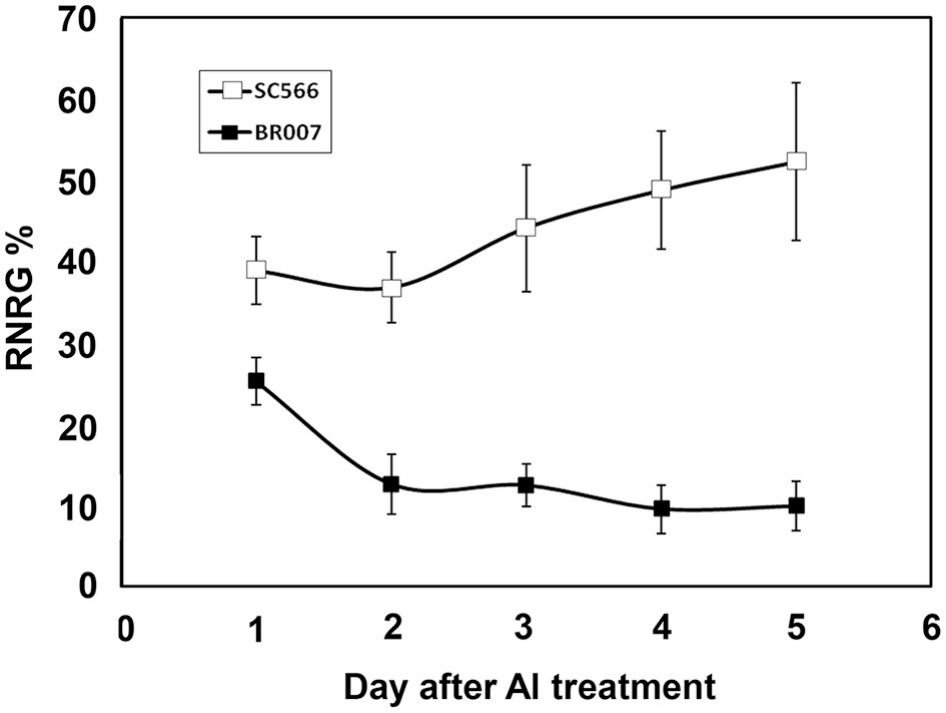
**Relative Net Root Growth (%RNRG) of SC566 and BR007**. Daily %RNRG was calculated as net root growth of +Al treatment / net root growth of −Al treatment at the indicated day of Al exposure. *n* = 20 seedlings.

To evaluate Al resistance, 3-day-old seedlings of Al tolerant SC566 and Al sensitive BR007 sorghum lines were transferred to hydroponic Magnavaca solutions supplemented without or with 27 μM {Al^3+^} at pH 4.0 (Magalhaes et al., 2007). Net root growth (NRG) of each seedling was measured at 1, 2, 3, 4, and 5 days of Al treatment and relative net root growth (%RNRG), i.e., percentage of NRG of +Al treatment/NRG of –Al treatment, was calculated for each day. The results showed that the NRG of SC566 was inhibited by 60% after 1 day of Al treatment. Al resistance was induced over the 5 days of Al treatment with %RNRG increasing to ~50% by the 5th day (Figure 1). In contrast, compared with SC566, root growth of BR007 was more severely inhibited by Al at 1D and %RNRG of BR007 continued to decline to 10% by day 5 (Figure 1). These results were consistent with previous observations that Al resistance in sorghum is an Al-inducible process (Magalhaes et al., 2007).

### Quantitative protein expression profiles in the sorghum root tip regions

**Figure 2:**
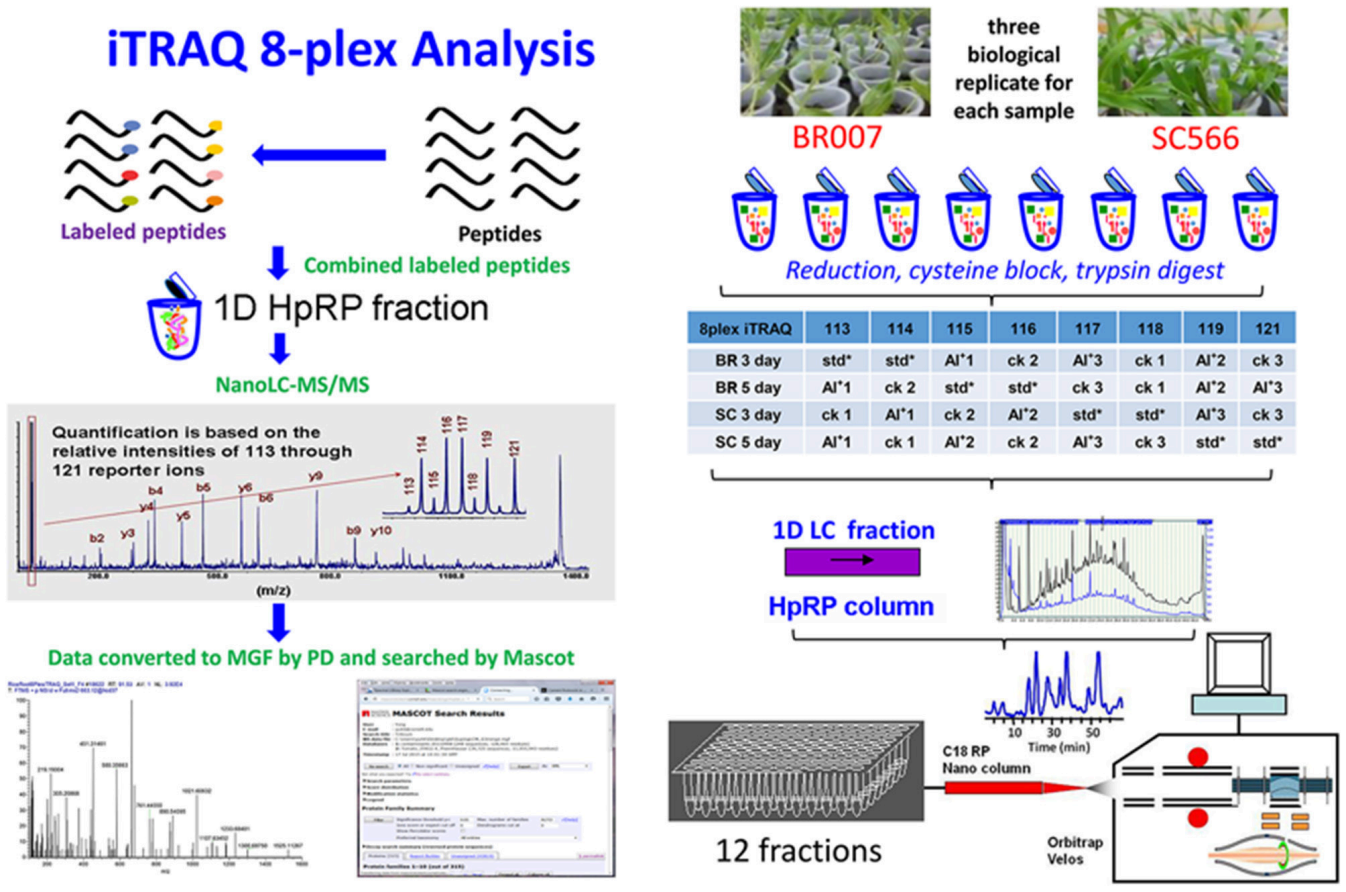
**Experimental design and schematic diagram of the workflow**. HpRP, High-pH reversed-phase chromatography; std, Internal standard which was pooled by equal amounts of all samples in the experiment; MGF by PD, use Proteome Discover software to convert the spectra to MGF format peak list files.

An iTRAQ-based quantitation strategy was utilized to obtain a global view of the proteome dynamics between the Al tolerant (SC566) and Al sensitive (BR007) sorghum lines and to determine the changes of proteins associated with Al response and tolerance (Figure 2). Internal standards constructed by combining equal amounts of proteins from each sample were included in each of the 8-plex iTRAQ set, which could greatly reduce system errors among iTRAQ sets analyzed (Albans et al., 2003; Lilley and Friedman, 2004). A total of 5126–5299 distinct proteins were identified with 95% confidence from 3 to 5 day Al treated samples of BR007 and SC566 (Supplemental Data 1), among which 4375–5082 proteins were found to be present at sufficient amounts to be reliably quantified (Table 1).

**Table 1:** **Summary of protein profiles from root tips of BR007 and SC566**.

	**Days**	**# of total proteins ID**	**# of reliably quantified proteins**	**# of DEPs with *P* ≤ 0.05**	**# of DEPs with >1.5-fold ↑*P* ≤ 0.05**	**# of DEPs with >1.5-fold ↓*P* ≤ 0.05**	**# of DEPs with > ±1.5-fold *P* ≤ 0.05**	**Overlap in 3D+5D**
BR007	3D	5177	5063	1902	345	110	455	237
	5D	5187	5082	1828	333	220	553	
SC566	3D	5126	5050	1430	122	41	163	34
	5D	5299	4375	1393	113	87	200	

*D, day; #, number; ID, identified; DEP, differentially expressed protein; ↑, increase; ↓, decrease*.

**Figure 3:**
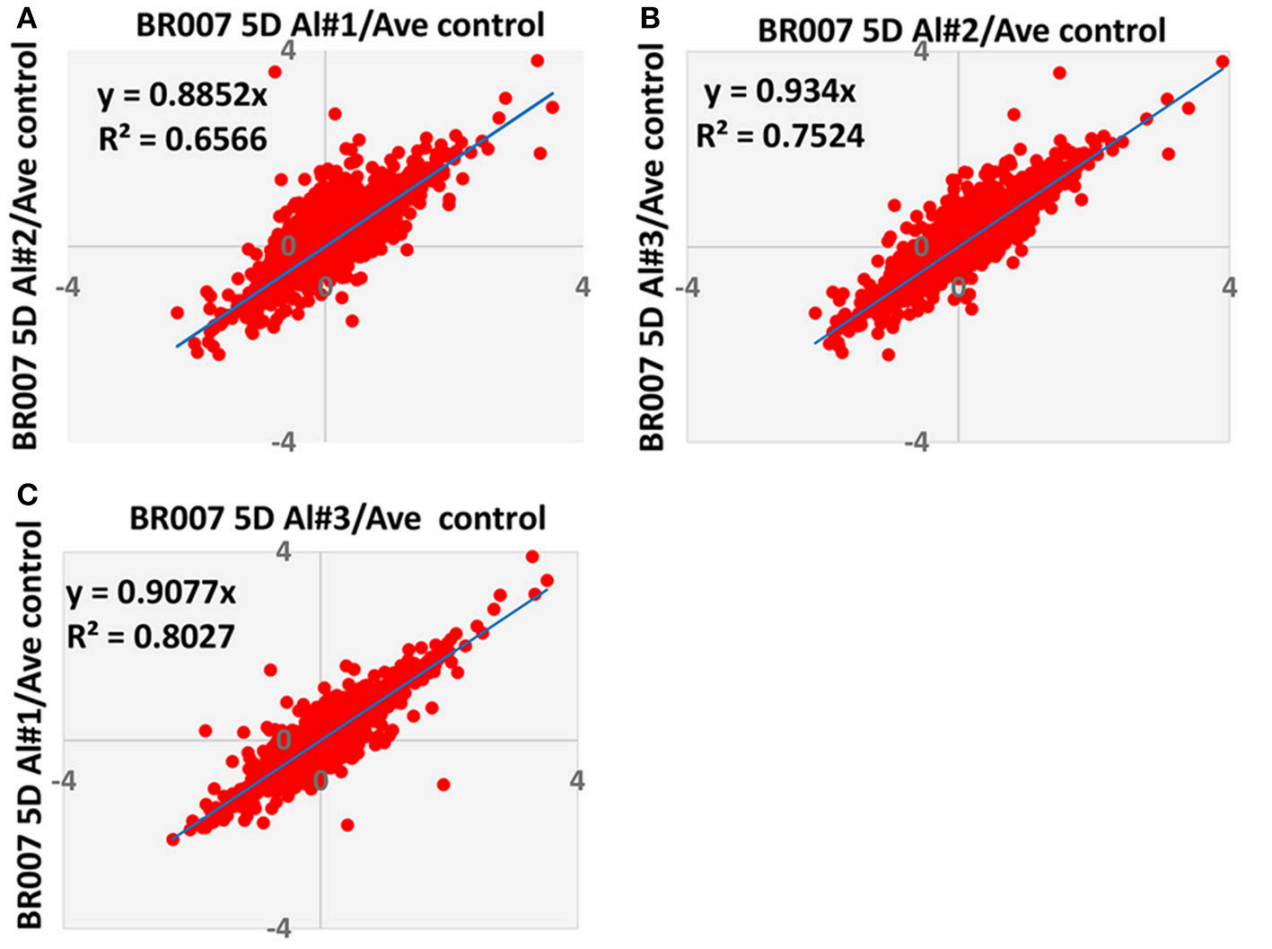
**Comparison of Log2 iTRAQ ratios for all proteins identified in three biological and analytical replicates of each experiment**. This figure shows an example of comparison of ratios from BR007 5D Al treated sample #1, #2, and #3 vs. average control. **(A)** #1 vs. #2; **(B)** #2 vs. #3; **(C)** #3 vs. #1.

To evaluate the quantitative precision and reproducibility of these analyses, a linear regression analysis was carried out. As an example, Figure 3 depicts the plots of the replicates of the BR007 5D treated sample (#1–3) vs. control #1 for all of the 5082 reliably quantified proteins in all replicates and the controls. The slope and *R*^2^-values associated with these plots were found to be 0.8852 and 0.6566 (Figure 3A), 0.9340 and 0.7524 (Figure 3B), and 0.9077 and 0.8027 (Figure 3C), respectively, yielding average slope and *R*^2^-values of 0.9090 and 0.7372. Similar analyses were carried out for the BR007 3D and SC566 3D and 5D experiments. The average slope and *R*^2^-values for these experiments were 0.844 and 0.7270, 0.7263 and 0.5877, and 0.8884 and 0.7527, respectively. The results indicated that the quantitative iTRAQ data from three replicates were reasonably reproducible and linearly correlated. These plots were used to estimate thresholds beyond which fold changes in protein abundance were considered to be significant by the method of “internal error” (Gan et al., 2007). The results suggest that the average internal errors (in log_2_ space) for BR007 3D and 5D, SC566 3D, and 5D were 0.458, 0.502, 0.444, and 0.331, respectively, which corresponded to a fold change threshold of 1.37, 1.42, 1.36, and 1.26, respectively, and represented the “least significant detectable difference,” combining estimates of both technical and biological deviation.

To test these estimates, the expression data from all four experiments were fit to 60 common statistical distributions using EasyFit software (MathWave Technologies, http://www.mathwave.com). The quality of each of the individual fits was evaluated by the *X*^2^ test, the Anderson Darling test and the Kalmogarov-Smirnov (KS) test and in each case the best fit to the experimental data was judged to be the Johnson S_u_ distribution (Castagliola, 1998). The threshold of significance corresponding to the 95% confidence interval was estimated from identically distributed theoretical data sets generated by the Easyfit software. These were found to closely approximate the (±) 2σ convention for normally distributed data. Using these analyses, the thresholds of significance (in log_2_ space) corresponding to the 95% confidence interval for experiments1–4 were estimated to be ±0.77, ±0.88, ±0.54, and ±0.58, respectively, corresponding to fold changes of 1.7, 1.8, 1.5, and 1.5, which are clearly more conservative than those estimated through the method of “internal error.”

Combining the results of both approaches, we arrived at a compromise estimate of ±1.5-fold as the threshold of significance. While this undoubtedly increases the probability of false positive results slightly, it correspondingly decreases the probability of false negative results which translate to “missed discoveries.” At this stage of our analysis, missed discoveries are considered to be the greater evil. The experimental expression data for each quantified protein was subject to a *t*-test. Only those proteins that exhibited a fold change greater than the threshold of significance and a *p*-value of <0.05 are considered to be significantly changed with high confidence. Based on these criteria, 113–345 reliably quantified proteins could be classified as differentially up-regulated proteins with >1.5-fold changes (*p* < 0.05) between the treatment and the control, while 41–220 proteins could be classified as differentially down-regulated proteins with >1.5-fold changes (*p* < 0.05; Table 1; Supplemental Data 1). These differentially expressed proteins (DEPs) were selected for further biological and functional analysis (Tables S1–S4).

### Functional classification of differentially expressed proteins

**Figure 4:**
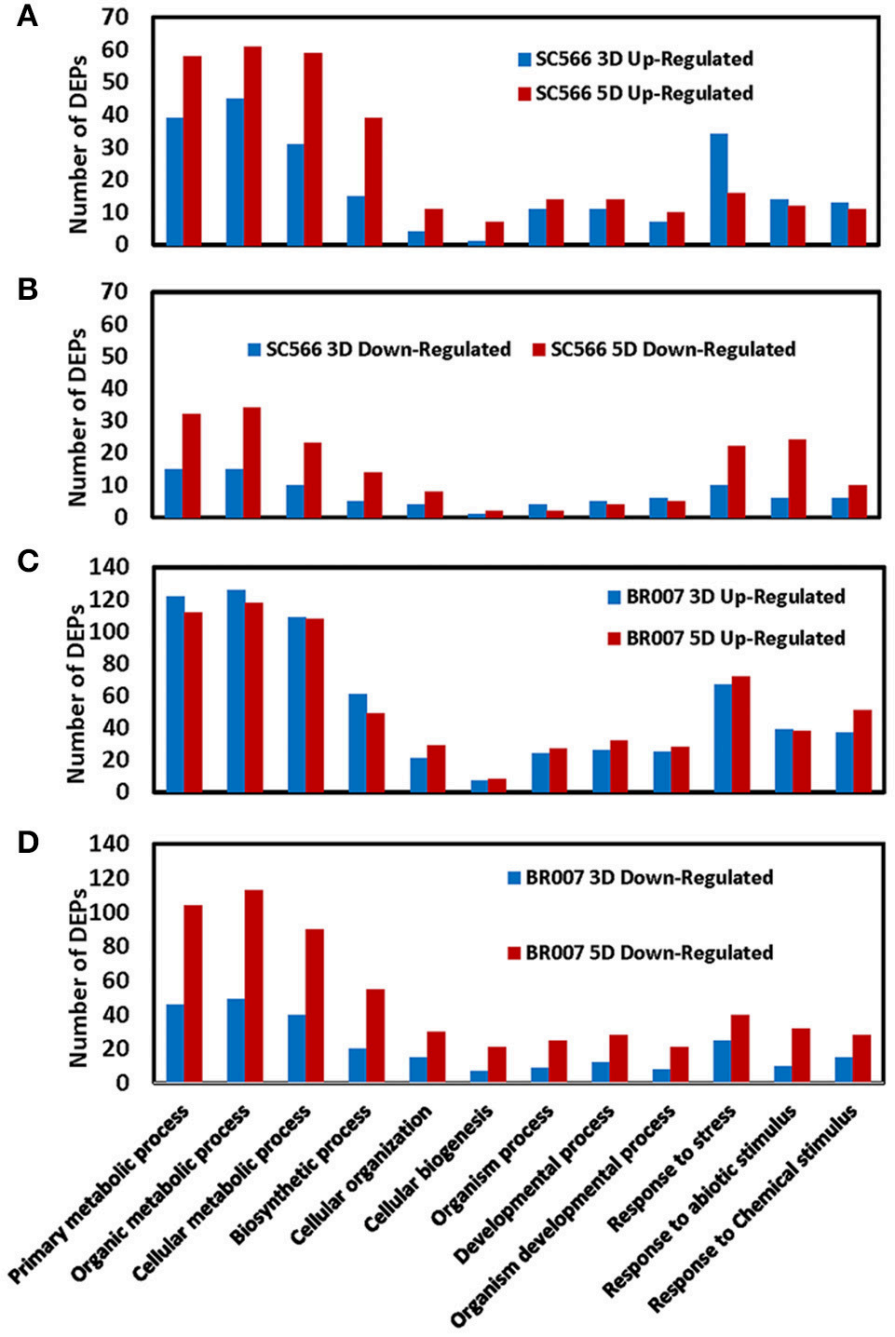
**Number of up- and down-regulated DEPs of SC566 and BR007 in functional categorization**. Functional categorization of 1.5-fold differentially expressed proteins was analyzed for both SC566 **(A,B)** and BR007 **(C,D)** treated with Al at indicated time points using the Blast2GO 2.8 software with default NCBI nr/nt Blast databases and settings.

We used the agriGO software (Du et al., 2010) to categorize the DEPs of BR007 and SC566 with respect to their functions. We observed large increases in the numbers of the up- and the down-regulated DEPs involved in basic cellular, metabolic and biosynthetic processes as well as in cellular organization from 3D to 5D of Al treatment in SC566 (Figures 4A,B). In contrast, less up-regulated (Figure 4A) and more down-regulated DEPs (Figure 4B) involved in responses to stresses were found at 5D than at 3D of Al treatment in SC566. For proteins involved in cellular biogenesis and developmental processes, more DEPs were up-regulated (Figure 4A) and less DEPs were down-regulated (Figure 4B) from 3D to 5D in SC566. The increased expression of proteins involved in basic cellular and developmental functions and the decreased expression of proteins involved in stress responses from 3D to 5D were coincident with the recovery of root growth inhibition by Al stress in SC566 during the same period (Figure 1; Magalhaes et al., 2007). For the Al-sensitive BR007 line, in general, there were no changes in the number of up-regulated DEPs between 3D and 5D in each of the categories listed (Figure 4C). However, there were significant increases in the numbers of the down-regulated DEPs in all of the listed categories at 5D than at 3D (Figure 4D). These results were consistent with the fact that at 5D of Al treatment, the roots of BR007 encountered further losses of cellular, metabolic, developmental, and defensive functions which was consistent with the stunted root phenotype of BR007 at 5D (Figure 1).

Although much more DEPs were found in BR007 than in SC566 at both 3D and 5D of Al treatment (Table 1), the numbers of up-regulated DEPs involved in cellar and developmental functions increased from 3D to 5D in SC566 but not in BR007 (Figures 4A,C). In addition, compared with SC566, much larger portions of the DEPs overlapped between 3D and 5D in BR007 than in SC566: the overlapped DEPs accounted for 52 and 43% of the total DEPs for 3D and 5D in BR007, respectively, while only 21 and 17% of the total DEPs were overlapped for 3D and 5D in SC566, respectively (Table 1). These results indicated that roots of the Al-tolerant SC566 experienced more dynamic changes in proteomes from 3D to 5D in SC566, which was coincident with the active recovery of SC566, but not BR007, from Al damage in this period (Figure 1; Magalhaes et al., 2007).

### Anti-oxidative and detoxification proteins

One of the key deleterious effects of Al toxicity on root growth is to disturb ROS balance in plants, and it has been suggested that the cellular antioxidant and free radical scavenge systems could play a role in tolerance to Al-induced ROS stresses (Jones et al., 2006).

**Figure 5:**
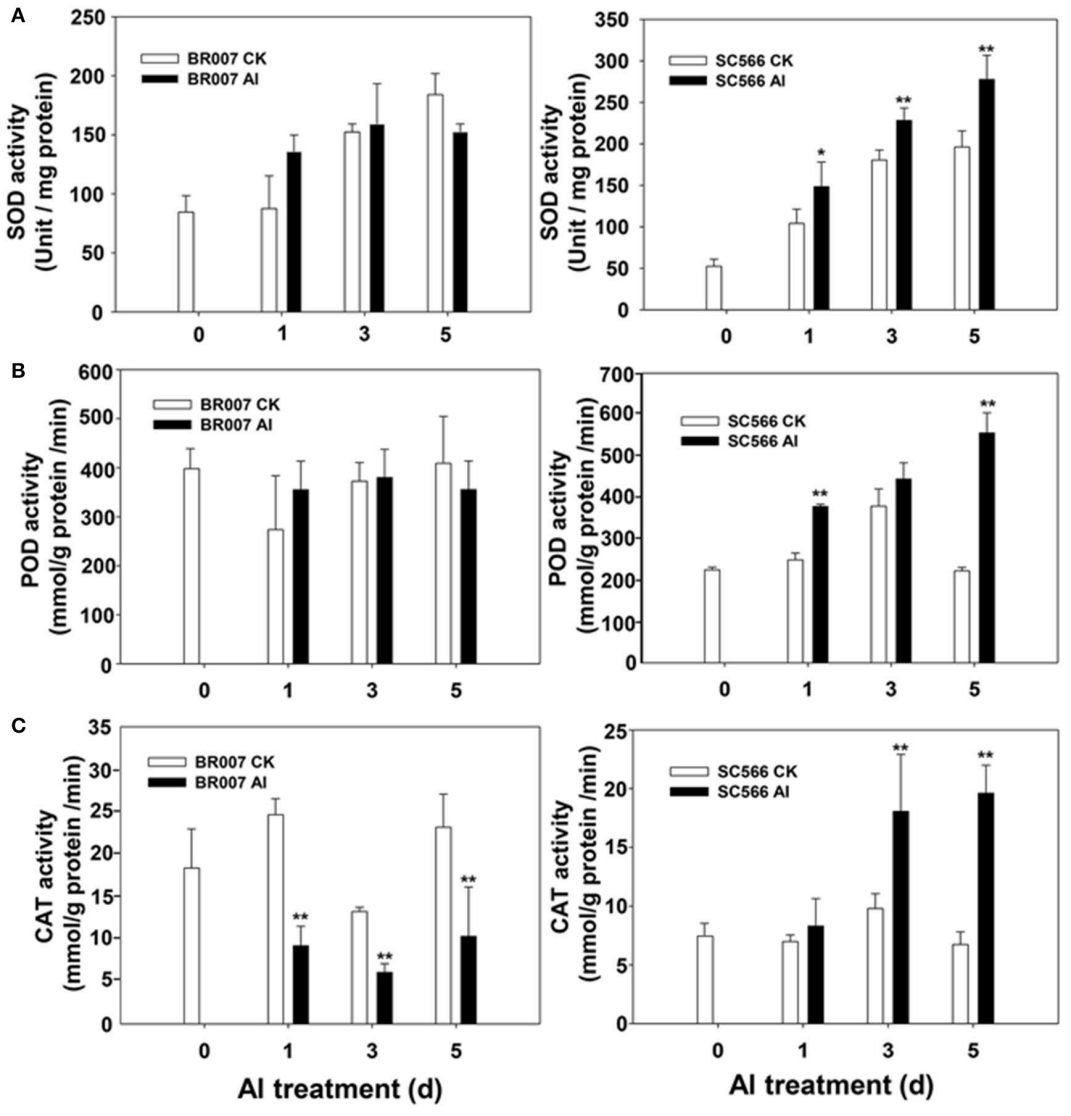
**Enzymatic activities**. Root tip samples (0.5 g FW) were homogenized in the HEPES-KOH buffer + 0.1 mM EDTA (pH 7.8). Supernatant of each sample was collected for enzymatic activity assays: **(A)** Superoxide dismutase (SOD), **(B)** Peroxidase (POD), and **(C)** Catalase (CAT) in root tips of BR007 and SC566 with Al or without Al (CK) treatment. *n* = 3.

In our previous study, we found that under Al stress, an Al sensitive sorghum line accumulated much higher levels of ROS in the root tip region than did its Al-tolerant counterpart (Sivaguru et al., 2013). In the current study, we found that the levels of many antioxidant proteins in SC566 and BR007 were changed significantly after Al stress (Table S5). While a large portion of these proteins were down-regulated in BR007, most of them were up-regulated in SC566 after Al treatment (Table S5). To evaluate antioxidant activities in the Al sensitive BR007 and the Al tolerant SC566, enzymatic activities of three important antioxidant enzymes, i.e., superoxide dismutase (SOD), peroxidase (POD), and catalase (CAT), were measured in the root tips of these two lines with or without Al treatment. Our results indicated that although the SOD activities were increased in root tips of BR007 treated with Al in 1D, no significant differences in SOD activities in the root tips of BR007 between the control and treatment were found at 3D and 5D (Figure 5A). However, compared with the controls (–Al), the SOD activities in root tips of SC566 treated with Al were significantly higher at 3D and 5D (Figure 5A). Similar trends were observed for POD (Figure 5B) and CAT where the activities of POD and CAT were higher in the Al-treated SC566 line than those in the corresponding controls, especially at 5D (Figure 5C), while the enzymatic activities either remained no changes for POD (Figure 5B) or decreased after Al treatment for CAT (Figure 5C) for the Al sensitive BR007 line. These results suggest that lower amounts of ROS accumulation in the root tips of Al tolerant sorghum lines could be explained by their higher levels of activities of antioxidant enzymes and thus higher capacities for ROS management compared with the sensitive ones (Figure 5; Sivaguru et al., 2013).

### Aluminum stress enhances the expression of proteins involved in the phenylopropanoid biosynthesis pathway

The phenylopropanoid pathway is associated with the biosynthesis of several important secondary compounds such as flavonoids, lignin and phenolic acids (Fraser and Chapple, 2011). Some of the phenylpropanoid pathway metabolites and their antioxidant activities in plants have been suggested to be involved in tolerance of plants to metal toxicities (Izbiańska et al., 2014). The results of our proteomic studies indicated that six proteins involved in the phenylpropanoid metabolic process and the production of lignin, including 4-coumarate:CoA ligase (4CL), Cinnamoyl coA:NADP (CCR), caffeic acid/5-hydroxyferulic acid O-methyltransferase (OMT1), phenylalanine ammonia lyase 1 (PAL1), cinnamyl alcohol dehydrogenase 4 (CAD4), and UDP-glucosyl transferase (UGT), were differentially expressed in SC566 and BR007 (Table 2; Figures S1, S2). While the levels of PAL1, CAD4, and OMT1 were up-regulated at 3D in SC566, their levels were comparable to those of the control plants after 5D of Al exposure. However, for BR007, not only were PAL1, OMT1, UGT1 up-regulated by Al treatment in root tips at 3D and 5D, but CCR and UGT were also highly up-regulated at 5D (Table 2). These data suggest that under Al stress, enzymes involved in the biosynthesis of flavonoid or lignin related compounds were more abundant and active in the root tips of BR007 than in SC566.

**Table 2:** **Relative protein expression (fold changes) of key enzymes in the phenylptropanoid pathway in BR007 and SC566 under aluminum treatment using iTRAQ technology**.

**Accession version**	**Gene**	**SC566 3D**	**SC566 5D**	**BR007 3D**	**BR007 5D**
EES12521.1	PAL1 (PHE ammonia lyase 1)	1.64 ± 0.07	N	1.82 ± 0.13	N
EER96736.1	CCR4 (cinnamoyl coA:NADP oxidoreductase)	N	N	N	1.57 ± 0.13
EES04623.1	4CL2 (coumarate:CoA ligase 3)	N	N	N	N
BAF42789.1	CAD4 (cinnamyl alcohol dehydrogenase 4)	1.75 ± 0.07	N	N	N
EES08408.1	OMT1 (caffeic acid/5-hydroxyferulic acid O-methyltransferase)	N	N	2.31 ± 0.51	N
AAO43609.1	OMT1 (caffeic acid/5-hydroxyferulic acid O-methyltransferase)	2.20 ± 0.18	N	N	N
EER89617.1	UGT71C (UDP-glucosyl transferase 71C4)	N	N	N	1.63 ± 0.15
EES14846.1	UGT88A1 (UDP-glucosyl transferase 88A1)	N	N	N	1.71 ± 0.04
EES05311.1	UGT85A2 (UDP-glucosyl transferase 88A2)	N	N	N	1.87 ± 0.14
CAX02213.1	UGT84A1 (UDP-glucosyl transferase 84A1)	N	N	1.58 ± 0.04	1.85 ± 0.10
AAM94296.1	UGT73C7 (UDP-glucosyl transferase 73C7)	N	N	2.66 ± 0.14	2.91 ± 0.13
EER94182.1	UGT74F4 (UDP-glucosyl transferase 74F4)	N	N	1.77 ± 0.06	1.92 ± 0.02
EES19767.1	UGT73B3 (UDP-glucosyl transferase 73B3)	N	N	3.84 ± 0.35	3.70 ± 0.68

*N represents that there is no significant change compared to control. Data are mean ± SE*.

**Figure 6:**
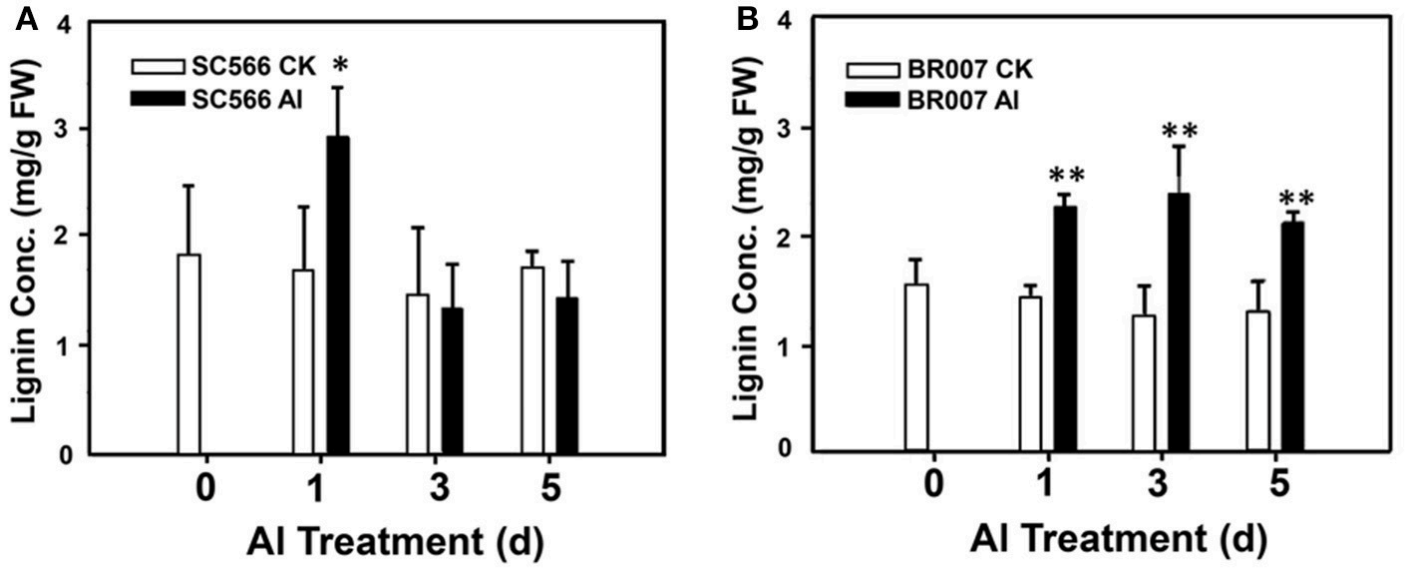
**Root tip lignin production**. Root tip samples (0.5 g FW) were homogenized in 95% EtOH. Lignin contents of the washed and air-dried pellet samples were determined for **(A)** SC566 and **(B)** BR007 under Al or control (CK) treatment. *n* = 3. ^*^p < 0.05; ^**^p < 0.01.

To further address this hypothesis, lignin production in both lines were measured without or with Al treatment. The results indicated that the lignin content in the root tips of SC566 was enhanced by 1D of Al treatment compared with the control and then it was back to the control levels at 3D and 5D, which was coincident with the recovery of root growth of SC566 after 1D of Al treatment (Figure 6A). However, for BR007, lignin production remained significantly higher in the Al-treated samples at 1D, 3D, and 5D of Al treatment compared to those in the −Al treatment (Figure 6B).

### Protein-protein interaction analysis

Protein-protein interactions play an important role in achieving proper cellular function. In order to understand the complex relationships of all direct and indirect protein interactions in sorghum roots, we used the high score corresponding homologous proteins from the Arabidopsis database to obtain protein interaction networks using the STRING software (http://string-db.org, Search Tool for the Retrieval of Interacting Genes/Proteins; Szklarczyk et al., 2015).

**Figure 7:**
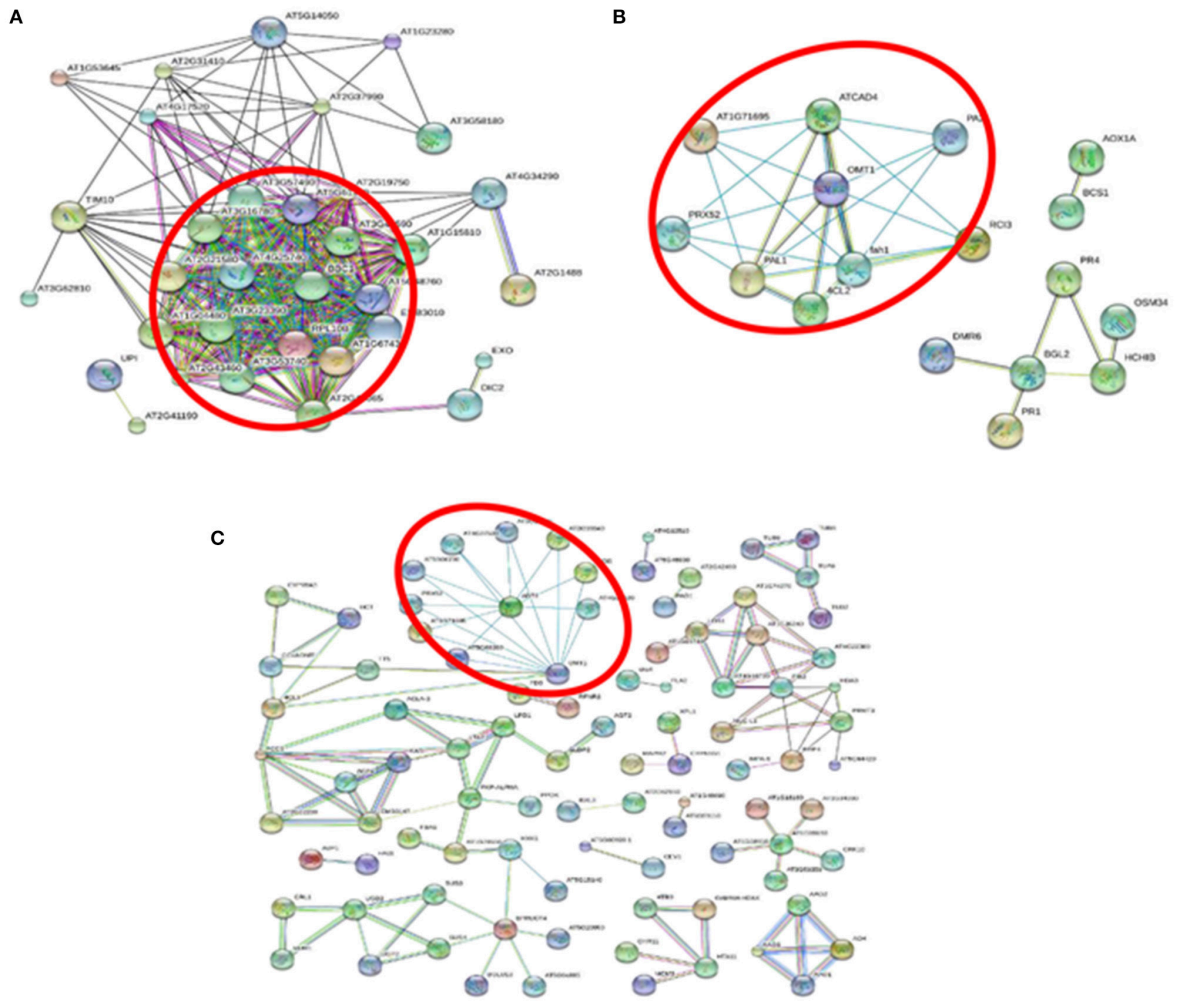
**STRING-based protein-protein interaction analysis with high confidence interaction score of 0.700. (A)** STRING analysis for up-regulated differentially expressed proteins (DEPs) in SC566 at 5D Al treatment. The circled nodes are Ribosomal family proteins indicating that protein biosynthesis is highly active for the Al tolerance line under Al stress. **(B)** STRING analysis for up-regulated DEPs in SC566 at 3D Al treatment. **(C)** STRING analysis for down-regulated DEPs in BR007 at 3D and 5D Al treatment. The circled nodes in **(B,C)** are peroxidase family proteins.

An interaction network of the ribosomal family proteins was detected involving 87 proteins with at least 1.5-fold increases in +Al/−Al ratios in SC566 5D (Figure 7A). Such interactions of differentially expressed ribosomal family proteins were not identified from BR007 5D. These results suggested that protein biosynthesis remained highly active in the root tips of SC566, but not in the root tips of BR007, after 5D exposure to Al. The STRING analysis also indicated that proteins involved in ROS cleavage processes were up-regulated and showed strong interactions with each other at 3D of Al treatment in SC566 (Figure 7B), while the differentially down-regulated POD family proteins after 3D and 5D of Al exposure in root tips of BR007 also showed interactions (Figure 7C).

## Discussion

Sorghum is the fifth most important cereal and an important biofuel crop. Sorghum also possesses an extraordinary ability to cope with abiotic stresses. In recent years, the sorghum transcriptome networks have been analyzed to reveal the molecular basis underlying sorghum tolerance to different abiotic stresses (Dugas et al., 2011; Sui et al., 2015). However, the adaptive mechanisms underlying sorghum Al tolerance at the proteomic level are still unclear. In this study, we used an 8-plex iTRAQ comparative proteomic strategy to analyze the dynamic changes of protein profiles in the root tip regions of the Al tolerant (SC566) and the Al-sensitive (BR007) sorghum lines with or without Al treatment.

In sorghum, Al resistance is slowly induced by Al stress. For instance, at 1D of Al treatment, the Al resistance mechanisms have not been induced and thus, at this time point, both of the Al tolerant and Al sensitive lines displayed similar sensitivity to Al toxicity (Figure 1; Magalhaes et al., 2007; Sivaguru et al., 2013). The sensitive phenotypes include: accumulation of Al in the root tip region, loosening of the root-tip cell wall structure, swollen root-tip cells, inhibition of root growth, damaged plasma membrane and cell integrity of the root tip cells, Al-induced callose (1,3-β-D-glucan) formation, and the bursts of ROS in the root tip region, all of which are typical secondary responses to Al-caused cell damage (Magalhaes et al., 2007; Sivaguru et al., 2013). However, from 3D of Al treatment, with the induction of Al resistance mechanism in the tolerant but not the sensitive lines, the root growth as well as root cell structure and function begin to recover from Al-caused root damage in the tolerant lines (Figure 1; Magalhaes et al., 2007; Sivaguru et al., 2013). By the 5D of Al treatment, root growth and function are fully recovered from Al stress in the tolerant lines, but not in the sensitive lines (Magalhaes et al., 2007; Sivaguru et al., 2013).

Therefore, sampling 3D and 5D could allow us to catch the most dynamic changes underlying the induction and development of Al tolerance in the tolerant line, while avoiding gathering a possible majority of overlapping information related to secondary responses to the Al-toxicity-caused damage shared between the tolerant and the sensitive lines at 1D. In addition, our results indicated that 3D could be not too late to catch the possible earlier tolerance response/mechanism. For instance, in Figure 6A, the tolerance line displayed enhanced Al-induced lignin production in roots only at 1D but not at 3D and 5D. However, the lignin productions in the sensitive line treated with Al never went down (Figure 6B). We know that lignin production at the root is positively associated with ROS levels but negatively associated with antioxidant activities, which results in decreased levels of ROS and thus lowered lignin production. Thus, although we did not include 1D in our sampling, our proteomic analyses did catch the important involvement of antioxidant enzymes in Al tolerance from the 3D samples of the tolerant line (Figure 7). For the reasons mentioned above, we chose 3D and 5D, but excluded 1D, for proteome profiling.

### Proteomic dynamics under Al stress

The results of our proteomic analysis were in line with the active recovery of the Al tolerant SC566 line from the damage caused by Al toxicity (Figure 1). The STRING analysis showed unique interactions between ribosomal family proteins which were differentially up-regulated in SC566 after 5D of Al treatment (Figure 7). This result suggests that the active protein synthesis could play a critical role for SC566 to cope with Al stress.

In sorghum, *SbMATE* is a major Al tolerance gene that encodes a citrate transporter facilitating Al-activated root citrate exudation upon Al stress (Magalhaes et al., 2007). In our previous study, we found that the expression and accumulation of the SbMATE protein was enhanced in the epidermal and cortical cells in the root tip region of the Al tolerant sorghum line but not in the Al sensitive line. In addition, the expression of antioxidant enzymes was enhanced in the root tips of the Al tolerant line, but not in the sensitive line (Figure 5). The association of active protein synthesis and the expression of the proteins, such as SbMATE, involved in the Al tolerance processes suggests that increased activities of protein biosynthesis under Al stress could play a key role in expression of Al tolerance in sorghum. Furthermore, compared with the sensitive BR007, less DEPs were overlapped during the course of recovery of the tolerant SC566 from Al-induced damage. We noticed that proteins involved in metabolic, biosynthetic, and developmental processes were up-regulated in the tolerant SC566 line at 5D of Al treatment (Figure 4A), while proteins in the same categories were more down-regulated at 5D compared with at 3D for the sensitive BR007 line (Figure 4D). These results suggest that dynamic protein changes in response to Al stress are likely to be a key factor for plants to cope with Al toxicity (Table 1).

### ROS scavenging system

ROS includes superoxide anion (O_2_^.−^), hydroxyl radical (.OH), hydrogen peroxide (H_2_O_2_), and singlet oxygen (^1^O_2_; Richards et al., 1998), which are produced under adverse environmental conditions. ROS can oxidize and damage important cellular constituents such as proteins, nucleic acids, and lipids. Therefore, controlling ROS levels in the plant cell is critical for plant survival under abiotic stress. Like other stresses, Al toxicity has been shown to trigger a ROS burst in the root (Jones et al., 2006; Sivaguru et al., 2013), which could cause damage of essential root cellular components such as proteins, lipids, and DNA, leading to stunted root growth and function. Therefore, the ability to maintain proper balance of ROS generation by the network of ROS generating and scavenging enzymes could be a contributing factor to what is turning out to be a more complex set of processes underlying Al tolerance in sorghum. In previous RNAseq studies, genes involved in antioxidant activities were found to be up-regulated by Al stress in the root tip of buckwheat (Zhu et al., 2015) and hydrangea (Chen et al., 2015), two of the highly Al resistant plant species. In this study, our results indicated that a number of antioxidant enzymes in root tips were induced to much higher levels in SC566 than in BR007 (Table S5), which can explain why the Al sensitive lines accumulated much more potentially damaging ROS in root tips than did the Al tolerant line (Sivaguru et al., 2013). Interestingly, the STRING analysis also indicated that the up-regulated antioxidant proteins in SC566 showed strong interactions in response to Al stress (Figure 7B). Taken together, our results provide further evidence to support the importance of increased antioxidant activities in Al tolerance among different plant species.

### The root cell wall

Cell walls not only provide mechanical support for plant cells, but also play an important defensive role in stress adaptation (Wolf et al., 2012). The primary cell wall produced during cytokinesis consists of cellulose, hemicelluloses, and pectins (Keegstra, 2010), while the secondary wall, which is composed of cellulose, hemicellulose and lignin, serves to strengthen the primary cell wall (Wang et al., 2015). Lignin is made from three substrates (p-hydroxyphenyl, guaiacyl, and syringyl monolignols), all of which are synthesized through the phenylpropanoid pathway (Boerjan et al., 2003; Wang et al., 2015).

Several proteins involved in the phenylopropanoid biosynthetic pathway were significantly up-regulated in SC566 (Table 2). Further, analysis showed that although the lignin production was enhanced at 1D of Al treatment in the root tips of SC566, lignin in SC566 root tips went down to the control levels with longer Al treatments (3D and 5D; Figure 6A). In contrast, lignin levels were continuously increased in BR007 root tips over the entire Al exposure period (Figure 6B). Hyper-accumulation of lignin in roots can lead to stiffening of the root cell wall and inhibition of root elongation. In the rice root, it has been reported that an Al sensitive rice line accumulated more lignin than a tolerant rice line (Ma et al., 2012). Our results are consistent with the rice report and suggest that remodeling of the secondary cell wall could play a role in sorghum Al tolerance.

### Cell wall remodeling mediated by ROS generation

The formation of lignin polymers is also regulated by ROS production, especially by H_2_O_2_ and O^2−^, due to the tendency of ROS to form monolignol radical (Boerjan et al., 2003). Therefore, higher cellular ROS levels are well-known to promote lignin formation. It has been reported that increased levels of H_2_O_2_ led to enhanced lignin biosynthesis in rice (Kotula et al., 2009) and, in contrast, KI or CAT could reduce cell wall lignification *in vitro* (Lee et al., 2013). At the 1D of Al treatment, both of the tolerant and sensitive lines accumulated high levels of ROS in their root tip regions (Sivaguru et al., 2013). However, beginning from the 3D, the ROS level in the root tip region of the tolerant line was decreased to the level of the control, while the sensitive line remained high levels of ROS accumulation (Sivaguru et al., 2013). The dynamic changes in ROS accumulation in the root tip region could explain why at 1D, Al induced lignin production in the root tip of the tolerant line (Figure 6A). However, after 3D, lignin production decreased to the control level (Figure 6A). In contrast, lignin production in the sensitive line remained at high levels in the entire Al treatment process (Figure 6B). Thus, our results provide further evidence that levels of Al-induced ROS accumulation in sorghum roots could be a critical factor that causes root cell wall lignification and Al-induced reduction in root cell elongation in sorghum.

## Conclusion

Using a multi-channel iTRAQ technology, dynamic proteomic profile changes in the root tip region of two sorghum lines were studied. Our results suggest that in addition to the exclusion mechanism mediated by Al-activated root citrate exudation, antioxidant enzymes, and protein biosynthetic activities could play important roles for sorghum Al tolerance. In the Al tolerant SC566 line, the activities of multiple antioxidant enzymes, including SOD, POD, and CAT, were enhanced by Al stress, while no changes or suppression of activities of these enzymes were observed in root tips of BR007 treated with Al (Figure 5), which could contribute to higher levels of ROS accumulation in the root tips of Al sensitive BR007 (Sivaguru et al., 2013). As ROS is required for promoting lignin formation, the higher levels of ROS accumulation in the root tip region of BR007 under Al stress could also contribute to higher levels of lignin accumulation in the root tip region of BR007 (Figure 6). Hyper-accumulation of lignin could inhibit root elongation (Sasaki et al., 1996). In addition, it has been demonstrated that hemicellulose of cell walls is one of the Al-binding sites and higher levels of hemicellulose in roots are associated with higher levels of Al accumulation in root cell walls and higher levels of Al toxicity (Yang J. L. et al., 2011). Lignin could potentially be another Al binding substrate in the cell wall. It will be interesting to test if lignin could be another binding substrate for Al in future studies.

## Materials and methods

### Plant material, Al treatment, and measurement of relative daily root growth rates

Sorghum seeds were surface-sterilized with 0.5% (w/v) NaOCl for 15 min, rinsed with ultra-pure water and allowed to germinate in wet paper tower for 3 days at 26°C before the seedlings were transferred to Magnavaca hydroponic solution (pH 4.0) for a 1 day acclimation to low pH in a growth chamber with a temperature of 26°C, an illumination level of 240 μmol m^−2^ s^−1^, a 16 h photo period, and a relative humidity of 60–80%. Then the seedlings were transferred to fresh Magnavaca solutions with or without an Al^3+^ activity of 27 μM (pH 4.0) in the same growth chamber conditions, for 5 days (Magalhaes et al., 2007). Aluminum activity is referred to the free Al^3+^ concentration in the solution, which was predicted by the GEOCHEM-EZ program (Shaff et al., 2010).

Primary root length of each of 20 seedlings was measured every 24 h during the 0–5 days' Al treatment. Relative daily root growth rates were calculated as the ratios of the daily root growth under the Al treatment divided by the daily root growth under the −Al treatment. The root tip region (1–3 cm) of individual seedlings was collected after 0, 3, or 5 days of +/−Al treatment and the samples were frozen in liquid nitrogen and stored at −80°C for further studies.

### Proteomics workflow

The experiments consisted of four separate analyses, one for each cultivar and time point (Figure 2). Each of the analyses included three independent biological replicates for control and treatment of BR007 or SC566 as well as two replicates of a pooled internal standard. Each of the biological replicates included analyses of the proteins extracted from 200 mg (FW) of root tip tissues harvested from 20 individual BR007 or SC566 plants grown under the indicated conditions (see below). The internal standards were then constructed by pooling equal amounts of the extracted proteins from the control and treatment replicates. The protein extracts from the individual replicates and the internal standards were then separately digested with trypsin, creating a set of eight samples which were then labeled by iTRAQ with a randomized design, as specified in the inset table of Figure 2. After labeling, two sets of three biological replicates (three control replicates and three treatment replicates) and the two replicates of the internal standard were combined and subjected to high-pH reversed-phase (HpRP) chromatography to create a set of 48 first dimension fractions. These were then pooled strategically to create a set of 12 samples for further analyses by nanoLC-MS/MS.

### Protein extraction, digestion, and iTRAQ labeling

Sorghum proteins were extracted using the phenol extraction method as described (Yang Y. et al., 2011). Protein pellets were reconstituted in 7M Urea and 1% SDS. Protein concentrations were determined by Bradford assay using bovine serum albumin as a standard (Bradford, 1976). The quality of the protein preparation was further evaluated by running the samples in a precast NOVEX 12% Tris/Glycine mini-gel (Invitrogen, Carlsbad, CA) and the proteins were visualized with colloidal Coomassie blue stain (Invitrogen).

An aliquot (100 μg per sample) of proteins from each sample was digested and iTRAQ labeled following the manufacturer's recommended protocol with the following minor modifications (AB SCIEX, Framingham, MA, USA; Yang Y. et al., 2011). A total of 100 μg proteins of each sample were reduced by tris (2-carboxyethyl) phosphine (TCEP) and cysteine residues were blocked with 10 mM methyl methanethiosulfonate (MMTS). The modified proteins were reconstituted in 100 μL of 100 mM triethylammoniumbicarbonate after acetone precipitation. Each sample was digested independently with 10 μg trypsin and labeled with a unique iTRAQ label. Each set of iTRAQ 8-plex tags was used to label six samples (three controls and three treatments) and the remaining two tags were used to label two internal standards which were constructed by combining equal amounts of proteins from all the samples in the analysis (See Figure 2 for experimental design). Then, the eight labeled samples (three controls, three treatments, and two internal standards) of each of the four sets of analyses (2 cultivars × 2 time points) were pooled, followed by evaporation to dryness and cation exchange chromatography using a PolyLC strong cation-exchange cartridge (PolyLC Inc., Columbia, MD) to remove the SDS and subsequent desalt processes using RP-SPE on Sep-Pak® Cartridges (Waters, Milford, MA). The tryptic peptides were eluted in 50% CH3CN in 1% FA and evaporated to dryness.

### High pH reversed phase (HpRP) liquid chromatography

The HpRP chromatography was carried out by Ultra Performance Liquid Chromatography (UPLC). The peptide separation was accomplished using an Acquity UPLC System and UV detection (Waters, Milford, MA) coupled with a robotic fraction collector (Probot; Dionex, Sunnyvale, CA, USA) as reported previously (Okekeobu et al., 2014). Specifically, the iTRAQ 8-plex tagged tryptic peptides were reconstituted in buffer A (20 mM ammonium formate pH 9.5 in water), and half of the sample was loaded onto an Acquity UPLC BEH C18 column (1.7 μm, 2.1 × 100 mm, Waters, Milford, MA) with 20 mM ammonium formate (NH4FA), pH 9.5 as buffer A and 90% ACN/10% 20 mM NH4FA as buffer B. The separation was carried out using a gradient of 10–45% of buffer B in 12 min at a flow rate 200 μL/min. Forty-eight fractions were collected at 15 s intervals and pooled into a total of 12 samples based on the UV absorbance at 214 nm employing a multiple fraction concatenation strategy (Chen et al., 2013). All of the fractions were dried and reconstituted in 30 μL of 2% ACN/0.5% FA for nanoLC-MS/MS analysis.

### iTRAQ nanoflow LC-MS/MS analysis

The nanoLC-MS/MS analysis was carried out using an Orbitrap Elite mass spectrometer (Thermo-Fisher Scientific, San Jose, CA) equipped with nano ion source using high energy collision dissociation (HCD) similar to previous reports (Yang Y. et al., 2011). The Orbitrap is coupled with the UltiMate3000 RSLCnano (Dionex, Sunnyvale, CA). Each reconstituted fraction (5 μL) was injected Into a PepMap C-18 RP nano trap column (3, 75, 20 mm, Dionex) with nanoViper Fittings at 20 μL/min flow rate for on-line concentration and desalting and then separated on a PepMap C-18 RP nano column (3 μm, 75 × 15 cm), and eluted in a 120 min gradient of 5–38% acetonitrile (ACN) in 0.1% formic acid at 300 nL/min, followed by a 5-min ramp to 95% ACN-0.1% FA and a 7 min hold at 95% ACN-0.1% FA. The column was re-equilibrated with 2% ACN-0.1% FA for 20 min prior to the next run. The Orbitrap Elite is operated in positive ion mode with nano spray voltage set at 1.6 kV and source temperature at 275°C. The instrument was externally calibrated using Ultramark 1621 for the FT mass analyzer. An internal calibration was performed using the background polysiloxane ion signal at m/z 445.120025 as the calibrant. The instrument was operated in data-dependent acquisition (DDA) mode. In all experiments, full MS scans were acquired over a mass range of m/z 400–1400, with detection in the Orbitrap mass analyzer at a resolution setting of 60,000 (FWHM). Fragment ion spectra produced via HCD were acquired in the Orbitrap mass analyzer with a resolution setting of 15,000 for the mass range of m/z 100–2000. In each cycle of DDA analysis, following each survey scan, the 20 most intense multiply charged ions above a threshold ion count of 5000 were selected for fragmentation at a normalized collision energy of 45%. Dynamic exclusion parameters were set at repeat count 1 with a 30 s repeat duration, an exclusion list size of 500, 30 s exclusion duration with ±10 ppm exclusion mass window. The activation time was 0.1 ms for HCD analysis. All data were acquired with Xcalibur 2.2 software (Thermo-Fisher Scientific).

### Data processing, protein identification, and data analysis

All MS and MS/MS raw spectra from iTRAQ experiments were processed using Proteome Discoverer 1.4 (PD1.4, Thermo) and the spectra from each DDA file were output as an MGF file for subsequent database searches using a licensed copy of Mascot Daemon (version 2.5.1, Matrix Science, Boston, MA). The *Sorghum bicolor* protein RefSeq sequence database containing 75,079 sequence entries was downloaded on 9 Dec, 2014 from NCBInr and used for database searches. The default search settings used for 8-plex iTRAQ quantitative processing and protein identification in Mascot server were as described previously (Yang Y. et al., 2011; Okekeobu et al., 2014). In brief, two miss-cleavage for full trypsin digestions were conducted with fixed methylthio modification of cysteine, fixed 8-plex iTRAQ modifications on lysine and N-terminal amines and variable modifications of methionine oxidation and deamidation on asparagines/glutamine residues. The peptide mass tolerance and fragment mass tolerance values were 10 ppm and 0.2 Da, respectively.

The resulting peptides were considered to be confidently-identified peptides and used for protein identifications. Furthermore, proteins identified in all eight iTRAQ experiments, which contained at least two peptides with a *p*-value of <0.05 (expectation value) as determined by Mascot probability analysis were considered confidently identified and further analyzed. Intensities of the reporter ions generated from the iTRAQ tags upon fragmentation were used for quantification, and the relative quantitation ratios were normalized to median ratio in each set of experiments. To estimate the false discovery rate (FDR), an automatic decoy database search was performed in Mascot by choosing the decoy checkbox in which a random sequence of database is generated and tested for raw spectra as well as the real database. The FDRs% for sets 1, 2, 3, and 4 were 0.70, 0.70, 0.68, and 0.68%, respectively.

### Functional classification by gene ontology

Candidate genes were classified with the online software blast2GO (http://www.blast2go.com) and Agrigo (http://bioinfo.cau.edu.cn/agriGO/; Du et al., 2010). Protein pathway analysis was performed using KEGG (http://www.genome.jp/kegg/). The protein-protein interaction was analyzed with STRING (http://string-db.org/).

### Enzyme activity test

Root tip samples (0.5 g fresh weight) were homogenized in 10 ml of the HEPES-KOH buffer (pH 7.8) containing 0.1 mM EDTA. The homogenate was centrifuged at 15,000 g for 15 min. All operations were performed at 4°C. The supernatant of each sample was used for the following enzymatic assays:

**Superoxide dismutase (SOD)** was assayed by a photochemical method as described (Giannopolitis and Ries, 1977). Reaction mixtures (3 ml) consisted of 50 mM HEPES-KOH (pH 7.8), 0.1 mM EDTA, 50 mM Na_2_CO_3_ (pH10.2), 12 mM L-methionine, 75 μM NBT, appropriately diluted enzyme extracts (0–300 μl) and 1 μM riboflavin. One unit SOD activity was defined as the amount of enzyme required to result in a 50% inhibition of the rate of NBT reduction measured at 560 nM with a time interval of 15 min.
**Peroxidase (POD) activity determination**. The reaction mixture consisted of 25 mM potassium phosphate buffer (pH 6.8), 10 mM H_2_O_2_, 0.05% guaiacol, and 100 μl the diluted enzyme extract. The oxidation of guaiacol (extinction coefficient 26.6 M^−1^cm^−1^) was followed at 470 nM and the obtained values were recorded every min. Protein content was measured by the Bradford (Bradford, 1976). The results were presented as oxidation guaiacol (O.G) mmol per g protein per minute.
**CAT activity determination**. The reaction mixture consisted of 25 mM potassium phosphate buffer (pH6.8), 10 mM H_2_O_2_ and the diluted enzyme extract in a total volume of 1 ml. The decomposition of H_2_O_2_ (extinction coefficient 39.4 M^−1^cm^−1^) was followed by the decline in absorbance at 240 nM within 1 min as described (Aebi, 1984). The CAT activity was expressed as H_2_O_2_ mmol per g protein per minute.

### Determination of lignin content

Root tip tissues were homogenized in 95% ethanol. After centrifugation at 1000 g for 5 min, the pellet was washed three times with 95% ethanol and twice with ethanol-hexane (1:2, v/v). The washed pellet was allowed to air-dry. The lignin content was determined according to the method of Morrison with some modifications (Morrison, 1972). After washing with 25% acetyl bromide in glacial acetic acid, the samples (~10 mg) were incubated at 70°C for 30 min in glass-stoppered test tubes with 1 ml 25% acetyl bromide in glacial acetic acid. Then, 0.9 ml of 2M NaOH, 5 ml of glacial acetic acid, and 0.1 ml of 7.5 M hydroxylamine hydrochloride were added to the tube, and the volume was made up to 10 ml with glacial acetic acid. After centrifugation at 1000 g for 5 min, the absorbance of the supernatant was measured at 280 nm to determine the lignin content. There was no interference from protein in the measurement of the lignin content at A280, because protein was precipitated by the procedure used.

## Author contributions

DZ, YY, JZ, and JL designed the experiments; DZ, YY, TT, LK, and JL participated in data analysis and wrote the article; DZ, YY, JZ, FJ, and EC performed the research.

### Conflict of interest statement

The authors declare that the research was conducted in the absence of any commercial or financial relationships that could be construed as a potential conflict of interest.

## Supplementary material

Supplemental Data 1 **Proteome profiling**.

Table S1 **Differentially expressed proteins (fold changes) in 3D BR007**.

Table S2 **Differentially expressed proteins (fold changes) in 5D BR007**.

Table S3 **Differentially expressed proteins (fold changes) in 3D SC566**.

Table S4 **Differentially expressed proteins (fold changes) in 5D SC566**.

Table S5 **Relative protein expression (fold changes) of antioxidant enzymes in BR007 and SC566 under Al treatment**.

Figure S1 **Key metabolic pathways for key proteins involved in Al tolerance**. A total of nine peroxidases with >1.5-fold upregulation were found in the phenylpropanoid biosynthesis pathway in BR007 5D.

Figure S2 **Key differentially expressed proteins in metabolism of xenobiotics by cytochrome P450 involved in Al tolerance**.

## Abbreviations

Al: aluminum
ALMT: Al-activated malate transporter
CAT: catalase
DDA: data-dependent acquisition
DEP: differentially expressed protein
HCD: high energy collision dissociation
HpRP: high pH reversed phase
MATE: multidrug and toxic compound extrusion
OA: organic acid
POD: peroxidase
SOD: superoxide dismutase
2D-LC: two-dimensional liquid chromatography
UPLC: ultra performance liquid chromatography.
