# Supplementary material for: Quantitative iTRAQ Proteomics Revealed Possible Roles for Antioxidant Proteins in Sorghum Aluminum Tolerance
Source: Front Plant Sci. 2017 Jan 9;7:2043. doi: 10.3389/fpls.2016.02043 (PMC5220100; doi:10.3389/fpls.2016.02043)
Supplement: Table S1 — Differentially expressed proteins (fold changes) in 3D BR007. [file Table1.PDF]

Table S1. Differentially Expressed Proteins (Fold Change) in 3D BR007.

Protein ID	Protein Description	Relative Protein Expression[1]	Variance[2]	SE[3]
gi|613447702	XRN4 - exoribonuclease 4; Possesses 5'->3' exoribonuclease activity. Acts as an endogenous post-transcriptional gene silencing (PTGS) suppressor. Degrades miRNA target cleavage products that lack a 5'-cap structure. Antagonizes the negative feedback regulation on EIN3 by promoting EBF1 and EBF2 mRNA decay, which consequently allows the accumulation of EIN3 protein to trigger the ethylene response	13.39	15.02	2.24
gi|241933790	AT2G24600 - ankyrin repeat-containing protein	10.41	31.20	3.22
gi|241929418	LCR83 - putative defensin-like protein 70	7.63	0.21	0.27
gi|241936642	SERK2 - somatic embryogenesis receptor kinase 2; Serine/threonine-kinase involved in brassinosteroid- dependent and -independent signaling pathways. Acts redundantly with SERK1 as a control point for sporophytic development controlling male gametophyte production	6.79	1.99	0.81
gi|241946897	BCS1 - cytochrome BC1 synthesis	6.60	24.40	2.85
gi|241933962	HTA11 - histone H2A; Variant histone H2A which may replace conventional H2A in a subset of nucleosomes. Nucleosomes wrap and compact DNA into chromatin, limiting DNA accessibility to the cellular machineries which require DNA as a template. Histones thereby play a central role in transcription regulation, DNA repair, DNA replication and chromosomal	5.31	0.92	0.55

	stability. DNA accessibility is regulated via a complex set of post-translational modifications of histones, also called histone code, and nucleosome remodeling (By similarity)			
gi|241929783	AT3G05545 - RING/U-box domain-containing protein	5.10	0.91	0.55
gi|241927971	CYP72A15 - cytochrome P450, family 72, subfamily A, polypeptide 15	4.75	0.14	0.22
gi|241930936	cICDH - isocitrate dehydrogenase; May supply 2-oxoglutarate for amino acid biosynthesis and ammonia assimilation via the glutamine synthetase/glutamate synthase (GS/GOGAT) pathway. May be involved in the production of NADPH to promote redox signaling or homeostasis in response to oxidative stress	4.58	0.41	0.37
gi|241921461	GSTU18 - glutathione S-transferase TAU 18; May be involved in the conjugation of reduced glutathione to a wide number of exogenous and endogenous hydrophobic electrophiles and have a detoxification role against certain herbicides (By similarity)	4.11	0.16	0.23
gi|241929511	AT1G13930 - uncharacterized protein	4.08	0.50	0.41
gi|241920881	AT2G15220 - basic secretory protein family protein	4.02	0.24	0.28
gi|241946622	UGT73B3 - UDP-glucosyl transferase 73B3; Possesses quercetin 3-O-glucosyltransferase activity in vitro. Also active in vitro on benzoates and benzoate derivatives. Involved in stress or defense responses	3.84	0.36	0.35
gi|241918016	AGO10 - ARGONAUTE 10; Involved in RNA-mediated post-transcriptional gene silencing (PTGS). Main component of the RNA-induced silencing complex (RISC) that binds to a short	3.78	1.41	0.68

	guide RNA such as a microRNA (miRNA) or small interfering RNA (siRNA). RISC uses the mature miRNA or siRNA as a guide for slicer-directed cleavage of homologous mRNAs to repress gene expression. Required for reliable formation of primary and axillary shoot apical meristems. Specifies leaf adaxial identity by repressing the miR165 and miR166 microRNAs in the embryonic shoot apex, in the shoot apical meristem (SAM) and leaf. R [...]			
gi|241918709	AT4G25030 - uncharacterized protein	3.59	2.38	0.89
gi|241930176	CYP72A15 - cytochrome P450, family 72, subfamily A, polypeptide 15	3.51	0.21	0.26
gi|241922789	AT3G22600 - bifunctional inhibitor/lipid-transfer protein/seed storage 2S albumin-like protein	3.49	0.15	0.23
gi|241926418	AT4G19900 - alpha 1,4-glycosyltransferase-like protein	3.45	0.36	0.34
gi|241920119	AATP1 - AAA-ATPase 1	3.41	0.11	0.19
gi|241922980	LCR69 - defensin-like protein 2; Confers broad-spectrum resistance to pathogens	3.41	0.17	0.24
gi|77744849	TIL - temperature-induced lipocalin	3.25	0.10	0.18
gi|241914611	AT1G76940 - RNA recognition motif-containing protein	3.23	0.12	0.20
gi|241932602	COR47 - dehydrin COR47	3.14	0.12	0.20
gi|241915454	AT5G11330 - FAD/NAD(P)-binding oxidoreductase family protein	3.13	1.11	0.61
gi|241926633	AT1G14550 - peroxidase 5; Removal of H(2)O(2), oxidation of toxic reductants, biosynthesis and degradation of lignin, suberization, auxin catabolism, response to environmental stresses such as wounding, pathogen attack and oxidative stress. These	3.11	0.37	0.35

	functions might be dependent on each isozyme/isoform in each plant tissue			
gi|241930937	PEX6 - peroxin 6; Involved in peroxisomal-targeting signal one (PTS1) and peroxisomal-targeting signal two (PTS2) protein import. Required for jasmonate biosynthesis. Necessary for the developmental elimination of obsolete peroxisome matix proteins. May form heteromeric AAA ATPase complexes required for the import of proteins. May be involved in PEX5 recycling	3.10	0.02	0.09
gi|241924606	EULS3 - Euonymus lectin S3	3.09	0.06	0.14
gi|241931078	GSTU7 - glutathione S-transferase tau 7; May be involved in the conjugation of reduced glutathione to a wide number of exogenous and endogenous hydrophobic electrophiles and have a detoxification role against certain herbicides (By similarity)	3.08	0.13	0.21
gi|241916274	OPR1 - 12-oxophytodienoate reductase 1; Specifically cleaves olefinic bonds in alpha,beta- unsaturated carbonyls and may be involved in detoxification or modification of these reactive compounds. May be involved in the biosynthesis or metabolism of oxylipin signaling molecules. In vitro, reduces 9R,13R-12-oxophyodienoic acid (9R,13R-OPDA) to 9R,13R-OPC-8:0, but only poorly 9S,13S-OPDA, the natural precursor of jasmonic acid. Can detoxify the explosive 2,4,6-trinitrotoluene (TNT) in vitro and in vivo by catalyzing its nitroreduction to form hydroxylamino-dinitrotoluene (HADNT)	3.03	0.90	0.55

gi|241942022	WAT1 - Walls Are Thin 1; Required for secondary wall formation in fibers, especially in short days conditions. Promotes indole metabolism and transport (e.g. tryptophan, neoglucobrassicin and auxin (indole-3-acetic acid)). May prevent salicylic-acid (SA) accumulation	3.03	0.20	0.26
gi|241943349	AT3G09510 - RNase H domain-containing protein	2.96	0.48	0.40
gi|241915136	AT3G12710 - DNA-3-methyladenine glycosylase I	2.95	0.47	0.40
gi|241922218	AT1G28200.1 - GEM-like protein 1	2.80	0.13	0.21
gi|241921870	LAC12 - laccase 12; Lignin degradation and detoxification of lignin-derived products (By similarity)	2.71	0.37	0.35
gi|241943862	AT5G13200 - GEM-like protein 5	2.70	0.16	0.23
gi|15529117	EP3 - chitinase	2.70	0.04	0.12
gi|241916320	AT3G19740 - P-loop containing nucleoside triphosphate hydrolase domain-containing protein	2.68	3.28	1.04
gi|22208467	UGT73C7 - UDP-glucosyl transferase 73C7	2.67	0.06	0.14
gi|241947322	HCHIB - chitinase; Defense against chitin containing fungal pathogens. Seems particularly implicated in resistance to jasmonate-inducing pathogens such as A.brassicicola. In vitro antifungal activity against T.reesei, but not against A.solani, F.oxysporum, S.sclerotiorum, G.graminis and P.megasperma	2.66	0.13	0.21
gi|241945753	AT5G48540 - cysteine-rich repeat secretory protein 55	2.65	0.10	0.18
gi|241917978	AT1G60995 - uncharacterized protein	2.65	0.86	0.54
gi|241935663	ARPN - plantacyanin; Forms a concentration gradient along the pollen tube growth path, with	2.65	0.17	0.24

	a lower level in the stigma papilla cell wall and a higher level in the transmitting tract extracellular matix of the style			
gi|241945166	AT3G28500 - 60S acidic ribosomal protein P2-3; Plays an important role in the elongation step of protein synthesis (By similarity)	2.64	0.02	0.08
gi|241926310	BCS1 - cytochrome BC1 synthesis	2.63	0.06	0.14
gi|241914792	AT2G19750 - 40S ribosomal protein S30	2.62	0.59	0.44
gi|241936501	AT4G35160 - O-methyltransferase family 2 protein	2.62	0.13	0.21
gi|241931342	GG1 - Ggamma-subunit 1; Guanine nucleotide-binding proteins (G proteins) are involved as a modulator or transducer in various transmembrane signaling systems. The beta and gamma chains are required for the GTPase activity, for replacement of GDP by GTP, and for G protein- effector interaction. Involved in the abscisic acid (ABA) and ethylene signaling pathways. Regulates acropetal transport of auxin (IAA) in roots and hypocotyls, and thus modulates root architecture (e.g. lateral root formation). The heterotrimeric G- protein controls defense responses to necrotrophic and vascular fungi prob [...]	2.62	1.21	0.64
gi|241932218	AT1G67900 - phototropic-responsive NPH3-like protein; May act as a substrate-specific adapter of an E3 ubiquitin-protein ligase complex (CUL3-RBX1-BTB) which mediates the ubiquitination and subsequent proteasomal degradation of target proteins (By similarity)	2.62	0.24	0.28
gi|241918978	SP1L1 - SPIRAL1-like1; Acts redundantly with SPR1 in maintaining the cortical microtubules	2.60	0.20	0.26

	organization essential for anisotropic cell growth (By similarity)			
gi|241945749	AT4G32285 - putative clathrin assembly protein	2.58	0.03	0.10
gi|241935615	PR4 - hevein-like protein; Fungal growth inhibitors. Neither CB-HEL nor CD-HEL have chitinase activity, but both have antimicrobial activities. CD-HEL has RNase, but no DNase activity	2.58	0.02	0.08
gi|241933008	CAT1 - catalase 1; Occurs in almost all aerobically respiring organisms and serves to protect cells from the toxic effects of hydrogen peroxide (By similarity)	2.56	0.07	0.16
gi|241928175	ABCB11 - P-glycoprotein 11	2.55	0.04	0.11
gi|241921563	AT1G60420 - putative nucleoredoxin 1; Probable thiol-disulfide oxidoreductase required for pollen tube growth and pollen function in the pistil. Seems not to be required for in vitro pollen tube growth. May be involved in the generation of lipid signaling molecules in pistil	2.55	0.05	0.13
gi|241931024	MES1 - methyl esterase 1; Methylesterase shown to have carboxylesterase activity, methyl indole-3-acetic acid (MeIAA) esterase activity, methyl salicylate (MeSA) esterase activity and methyl jasmonate (MeJA) esterase activity in vitro. Required to convert methyl salicylate (MeSA) to salicylic acid (SA) as part of the signal transduction pathways that activate systemic acquired resistance in systemic tissue. MeSA is believed to be an inactive form that needs to be demethylated to exert a biological effect	2.52	0.02	0.08
gi|241919322	HSC70-1 - heat shock 70kDa protein 1/8; Component of the Mediator complex, a	2.52	0.04	0.12

	coactivator involved in the regulated transcription of nearly all RNA polymerase II-dependent genes. Mediator functions as a bridge to convey information from gene-specific regulatory proteins to the basal RNA polymerase II transcription machinery. The Mediator complex, having a compact conformation in its free form, is recruited to promoters by direct interactions with regulatory proteins and serves for the assembly of a functional preinitiation complex with RNA polymerase II and the general transcription factors (By s [...]			
gi|241929989	THI1 - thiazole biosynthetic enzyme; Involved in biosynthesis of the thiamine precursor thiazole. Catalyzes the conversion of NAD and glycine to adenosine diphosphate 5-(2-hydroxyethyl)-4-methylthiazole-2-carboxylic acid (ADT), an adenylated thiazole intermediate. The reaction includes an iron-dependent sulfide transfer from a conserved cysteine residue of the protein to a thiazole intermediate. The enzyme can only undergo a single turnover, which suggests it is a suicide enzyme. May have additional roles in adaptation to various stress conditions and in DNA damage tolerance	2.52	0.08	0.16
gi|241929604	CYSB - cystatin B; Specific inhibitor of cysteine proteinases. Probably involved in the regulation of endogenous processes and in defense against pests and pathogens (By similarity)	2.50	0.03	0.11
gi|241916095	AT3G02645 - uncharacterized protein	2.49	0.10	0.18
gi|241922385	AT5G60710 - C3H4 type zinc finger protein	2.49	0.03	0.09

gi|241935975	PRX52 - peroxidase 52; Removal of H(2)O(2), oxidation of toxic reductants, biosynthesis and degradation of lignin, suberization, auxin catabolism, response to environmental stresses such as wounding, pathogen attack and oxidative stress. These functions might be dependent on each isozyme/isoform in each plant tissue	2.49	0.06	0.15
gi|241917737	RS27A - ribosomal protein S27; May be involved in the elimination of damaged mRNA after UV irradiation	2.48	0.19	0.25
gi|257659117	PPDK - pyruvate, phosphate dikinase 1; Formation of phosphoenolpyruvate. May be involved in regulating the flux of carbon into starch and fatty acids of seeds and in the remobilization of nitrogen reserves in senescing leaves	2.46	0.01	0.07
gi|241937749	AT2G03200 - aspartyl protease-like protein	2.45	0.19	0.25
gi|241921462	GSTU18 - glutathione S-transferase TAU 18; May be involved in the conjugation of reduced glutathione to a wide number of exogenous and endogenous hydrophobic electrophiles and have a detoxification role against certain herbicides (By similarity)	2.41	0.00	0.04
gi|241946533	LSH5 - uncharacterized protein	2.41	0.10	0.18
gi|241931083	GLP5 - germin-like protein 5; May play a role in plant defense. Probably has no oxalate oxidase activity even if the active site is conserved	2.40	0.16	0.23
gi|241926632	RCI3 - peroxidase 3; Removal of H(2)O(2), oxidation of toxic reductants, biosynthesis and degradation of lignin, suberization, auxin catabolism, response to environmental stresses such as wounding, pathogen attack and	2.40	0.02	0.08

	oxidative stress. These functions might be dependent on each isozyme/isoform in each plant tissue			
gi|241939895	FRUCT5 - beta-fructofuranosidase 5; 6-fructan exohydrolase that can use phlein, levan, neokestose, levanbiose, 6-kestose, and 1-kestose as substrates	2.40	0.04	0.11
gi|241918039	AT4G31360 - selenium binding protein	2.38	0.19	0.25
gi|241939010	AT1G20110 - RING/FYVE/PHD zinc finger-containing protein	2.37	0.02	0.08
gi|241942264	AT3G12640 - RNA binding (RRM/RBD/RNP motifs) family protein	2.35	1.05	0.59
gi|219965357	BTI1 - reticulon-like protein B1; Plays a role in the Agrobacterium-mediated plant transformation via its interaction with VirB2, the major component of the T-pilus	2.35	0.01	0.07
gi|241935380	AT3G48770 - ATP/DNA binding protein	2.34	0.11	0.19
gi|241930137	AT1G60690 - NAD(P)-linked oxidoreductase-like protein	2.34	0.01	0.07
gi|241923071	AT3G48140 - B12D protein	2.34	0.04	0.12
gi|241922107	RD20 - caleosin-related protein; Probable calcium-binding peroxygenase. May be involved in the degradation of storage lipid in oil bodies, in abiotic stress-related signaling pathway and in drought tolerance through stomatal control under water deficit conditions	2.34	0.19	0.25
gi|241928420	SC3 - secretory carrier 3; Probably involved in membrane trafficking (By similarity)	2.33	0.20	0.26
gi|241915296	AT5G24165 - uncharacterized protein	2.33	0.05	0.13
gi|241935263	OMT1 - O-methyltransferase 1; Methylates OH residues of flavonoid compounds. Converts quercetin into isorhamnetin. Dihydroquercetin is not a substrate. Catalyzes the methylation of	2.31	0.78	0.51

	monolignols, the lignin precursors. Does not contribute to the phenylpropanoid pattern of the pollen tryphine, but is probably confined to isorhamnetin gylcoside biosynthesis			
gi|241926030	AT5G47530 - putative auxin-responsive protein	2.30	0.08	0.16
gi|241926454	OSM34 - osmotin 34	2.30	0.01	0.05
gi|241925310	PIP2B - aquaporin PIP2-2; Water channel required to facilitate the transport of water across cell membrane. Plays an predominant role in root water uptake process in conditions of reduced transpiration, and in osmotic fluid transport. Its function is impaired by Hg(2+). Inhibited by cytosolic acidosis which occurs during anoxia in roots	2.29	0.04	0.11
gi|241942135	AT5G61820 - uncharacterized protein	2.27	0.00	0.03
gi|241927309	WRKY26 - WRKY DNA-binding protein 26; Transcription factor. Interacts specifically with the W box (5'-(T)TGAC[CT]-3'), a frequently occurring elicitor- responsive cis-acting element (By similarity)	2.26	0.24	0.28
gi|241935976	PRX52 - peroxidase 52; Removal of H(2)O(2), oxidation of toxic reductants, biosynthesis and degradation of lignin, suberization, auxin catabolism, response to environmental stresses such as wounding, pathogen attack and oxidative stress. These functions might be dependent on each isozyme/isoform in each plant tissue	2.26	0.08	0.16
gi|241928000	UGT73B4 - UDP-glycosyltransferase 73B4; Possesses quercetin 3-O-glucosyltransferase and low 7-O- glucosyltransferase activities in vitro. Also active in vitro on benzoates and benzoate derivatives. Can detoxify the	2.26	1.27	0.65

	explosive 2,4,6-trinitrotoluene in plant by forming O- or C-glucose conjugates			
gi|241945724	UPI - UNUSUAL SERINE PROTEASE INHIBITOR	2.25	0.04	0.12
gi|241923908	UGT74F2 - UDP-glucosyltransferase 74F2; Glycosyltransferase that glucosylates benzoic acid and derivatives. Substrate preference is benzoic acid > salicylic acid (SA) > 3-hydroxybenzoic acid > 4-hydroxybenzoic acid. Catalyzes the formation of both SA 2-O-beta-D-glucoside (SAG) and SA glucose ester (SGE). Has high affinity for the tryptophan precursor anthranilate. Catalyzes the formation of anthranilate glucose ester. Is the major source of this activity in the plant	2.25	0.04	0.11
gi|241938127	XRN4 - exoribonuclease 4; Possesses 5'->3' exoribonuclease activity. Acts as an endogenous post-transcriptional gene silencing (PTGS) suppressor. Degrades miRNA target cleavage products that lack a 5'-cap structure. Antagonizes the negative feedback regulation on EIN3 by promoting EBF1 and EBF2 mRNA decay, which consequently allows the accumulation of EIN3 protein to trigger the ethylene response	2.24	0.01	0.05
gi|241917393	AT1G27400 - 60S ribosomal protein L17-1	2.24	0.30	0.32
gi|241937907	AT5G11420 - uncharacterized protein	2.22	0.04	0.11
gi|241933461	AT3G09620 - DEAD-box ATP-dependent RNA helicase 45	2.21	0.33	0.33
gi|241927406	AT3G56740 - Ubiquitin-associated (UBA) protein	2.19	0.04	0.11
gi|241917045	BPM2 - BTB-POZ and MATH domain 2; May act as a substrate-specific adapter of an E3	2.18	0.06	0.14

	ubiquitin-protein ligase complex (CUL3-RBX1-BTB) which mediates the ubiquitination and subsequent proteasomal degradation of target proteins			
gi|241919705	ASL15 - ASYMMETRIC LEAVES 2-like 15	2.18	0.39	0.36
gi|241930740	AT5G05960 - bifunctional inhibitor/lipid-transfer protein/seed storage 2S albumin-like protein	2.18	0.11	0.19
gi|241937766	AT2G14095 - uncharacterized protein	2.17	0.17	0.24
gi|241923012	CESA6 - cellulose synthase 6; Catalytic subunit of cellulose synthase terminal complexes ('rosettes'), required for beta-1,4-glucan microfibril crystallization, a major mechanism of the cell wall formation. Involved in the primary cell wall formation. The presence of each protein CESA1 and CESA6 is critical for cell expansion. The hypocotyl elongation is based on a CESA6-dependent cell elongation in dark and a CESA6-independent cell elongation in light. The transition between these two mechanisms requires photosynthesis and PHYB, but not CRY1. The CESA6-dependent cell elongation seems to be in [...]	2.16	0.08	0.17
gi|241942939	SYP131 - syntaxin 1B/2/3; Vesicle trafficking protein that functions in the secretory pathway (By similarity)	2.15	0.01	0.04
gi|241926425	FLR1 - FLOR1	2.15	0.05	0.13
gi|241930111	ABCG40 - ATP-binding cassette G40; May be a general defense protein (By similarity). Functions as a pump to exclude Pb(2+) ions and/or Pb(2+)- containing toxic compounds from the cytoplasm. Contributes to Pb(2+) ions resistance. Confers some resistance to the terpene sclareol	2.14	0.09	0.17

gi|241920099	AT3G57490 - 40S ribosomal protein S2-4	2.14	0.19	0.25
gi|241925556	PPC1 - phosphoenolpyruvate carboxylase 1; Through the carboxylation of phosphoenolpyruvate (PEP) it forms oxaloacetate, a four-carbon dicarboxylic acid source for the tricarboxylic acid cycle. Contributes probably to the adaptation to inorganic phosophate (Pi) deprivation	2.13	0.02	0.09
gi|241917923	AT5G09570 - Cox19-like CHCH family protein	2.12	0.13	0.21
gi|241928915	GLP5 - germin-like protein 5; May play a role in plant defense. Probably has no oxalate oxidase activity even if the active site is conserved	2.11	0.28	0.31
gi|241943924	CYP71B10 - cytochrome P450 71B10	2.11	0.36	0.35
gi|241921463	GSTU18 - glutathione S-transferase TAU 18; May be involved in the conjugation of reduced glutathione to a wide number of exogenous and endogenous hydrophobic electrophiles and have a detoxification role against certain herbicides (By similarity)	2.10	0.01	0.06
gi|241941650	AT3G21215 - RNA recognition motif-containing protein	2.10	0.14	0.22
gi|241926637	PA2 - peroxidase 2; Removal of H(2)O(2), oxidation of toxic reductants, biosynthesis and degradation of lignin, suberization, auxin catabolism, response to environmental stresses such as wounding, pathogen attack and oxidative stress. These functions might be dependent on each isozyme/isoform in each plant tissue	2.09	0.29	0.31
gi|241930087	AT4G17520 - plasminogen activator inhibitor 1 RNA-binding protein	2.09	0.03	0.10
gi|241943041	AT2G28790 - pathogenesis-related thaumatin-like protein	2.08	0.01	0.07

gi|241921913	AT2G37290 - RabGAP/TBC domain-containing protein	2.08	0.44	0.38
gi|241918805	EXPB2 - expansin B2; May cause loosening and extension of plant cell walls by disrupting non-covalent bonding between cellulose microfibrils and matrix glucans. No enzymatic activity has been found (By similarity)	2.07	0.03	0.10
gi|241925690	AT5G15870 - glycosyl hydrolase family 81 protein	2.07	0.02	0.08
gi|241936021	AT1G50180 - NB-ARC domain-containing disease resistance protein; Potential disease resistance protein (By similarity)	2.06	0.17	0.24
gi|241931072	AT1G24620 - putative calcium-binding protein CML25; Potential calcium sensor (By similarity)	2.06	0.30	0.32
gi|4680212	SP1L2 - SPIRAL1-like2; Acts redundantly with SPR1 in maintaining the cortical microtubules organization essential for anisotropic cell growth	2.05	0.03	0.10
gi|241920098	AT3G57490 - 40S ribosomal protein S2-4	2.04	0.90	0.55
gi|241918213	CYP76C4 - cytochrome P450 76C4	2.04	0.31	0.32
gi|241915746	EXO70G1 - exocyst complex component 7	2.04	0.04	0.11
gi|241928202	AT2G19730 - 60S ribosomal protein L28-1	2.03	0.07	0.15
gi|241917092	AT4G31200 - SWAP/Surp domain-containing protein	2.03	0.11	0.19
gi|241924332	GSTU25 - glutathione S-transferase TAU 25; May be involved in the conjugation of reduced glutathione to a wide number of exogenous and endogenous hydrophobic electrophiles and have a detoxification role against certain herbicides (By similarity)	2.02	0.18	0.25
gi|241937783	PLAT1 - PLAT domain protein 1	2.02	0.05	0.13
gi|241946443	AT1G71950 - Proteinase inhibitor, propeptide	2.01	0.01	0.07

gi|241932928	SUS6 - sucrose synthase 6; Sucrose-cleaving enzyme that provides UDP-glucose and fructose for various metabolic pathways. Functions in callose synthesis at the site of phloem sieve elements	2.01	0.02	0.08
gi|241918333	AT4G09770 - TRAF-like family protein	2.01	0.02	0.07
gi|241921616	AT1G22410 - class-II DAHP synthetase-like protein	2.00	0.07	0.15
gi|241922091	AOS - allene oxide synthase	2.00	0.03	0.11
gi|241918870	PUB13 - plant U-box 13; Functions as an E3 ubiquitin ligase (By similarity)	1.99	0.07	0.15
gi|241945076	AT4G02880 - uncharacterized protein	1.99	0.08	0.16
gi|241928586	HSP70 - heat shock protein 70; Component of the Mediator complex, a coactivator involved in the regulated transcription of nearly all RNA polymerase II-dependent genes. Mediator functions as a bridge to convey information from gene-specific regulatory proteins to the basal RNA polymerase II transcription machinery. The Mediator complex, having a compact conformation in its free form, is recruited to promoters by direct interactions with regulatory proteins and serves for the assembly of a functional preinitiation complex with RNA polymerase II and the general transcription factors (By similarity)	1.98	0.16	0.23
gi|241940003	AT1G71695 - peroxidase 12; Removal of H(2)O(2), oxidation of toxic reductants, biosynthesis and degradation of lignin, suberization, auxin catabolism, response to environmental stresses such as wounding, pathogen attack and oxidative stress. These	1.98	0.02	0.08

	functions might be dependent on each isozyme/isoform in each plant tissue			
gi|21326114	ABCB27 - ATP-binding cassette B27; Probably involved in redistribution of internalized aluminum. May mediate vacuolar sequestration of a metal complex	1.97	0.14	0.21
gi|241926142	AT5G11880 - diaminopimelate decarboxylase 2; Specifically catalyzes the decarboxylation of meso- diaminopimelate (meso-DAP) to L-lysine	1.97	0.26	0.30
gi|241936498	AT1G58170 - Disease resistance-responsive (dirigent-like protein) family protein	1.96	0.02	0.08
gi|241938193	ABCC9 - ATP-binding cassette C9; Pump for glutathione S-conjugates (By similarity)	1.96	0.12	0.20
gi|241919699	PHT1;7 - phosphate transporter 1;7; High-affinity transporter for external inorganic phosphate (By similarity)	1.96	0.01	0.07
gi|241938280	AGO7 - ARGONAUTE7; Involved in RNA-mediated post-transcriptional gene silencing (PTGS). Main component of the RNA-induced silencing complex (RISC) that binds to a short guide RNA such as a microRNA (miRNA) or small interfering RNA (siRNA). RISC uses the mature miRNA or siRNA as a guide for slicer-directed cleavage of homologous mRNAs to repress gene expression. Required for the processing of 21 nucleotide trans-acting siRNAs (ta-siRNAs) derived from TAS3a transcripts. Associates preferentially with the microRNA (miRNA) miR390 which guides the cleavage of TAS3 precursor RNA. Seems to act as mi [...]	1.95	0.01	0.06
gi|241940764	AT3G25430 - Poly(A)-specific ribonuclease PARN-like protein; 3'-exoribonuclease that has	1.95	0.11	0.19

	a preference for poly(A) tails of mRNAs, thereby efficiently degrading poly(A) tails. Exonucleolytic degradation of the poly(A) tail is often the first step in the decay of eukaryotic mRNAs (By similarity)			
gi|241923619	TET8 - tetraspanin8; May be involved in the regulation of cell differentiation (By similarity)	1.94	0.03	0.09
gi|241931720	AT5G36228 - nucleic acid binding / zinc ion binding protein	1.92	0.24	0.28
gi|241926357	AT2G43460 - 60S ribosomal protein L38	1.91	0.09	0.17
gi|241946458	ACT7 - actin 7; Actins are highly conserved proteins that are involved in various types of cell motility and are ubiquitously expressed in all eukaryotic cells. Essential component of cell cytoskeleton; plays an important role in cytoplasmic streaming, cell shape determination, cell division, organelle movement and extension growth. This is considered as one of the vegetative actins which is involved in the regulation of hormone-induced plant cell proliferation and callus formation	1.91	0.00	0.02
gi|241924663	CYP93D1 - cytochrome P450, family 93, subfamily D, polypeptide 1	1.91	0.09	0.17
gi|241935376	AT4G32110 - beta-1,3-N-Acetylglucosaminyltransferase family protein	1.90	0.08	0.16
gi|241946976	OMT1 - O-methyltransferase 1; Methylates OH residues of flavonoid compounds. Converts quercetin into isorhamnetin. Dihydroquercetin is not a substrate. Catalyzes the methylation of monolignols, the lignin precursors. Does not contribute to the phenylpropanoid pattern of the	1.90	0.00	0.02

	pollen tryphine, but is probably confined to isorhamnetin gylcoside biosynthesis			
gi|241940843	AT2G03200 - aspartyl protease-like protein	1.89	0.05	0.12
gi|241944360	AATP1 - AAA-ATPase 1	1.87	0.02	0.07
gi|241938664	AT3G05950 - germin-like protein subfamily 1 member 7; May play a role in plant defense. Probably has no oxalate oxidase activity even if the active site is conserved	1.86	0.02	0.08
gi|241939008	AT1G01540 - putative serine/threonine-protein kinase	1.86	0.02	0.07
gi|241935381	AT3G48770 - ATP/DNA binding protein	1.85	0.04	0.11
gi|241917750	APS3 - 3'-phosphoadenosine 5'-phosphosulfate synthase	1.85	0.05	0.13
gi|241934212	FIM5 - FIMBRIN5; Cross-links actin filaments (F-actin). Stabilizes and prevents F-actin depolymerization mediated by profilin. May regulate actin cytoarchitecture, cell cycle, cell division, cell elongation and cytoplasmic tractus (By similarity)	1.85	0.06	0.15
gi|241930480	AT4G28300 - uncharacterized protein	1.84	0.01	0.06
gi|241926347	AT4G26830 - O-glycosyl hydrolase-17	1.84	0.06	0.15
gi|241930496	PPC3 - phosphoenolpyruvate carboxylase 3; Through the carboxylation of phosphoenolpyruvate (PEP) it forms oxaloacetate, a four-carbon dicarboxylic acid source for the tricarboxylic acid cycle	1.84	0.01	0.06
gi|241928446	AT2G21580 - 40S ribosomal protein S25-2	1.84	0.08	0.17
gi|241919296	GSTL2 - glutathione transferase lambda 2; Catalyzes the glutathione-dependent reduction of S- glutathionylquercetin to quercetin. In vitro, possesses glutathione-dependent thiol transferase activity toward 2- hydroxyethyl disulfide (HED)	1.83	0.09	0.18

gi|241930617	GPDHC1 - 6-phosphogluconate dehydrogenase-like protein; Involved in cell redox homeostasis. Required for maintaining a steady state cellular NADH/NAD(+) ratio through a mitochondrial glycerol-3-phosphate redox shuttle. May function with the mitochondrial FAD-dependent glycerol-3-phosphate dehydrogenase SDP6 to shuttle reducing equivalents into the mitochondria for respiration	1.83	0.01	0.07
gi|241937302	APX3 - L-ascorbate peroxidase; Plays a key role in hydrogen peroxide removal (By similarity)	1.82	0.01	0.04
gi|241939376	PAL1 - PHE ammonia lyase 1; This is a key enzyme of plant metabolism catalyzing the first reaction in the biosynthesis from L-phenylalanine of a wide variety of natural products based on the phenylpropane skeleton	1.82	0.05	0.13
gi|241936195	AT1G64010 - serine protease inhibitor-like protein	1.82	0.13	0.21
gi|241915743	AT3G28580 - AAA-type ATPase family protein	1.82	0.00	0.03
gi|241935200	APY1 - apyrase 1; Catalyzes the hydrolysis of phosphoanhydride bonds of nucleoside tri- and di-phosphates. Substrate preference is ATP > ADP. Functions with APY2 to reduce extracellular ATP level which is essential for pollen germination and normal plant development. Plays a role in the regulation of stomatal function by modulating extracellular ATP levels in guard cells	1.82	0.01	0.05
gi|241922062	GAD - glutamate decarboxylase; Catalyzes the production of GABA. The calmodulin-binding is calcium-dependent and it is proposed that this	1.82	0.00	0.03

	may, directly or indirectly, form a calcium regulated control of GABA biosynthesis			
gi|241927139	AT3G14880 - uncharacterized protein	1.82	0.03	0.10
gi|241925402	DL1E - DYNAMIN-like 1E; Microtubule-associated force-producing protein that is targeted to the tubulo-vesicular network of the forming cell plate during cytokinesis. Plays also a major role in plasma membrane maintenance and cell wall integrity with an implication in vesicular trafficking, polar cell expansion, and other aspects of plant growth and development	1.82	0.04	0.11
gi|241930041	HIR1 - HYPERSENSITIVE-INDUCED RESPONSE PROTEIN 1	1.81	0.02	0.09
gi|241931686	PPC3 - phosphoenolpyruvate carboxylase 3; Through the carboxylation of phosphoenolpyruvate (PEP) it forms oxaloacetate, a four-carbon dicarboxylic acid source for the tricarboxylic acid cycle	1.81	0.01	0.05
gi|241932635	EXPB4 - expansin B4; May cause loosening and extension of plant cell walls by disrupting non-covalent bonding between cellulose microfibrils and matrix glucans. No enzymatic activity has been found (By similarity)	1.81	0.02	0.08
gi|241941429	ELF7 - EARLY FLOWERING 7	1.80	0.14	0.21
gi|241940639	ABCA7 - ATP-binding cassette A7	1.79	0.02	0.08
gi|241915915	AT4G17720 - RNA recognition motif-containing protein	1.79	0.02	0.08
gi|219766905	DMR6 - DOWNY MILDEW RESISTANT 6	1.79	0.08	0.16
gi|241946875	AT4G28000 - AAA-type ATPase family protein	1.79	0.01	0.07
gi|241935756	VPS15 - vacuolar protein sorting 15	1.79	0.17	0.24
gi|241937673	AT4G25740 - 40S ribosomal protein S10-1	1.79	0.01	0.07
gi|241920167	AT1G53645 - hydroxyproline-rich glycoprotein-like protein	1.79	0.07	0.16

gi|241928905	GSTU8 - glutathione S-transferase TAU 8; May be involved in the conjugation of reduced glutathione to a wide number of exogenous and endogenous hydrophobic electrophiles and have a detoxification role against certain herbicides (By similarity)	1.78	0.08	0.16
gi|241945083	CXE17 - carboxyesterase 17; Carboxylesterase acting on esters with varying acyl chain length (By similarity)	1.78	0.03	0.09
gi|241943244	CHX19 - cation/H+ exchanger 19; May operate as a cation/H(+) antiporter (By similarity)	1.78	0.06	0.14
gi|241919513	At2g03440 - nodulin-related protein 1	1.78	0.02	0.08
gi|241935100	ChiC - class V chitinase	1.78	0.37	0.35
gi|241934564	CLPP6 - CLP protease proteolytic subunit 6; Cleaves peptides in various proteins in a process that requires ATP hydrolysis. Has a chymotrypsin-like activity. Plays a major role in the degradation of misfolded proteins (By similarity). Essential protein required for chloroplast development and integrity	1.78	0.05	0.13
gi|241943126	AT5G64130 - cAMP-regulated phosphoprotein 19-related protein	1.77	0.03	0.09
gi|241921860	PYL6 - PYR1-like 6; Receptor for abscisic acid (ABA) required for ABA- mediated responses such as stomatal closure and germination inhibition. Inhibits the activity of group-A protein phosphatases type 2C (PP2Cs) when activated by ABA (By similarity)	1.77	0.07	0.15
gi|241921038	UGT74F2 - UDP-glucosyltransferase 74F2; Glycosyltransferase that glucosylates benzoic acid and derivatives. Substrate preference is benzoic acid > salicylic acid (SA) > 3-hydroxybenzoic acid > 4-hydroxybenzoic acid.	1.77	0.01	0.06

	Catalyzes the formation of both SA 2-O-beta-D-glucoside (SAG) and SA glucose ester (SGE). Has high affinity for the tryptophan precursor anthranilate. Catalyzes the formation of anthranilate glucose ester. Is the major source of this activity in the plant			
gi|241931074	GSTU8 - glutathione S-transferase TAU 8; May be involved in the conjugation of reduced glutathione to a wide number of exogenous and endogenous hydrophobic electrophiles and have a detoxification role against certain herbicides (By similarity)	1.77	0.07	0.15
gi|241920333	PRA7 - PRA1 family protein F2; May be involved in both secretory and endocytic intracellular trafficking in the endosomal/prevacuolar compartments (By similarity)	1.77	0.01	0.06
gi|241944260	AT5G46160 - large subunit ribosomal protein L14	1.76	0.11	0.19
gi|241923561	VIT1 - vacuolar iron transporter 1; Vacuolar iron transporter involved in the transfer of iron from the cytosol to the vacuole for intracellular iron storage. Vacuolar iron storage is required for seed embryo and seedling development	1.76	0.11	0.19
gi|241917203	CID5 - CTC-interacting domain 5	1.76	0.02	0.08
gi|241918149	SC3 - secretory carrier 3; Probably involved in membrane trafficking (By similarity)	1.76	0.02	0.08
gi|241945927	AT3G17020 - universal stress protein (USP) family protein	1.75	0.02	0.09
gi|241930888	AT3G07570 - Cytochrome b561/ferric reductase transmembrane with DOMON related domain	1.75	0.06	0.14
gi|241921619	AT1G35780 - uncharacterized protein	1.75	0.03	0.09

gi|241916072	AT5G46030 - transcription elongation factor 1-like protein; Transcription elongation factor implicated in the maintenance of proper chromatin structure in actively transcribed regions (By similarity)	1.74	0.03	0.10
gi|241942343	AT2G14095 - uncharacterized protein	1.74	0.28	0.31
gi|241944468	AT5G48760 - 60S ribosomal protein L13a-4	1.74	0.13	0.21
gi|241925900	AT2G22420 - peroxidase; Removal of H(2)O(2), oxidation of toxic reductants, biosynthesis and degradation of lignin, suberization, auxin catabolism, response to environmental stresses such as wounding, pathogen attack and oxidative stress. These functions might be dependent on each isozyme/isoform in each plant tissue	1.74	0.00	0.04
gi|241943470	LP1 - lipid transfer protein 1; Plant non-specific lipid-transfer proteins transfer phospholipids as well as galactolipids across membranes. May play a role in wax or cutin deposition in the cell walls of expanding epidermal cells and certain secretory tissues	1.74	0.03	0.10
gi|21326116	AT5G48760 - 60S ribosomal protein L13a-4	1.74	0.04	0.12
gi|241915714	RD21B - esponsive to dehydration 21B	1.74	0.03	0.11
gi|241927204	CYP77A5P - cytochrome P450, family 77, subfamily A, polypeptide 5 pseudogene	1.74	0.01	0.07
gi|241938178	AT5G65960 - GTP binding protein	1.74	0.05	0.13
gi|241941959	F28P22.20 - germin-like protein 1; May play a role in plant defense. Probably has no oxalate oxidase activity even if the active site is conserved	1.73	0.06	0.14
gi|241919534	AT3G19650 - cyclin-related protein	1.73	0.03	0.10
gi|241922195	AT3G25290 - putative auxin-responsive protein	1.73	0.04	0.11
gi|241929054	AT4G32760 - ENTH/VHS/GAT family protein	1.73	0.19	0.25

gi|241916215	AT3G16780 - 60S ribosomal protein L19-2	1.72	0.10	0.18
gi|241918260	AT5G23760 - putative copper transport protein	1.72	0.05	0.13
gi|241944359	AATP1 - AAA-ATPase 1	1.72	0.02	0.07
gi|241917376	PAP15 - purple acid phosphatase 15; Acid phosphatase activity with p-nitrophenyl phosphate (pNPP), D-myoinositol 1-phosphate (Ins(1)P1), phytic acid and Myo- inositol hexakisphosphate. Low or no activity with Glc-6-P and ATP. Confers shoot growth stimulation, enhanced salt and osmotic stress tolerance, and ABA insensitivity. May modulate ascorbic acid (AsA) levels by controlling the input of myoinositol into this branch of AsA biosynthesis	1.71	0.03	0.10
gi|241931185	AT4G17260 - L-lactate dehydrogenase	1.71	0.04	0.12
gi|241931889	RD19 - cysteine proteinase RD19a	1.71	0.02	0.07
gi|241919908	AT5G43310 - COP1-interacting protein-like protein	1.71	0.19	0.25
gi|241926919	AT5G45920 - GDSL esterase/lipase	1.71	0.04	0.11
gi|241917869	AT1G70750 - uncharacterized protein	1.70	0.06	0.14
gi|241929173	PFK3 - phosphofructokinase 3	1.70	0.19	0.25
gi|241931077	GSTU8 - glutathione S-transferase TAU 8; May be involved in the conjugation of reduced glutathione to a wide number of exogenous and endogenous hydrophobic electrophiles and have a detoxification role against certain herbicides (By similarity)	1.70	0.00	0.03
gi|241929275	GLP5 - germin-like protein 5; May play a role in plant defense. Probably has no oxalate oxidase activity even if the active site is conserved	1.70	0.06	0.14
gi|241930334	ABCB11 - P-glycoprotein 11	1.69	0.02	0.09
gi|241934692	AT3G45630 - CCR4-NOT transcription complex subunit 4	1.69	0.05	0.12

gi|241938959	AT3G09970 - calcineurin-like metallo-phosphoesterase-like protein	1.69	0.07	0.15
gi|241941805	ABCA2 - ATP-binding cassette A2	1.69	0.01	0.06
gi|241917405	AT3G23390 - 60S ribosomal protein L36a	1.69	0.12	0.20
gi|241942311	AT1G03370 - C2 calcium/lipid-binding and GRAM domain containing protein	1.68	0.09	0.17
gi|241933157	AT3G16780 - 60S ribosomal protein L19-2	1.68	0.02	0.08
gi|219725304	SSI2 - suppressor of SA insensitive 2; Converts stearoyl-ACP to oleoyl-ACP by introduction of a cis double bond between carbons Delta(9) and Delta(10) of the acyl chain. Required for the activation of certain jasmonic acid (JA)-mediated responses and the repression of the salicylic acid (SA) signaling pathway	1.68	0.00	0.02
gi|241916338	AT5G39570 - uncharacterized protein	1.68	0.01	0.07
gi|241929241	AT1G17350 - putative complex I intermediate-associated protein 30; Chaperone protein involved in the assembly of the mitochondrial NADH:ubiquinone oxidoreductase complex (complex I) (By similarity)	1.68	0.05	0.13
gi|241945969	AT5G14540 - uncharacterized protein	1.68	0.09	0.17
gi|241936497	AT1G58170 - Disease resistance-responsive (dirigent-like protein) family protein	1.68	0.03	0.09
gi|241946722	AT1G80000 - CASC3/Barentsz eIF4AIII binding protein	1.67	0.05	0.13
gi|241931032	LBO1 - LATERAL BRANCHING OXIDOREDUCTASE 1	1.67	0.00	0.04
gi|241942564	VIP1 - VIRE2-interacting protein 1; Transcription activator that binds specifically to the VIP1 response elements (VREs) DNA sequence 5'-ACNGCT-3' found in some stress genes (e.g. TRX8 and MYB44), when phosphorylated/activated by MPK3. Required	1.67	0.03	0.10

	for Agrobacterium VirE2 nuclear import and tumorigenicity. Promotes transient expression of T-DNA in early stages by interacting with VirE2 in complex with the T-DNA and facilitating its translocation to the nucleus, and mediates stable genetic transformation by Agrobacterium by binding H2A histone. Prevents cell differentiation and shoot formation. Li [...]			
gi|241918888	GSTU18 - glutathione S-transferase TAU 18; May be involved in the conjugation of reduced glutathione to a wide number of exogenous and endogenous hydrophobic electrophiles and have a detoxification role against certain herbicides (By similarity)	1.67	0.03	0.10
gi|241946629	SC3 - secretory carrier 3; Probably involved in membrane trafficking (By similarity)	1.67	0.01	0.07
gi|241944027	AT2G47710 - adenine nucleotide alpha hydrolases-like protein	1.67	0.08	0.16
gi|58978057	PYL12 - PYR1-like 12; Receptor for abscisic acid (ABA) required for ABA- mediated responses such as stomatal closure and germination inhibition. Inhibits the activity of group-A protein phosphatases type 2C (PP2Cs) when activated by ABA (By similarity)	1.67	0.01	0.07
gi|241920677	SERPIN1 - serpin-ZX; Inhibits metacaspase-9 cysteine protease	1.67	0.02	0.08
gi|241924277	AT1G20110 - RING/FYVE/PHD zinc finger-containing protein	1.67	0.01	0.04
gi|241930323	GSTU18 - glutathione S-transferase TAU 18; May be involved in the conjugation of reduced glutathione to a wide number of exogenous and endogenous hydrophobic electrophiles and	1.67	0.03	0.10

	have a detoxification role against certain herbicides (By similarity)			
gi|194326198	GLN1-1 - glutamine synthase clone R1; High-affinity glutamine synthetase. May contribute to the homeostatic control of glutamine synthesis in roots	1.66	0.01	0.06
gi|241924066	AT1G60730 - NAD(P)-linked oxidoreductase-like protein	1.66	0.01	0.05
gi|241940229	NRT1.1 - nitrate transporter 1.1; Dual affinity nitrate transporter. Involved in proton-dependent nitrate uptake and in the regulation of the nitrate transporter NRT2.1. Acts also as a nitrate sensor that trigger a specific signaling pathway stimulating lateral root growth and seed germination. The uptake activity is not required for sensor function. Displays an auxin transport facilitation inhibited by high nitrate concentration. Required to prevent auxin accumulation in preemerged lateral root primordia and young lateral roots when external nitrate concentration is low or null. May be involv [...]	1.66	0.05	0.13
gi|669027005	AT5G47260 - putative disease resistance protein; Potential disease resistance protein (By similarity)	1.66	0.22	0.27
gi|241932190	AT1G61690 - phosphoinositide binding protein	1.65	0.11	0.19
gi|241921452	ERD9 - glutathione S-transferase; Involved in light signaling, mainly phyA-mediated photomorphogenesis and in the integration of various phytohormone signals to modulate various aspects of plant development by affecting glutathione pools. In vitro, possesses glutathione S- transferase activity toward 1-	1.65	0.02	0.09

	chloro-2,4-dinitrobenzene (CDNB) and benzyl isothiocyanate (BITC)			
gi|241935903	MT2A - metallothionein 2A; Metallothioneins have a high content of cysteine residues that bind various heavy metals	1.65	0.04	0.12
gi|241922547	EXPB2 - expansin B2; May cause loosening and extension of plant cell walls by disrupting non-covalent bonding between cellulose microfibrils and matrix glucans. No enzymatic activity has been found (By similarity)	1.65	0.03	0.09
gi|241926582	AT5G64220 - calmodulin-binding transcription activator 2; Regulates transcriptional activity in response to calcium signals. Binds to the consensus sequence 5'- [ACG]CGCG[GTC]-3'	1.64	0.04	0.11
gi|241921274	PTR2 - peptide transporter 2; Peptide transporter. Mediates the transport of di- and tripeptides. High affinity, low capacity transporter. Can also transport histidine	1.64	0.15	0.23
gi|241932892	ATI1 - uncharacterized protein	1.64	0.08	0.17
gi|241915166	EXT-like - extensin-like	1.64	0.01	0.04
gi|241933691	AT1G14130 - 2-oxoglutarate (2OG) and Fe(II)-dependent oxygenase-like protein	1.64	0.18	0.24
gi|241928760	BGLU42 - beta glucosidase 42	1.63	0.01	0.05
gi|241927967	CYP72A14 - cytochrome P450, family 72, subfamily A, polypeptide 14	1.63	0.04	0.12
gi|241934501	RPT1A - regulatory particle triple-A 1A; The 26S protease is involved in the ATP-dependent degradation of ubiquitinated proteins. The regulatory (or ATPase) complex confers ATP dependency and substrate specificity to the 26S complex	1.63	0.01	0.05
gi|241936083	AT3G10300 - putative calcium-binding protein CML49; Potential calcium sensor (By similarity)	1.63	0.03	0.11

gi|241920635	NSF - vesicle-fusing ATPase; Involved in vesicle-mediated transport. The ATPase activity of NSF serves to disassemble the SNARE complex, freeing the components for subsequent pairing and fusion events	1.63	0.00	0.04
gi|241919403	AT2G45600 - alpha/beta-hydrolase domain-containing protein; Carboxylesterase acting on esters with varying acyl chain length (By similarity)	1.63	0.02	0.08
gi|241926402	AT1G61260 - uncharacterized protein	1.63	0.03	0.11
gi|241926602	AT5G39850 - 40S ribosomal protein S9-2	1.62	0.01	0.07
gi|241919029	SCPL51 - serine carboxypeptidase-like 51; Probable carboxypeptidase (By similarity)	1.62	0.01	0.05
gi|241931538	BUBR1 - checkpoint serine/threonine-protein kinase	1.62	0.04	0.12
gi|241931076	GSTU8 - glutathione S-transferase TAU 8; May be involved in the conjugation of reduced glutathione to a wide number of exogenous and endogenous hydrophobic electrophiles and have a detoxification role against certain herbicides (By similarity)	1.62	0.01	0.06
gi|241929733	UXS4 - UDP-xylose synthase 4; Catalyzes the NAD-dependent decarboxylation of UDP-glucuronic acid to UDP-xylose. Necessary for the biosynthesis of the core tetrasaccharide in glycosaminoglycan biosynthesis (By similarity)	1.62	0.01	0.06
gi|241931125	AT4G25740 - 40S ribosomal protein S10-1	1.62	0.00	0.03
gi|241928916	GLP10 - germin-like protein 10; May play a role in plant defense. Probably has no oxalate oxidase activity even if the active site is conserved	1.61	0.11	0.19
gi|241942991	AT1G28600 - GDSL esterase/lipase	1.61	0.14	0.21

gi|241946839	DSEL - lipase class 3 family protein; Acylhydrolase that catalyzes the hydrolysis of 1,3- diacylglycerol (1,3-DAG) and 1-monoacylglycerol (1-MAG) at the sn- 1 position. High activity toward 1,3-DAG and 1-MAG, but low activity toward 1,2-diacylglycerol (1,2-DAG) and 1- lysophosphatidylcholine (1-LPC), and no activity toward phosphatidylcholine (PC), monogalactosyldiacylglycerol (MGDG), digalactosyldiacylglycerol (DGDG), triacylglycerol (TAG) and 2- monoacylglycerol (2-MAG). May be involved in the negative regulation of seedling establishment by inhibiting the breakdown, beta-oxidation and mobi [...]	1.61	0.03	0.09
gi|241917910	AT1G55265 - uncharacterized protein	1.61	0.04	0.12
gi|241940977	AT2G45600 - alpha/beta-hydrolase domain-containing protein; Carboxylesterase acting on esters with varying acyl chain length (By similarity)	1.61	0.00	0.03
gi|241944563	ATGSTF13 - Glutathione S-transferase-like protein; May be involved in the conjugation of reduced glutathione to a wide number of exogenous and endogenous hydrophobic electrophiles and have a detoxification role against certain herbicides (By similarity)	1.61	0.01	0.06
gi|241926905	GH9C3 - endoglucanase 19	1.60	0.04	0.11
gi|241928375	AT3G49800 - BSD domain-containing protein	1.60	0.00	0.04
gi|241918012	SULTR1;3 - sulfate transporter 1.3; High-affinity H(+)/sulfate cotransporter that mediates the loading of sulfate into the sieve tube. Plays a central role in the regulation of sulfate assimilation	1.60	0.10	0.18

gi|219783544	AT2G25520 - putative phosphate/phosphoenolpyruvate translocator protein	1.60	0.03	0.10
gi|241931258	CDC2 - cell division control 2; Involved in the control of the cell cycle. Essential for both G1/S and G2/M (mitosis) phase transitions. Functions in cell morphogenesis as well as cell proliferation. Required for cell division (entry into mitosis) of the generative cell in male gametogenesis	1.59	0.08	0.17
gi|241946439	AT1G22540 - putative peptide/nitrate transporter	1.59	0.02	0.08
gi|241920339	AT1G56290 - CwfJ-like protein	1.59	0.02	0.08
gi|241916873	SPX1 - SPX domain gene 1; Plays a positive role in plant adaptation to phosphate starvation	1.59	0.06	0.14
gi|241939986	NHL2 - NDR1/HIN1-like 2	1.59	0.05	0.13
gi|241925856	ANNAT7 - annexin 7	1.59	0.08	0.16
gi|241926293	APR3 - APS reductase 3; Reduces sulfate for Cys biosynthesis. Substrate preference is adenosine-5'-phosphosulfate (APS) >> 3'-phosphoadenosine-5'-phosphosulfate (PAPS). Uses glutathione or DTT as source of protons	1.59	0.03	0.10
gi|241934315	CBF2 - C-repeat/DRE binding factor 2; Transcriptional activator that binds specifically to the DNA sequence 5'-[AG]CCGAC-3'. Binding to the C-repeat/DRE element mediates cold-inducible transcription. CBF/DREB1 factors play a key role in freezing tolerance and cold acclimation	1.58	0.01	0.07
gi|241941872	HMGA - high mobility group A	1.58	0.03	0.09
gi|241937753	AT1G47500.1 - RNA-binding protein 47C'; Heterogeneous nuclear ribonucleoprotein (hnRNP)-protein binding the poly(A) tail of	1.58	0.03	0.10

	mRNA and probably involved in some steps of pre-mRNA maturation (By similarity)			
gi|241921629	XYP1 - xylogen protein 1	1.58	0.01	0.06
gi|241917233	AT5G14050 - U3 small nucleolar RNA-associated protein 18-like protein; Involved in nucleolar processing of pre-18S ribosomal RNA (By similarity)	1.58	0.05	0.12
gi|219891408	UGT84A1 - UDP-glycosyltransferase-like protein; Glucosyltransferase that glucosylates 4-coumarate, ferulate, caffeate, sinapate and cinnamate. Can glucosylate the phytotoxic xenobiotic compound 2,4,5-trichlorophenol (TCP)	1.58	0.00	0.04
gi|241945077	CYSB - cystatin B; Specific inhibitor of cysteine proteinases. Probably involved in the regulation of endogenous processes and in defense against pests and pathogens (By similarity)	1.58	0.01	0.07
gi|241929023	ADCL - 4-amino-4-deoxychorismate lyase	1.57	0.02	0.07
gi|241927140	NFXL2 - NFX1-like 2; Probable transcriptional regulator. May mediate E2- or E3-dependent ubiquitination. Required to gate light sensitivity during the night. Regulates the speed of the clock by acting in the feedback loop between CCA1, LHY and APRR1/TOC1. Promotes the expression of CCA1 at night but not by days. This activational effect is enhanced by interaction with ADO1/ZTL. Association with ADO1/ZTL is not leading to the degradation of NFXL2. Confers sensitivity to osmotic stress such as high salinity. Prevents H(2)O(2) production and abscisic acid accumulation. Part of a regulatory networ [...]	1.57	0.05	0.13

gi|241946033	AT4G25150 - HAD superfamily, subfamily IIIB acid phosphatase	1.57	0.04	0.12
gi|241919109	BIGYIN - protein BIGYIN1	1.57	0.02	0.09
gi|241925303	EXPB4 - expansin B4; May cause loosening and extension of plant cell walls by disrupting non-covalent bonding between cellulose microfibrils and matrix glucans. No enzymatic activity has been found (By similarity)	1.57	0.02	0.08
gi|241922716	RPM1 - disease resistance protein RPM1; Disease resistance (R) protein that specifically recognizes the AvrRpm1 type III effector avirulence protein from Pseudomonas syringae. Resistance proteins guard the plant against pathogens that contain an appropriate avirulence protein via an indirect interaction with this avirulence protein. That triggers a defense system including the hypersensitive response, which restricts the pathogen growth. Acts via its interaction with RIN4, and probably triggers the plant resistance when RIN4 is phosphorylated by AvrRpm1. It is then degraded at the onset of th [...]	1.57	0.02	0.08
gi|241923486	HIR1 - HYPERSENSITIVE-INDUCED RESPONSE PROTEIN 1	1.56	0.01	0.06
gi|241940359	AT1G56130 - putative LRR receptor-like serine/threonine-protein kinase	1.56	0.04	0.11
gi|241917392	AT1G27400 - 60S ribosomal protein L17-1	1.56	0.12	0.20
gi|241944367	SOS1 - SALT OVERLY SENSITIVE 1; Acts in electroneutral exchange of protons for cations such as Na(+) or Li(+) across plasma membrane. Involved in Na(+) and K(+) homeostasis. Required for cytoplasmic Na(+) and Li(+) detoxification by secreting them from	1.55	0.01	0.07

	the cytoplasm to the extracellular space. Regulates Na(+) content of the xylem sap			
gi|241927268	AT3G09410 - putative pectinacetylesterase	1.55	0.02	0.09
gi|241917554	AT4G38810 - EF-hand, calcium binding motif-containing protein	1.55	0.01	0.07
gi|241928417	AT2G41790 - insulysin; Peptidase that might be involved in pathogen or wound response. Not required for peroxisome biogenesis, indole-3-butyric acid (IBA) metabolism, fatty acid beta-oxidation or degradation of glyoxylate cycle enzymes during seedling development	1.55	0.01	0.05
gi|241945412	AT4G17520 - plasminogen activator inhibitor 1 RNA-binding protein	1.55	0.02	0.08
gi|241937255	AT2G45630 - D-isomer specific 2-hydroxyacid dehydrogenase-like protein	1.55	0.01	0.04
gi|18390108	AT3G13990 - uncharacterized protein	1.55	0.02	0.07
gi|241936032	AT2G09990 - 40S ribosomal protein S16-1	1.55	0.03	0.10
gi|241942172	UKL2 - uridine kinase-like 2; Involved in the pyrimidine salvage pathway. The uracil phosphoribosyltransferase (UPRT) activity, that catalyzes the conversion of uracil and 5-phospho-alpha-D-ribose 1-diphosphate (PRPP) to UMP and diphosphate, is unsure	1.55	0.04	0.12
gi|241921951	HSP70 - heat shock protein 70; Component of the Mediator complex, a coactivator involved in the regulated transcription of nearly all RNA polymerase II-dependent genes. Mediator functions as a bridge to convey information from gene-specific regulatory proteins to the basal RNA polymerase II transcription machinery. The Mediator complex, having a compact conformation in its free form, is recruited to promoters by direct interactions	1.55	0.01	0.06

	with regulatory proteins and serves for the assembly of a functional preinitiation complex with RNA polymerase II and the general transcription factors (By similarity)			
gi|241934559	THA1 - threonine aldolase	1.55	0.07	0.16
gi|241928058	CHIA - chitinase A	1.55	0.02	0.09
gi|241937382	KEU - keule; Regulator of vesicle trafficking involved in cytokinesis and root hair development, but not required for cell elongation	1.54	0.00	0.04
gi|241940008	AT1G71695 - peroxidase 12; Removal of H(2)O(2), oxidation of toxic reductants, biosynthesis and degradation of lignin, suberization, auxin catabolism, response to environmental stresses such as wounding, pathogen attack and oxidative stress. These functions might be dependent on each isozyme/isoform in each plant tissue	1.54	0.01	0.07
gi|241934088	AT5G64160 - uncharacterized protein	1.54	0.03	0.11
gi|241940921	AT5G23950 - calcium-dependent lipid-binding domain-containing protein	1.54	0.02	0.08
gi|241924929	CHIA - chitinase A	1.54	0.03	0.11
gi|241925991	AT3G25290 - putative auxin-responsive protein	1.54	0.05	0.12
gi|241932855	PP2-B1 - phloem protein 2-B1; Component of SCF(ASK-cullin-F-box) E3 ubiquitin ligase complexes, which may mediate the ubiquitination and subsequent proteasomal degradation of target proteins (By similarity)	1.53	0.01	0.07
gi|241938626	AT4G10540 - Subtilase family protein	1.53	0.01	0.07
gi|241934156	AT5G17190 - uncharacterized protein	1.53	0.03	0.11
gi|241935198	CHIA - chitinase A	1.53	0.01	0.06
gi|241932736	ERECTA - ERECTA; Receptor kinase that, together with ERL1 and ERL2, regulates aerial	1.53	0.01	0.07

	architecture, including inflorescence (e.g. shoot apical meristem-originating organ shape, elongation of the internode and pedicels, and adaxial-abaxial polarity), and stomatal patterning (e.g. density and clustering), probably by tuning cell division and expansion. Modulates plant transpiration efficiency by controlling stomatal density, leaf photosynthetic capacity, epidermal cell expansion, mesophyll cell proliferation and cell-cell contact. Probable major trait regulating canalization (maintenance of phenot [...]			
gi|241943504	ACR12 - ACT domain repeats 12	1.53	0.00	0.03
gi|241915415	AT4G33110 - S-adenosyl-L-methionine-dependent methyltransferase-like protein	1.53	0.03	0.10
gi|241946305	BIGYIN - protein BIGYIN1	1.53	0.04	0.12
gi|241916164	RPL23AA - ribosomal protein L23AA; Binds to a specific region on the 26S rRNA (By similarity)	1.53	0.08	0.16
gi|241917702	AT4G34290 - SWIB/MDM2 domain-containing protein	1.53	0.04	0.12
gi|241920188	GRIK1 - geminivirus rep interacting kinase 1; Activates SnRK1.1/KIN10 and SnRK1.2/KIN11 by phosphorylation of their activation-loop 'Thr-198' and 'Thr-176', respectively. Required for the regulation by SnRK1 kinases of the transcription of a large set of genes, the modification the activity of metabolic enzymes, and the control of various nutrient-responsive cellular developmental processes	1.52	0.05	0.13
gi|241914900	HON4 - DNA-binding protein HMR1-like protein	1.52	0.03	0.11
gi|241926606	AT5G39850 - 40S ribosomal protein S9-2	1.52	0.01	0.04
gi|219898271	RPS13A - ribosomal protein S13A	1.52	0.02	0.09
gi|241924078	PAP27 - purple acid phosphatase 27	1.51	0.00	0.03

gi|241933953	VPS20.2 - vacuolar protein sorting-associated protein 20-2; Component of the ESCRT-III complex, which is required for multivesicular bodies (MVBs) formation and sorting of endosomal cargo proteins into MVBs. The ESCRT-III complex is probably involved in the concentration of MVB cargo (By similarity)	1.51	0.01	0.06
gi|241929350	AT1G19240 - uncharacterized protein	1.51	0.02	0.08
gi|241937149	ABCG11 - ATP-binding cassette G11; Required for the cuticle and pollen coat development by controlling cutin and maybe wax transport to the extracellular matrix. Involved in developmental plasticity and stress responses	1.51	0.01	0.06
gi|241938829	scpl20 - carboxypeptidase C; Probable carboxypeptidase (By similarity)	1.51	0.02	0.07
gi|241942714	ATGSTF13 - Glutathione S-transferase-like protein; May be involved in the conjugation of reduced glutathione to a wide number of exogenous and endogenous hydrophobic electrophiles and have a detoxification role against certain herbicides (By similarity)	1.51	0.01	0.05
gi|241917595	AT3G09630 - 60S ribosomal protein L4-1	1.51	0.02	0.08
gi|241940976	AT5G61170 - 40S ribosomal protein S19-3	1.51	0.05	0.13
gi|241939289	EP3 - chitinase	1.50	0.02	0.07
gi|241920138	AT2G30620 - histone H1.2; Histones H1 are necessary for the condensation of nucleosome chains into higher-order structures	0.67	0.01	0.04
gi|241935866	ACLA-3 - ATP-citrate lyase A-3; ATP citrate-lyase is the primary enzyme responsible for the synthesis of cytosolic acetyl-CoA, used for the elongation of fatty acids and biosynthesis of isoprenoids, flavonoids and malonated derivatives. May supply substrate to the	0.67	0.00	0.01

	cytosolic acetyl-CoA carboxylase, which generates the malonyl-CoA used for the synthesis of a multitude of compounds, including very long chain fatty acids and flavonoids. Required for normal growth and development and elongation of C18 fatty acids to C20 to C24 fatty acids in seeds. In contrast to all known animal ACL enzymes having [...]			
gi|241939702	GlcNA.1UT1 - N-acetylglucosamine-1-phosphate uridylyltransferase 1; Uridylyltransferase involved in the biosynthesis of UDP- glucosamine, an essential precursor for glycoprotein and glycolipid synthesis. Can use both UDP-glucosamine and the 4- epimer UDP-galactosamine as substrates, but no other sugars or NTPs	0.66	0.00	0.01
gi|241938417	ENDO2 - endonuclease 2; Probable endonuclease (By similarity). Can not hydrolyze single stranded DNA and does not cleave mismatches	0.66	0.00	0.02
gi|241917988	AT5G20970 - heat shock family protein	0.66	0.02	0.08
gi|241942126	AT3G59350 - protein kinase family protein	0.66	0.00	0.02
gi|241938206	MLP423 - MLP-like protein 423	0.66	0.00	0.03
gi|2766448	CYP98A3 - cytochrome P450 98A3; Cytochrome P450 which catalyzes 3'-hydroxylation of p- coumaric esters of shikimic/quinic acids to form lignin monomers. Can use p-coumarate, p-coumaraldehyde, p-coumaroyl methyl ester, 5-O-(4-coumaroyl) D-quinate and 5-O-(4-coumaroyl) shikimate as substrates, but not p-coumaryl alcohol, p-coumaroyl CoA, 1-O-p- coumaroyl-beta-D-glucose, p-hydroxy-cinnamyl alcohol,	0.66	0.00	0.01

	cinnamate, caffeate or ferulate. Has a weak activity on tri(p- coumaroyl)spermidine, but none on triferuloylspermidine. Hydroxylates preferentially the 5-O-isomer, but can also convert the 4-O- and 3-O-i [...]			
gi|34541998	AT3G14470 - putative disease resistance RPP13-like protein 1; Potential disease resistance protein	0.66	0.02	0.07
gi|398559791	AT3G15670 - late embryogenesis abundant domain-containing protein	0.66	0.00	0.04
gi|241920539	RXF12 - glycosyl hydrolase-like prottein 10	0.65	0.00	0.03
gi|241927471	AT4G33420 - peroxidase; Removal of H(2)O(2), oxidation of toxic reductants, biosynthesis and degradation of lignin, suberization, auxin catabolism, response to environmental stresses such as wounding, pathogen attack and oxidative stress. These functions might be dependent on each isozyme/isoform in each plant tissue	0.65	0.01	0.05
gi|241920686	ARA12 - subtilisin-like protease; Serine protease. Has a substrate preference for the hydrophobic residues Phe and Ala and the basic residue Asp in the P1 position, and for Asp, Leu or Ala in the P1' position	0.64	0.00	0.03
gi|241945305	RCI3 - peroxidase 3; Removal of H(2)O(2), oxidation of toxic reductants, biosynthesis and degradation of lignin, suberization, auxin catabolism, response to environmental stresses such as wounding, pathogen attack and oxidative stress. These functions might be dependent on each isozyme/isoform in each plant tissue	0.64	0.00	0.02
gi|241919390	BGAL8 - beta-galactosidase 8	0.64	0.00	0.03

gi|241923857	AIR12 - auxin-responsive-like protein	0.64	0.00	0.04
gi|241945532	FLA11 - FASCICLIN-like arabinogalactan-protein 11; May be a cell surface adhesion protein	0.64	0.00	0.04
gi|241930710	HTB9 - histone H2B; Core component of nucleosome. Nucleosomes wrap and compact DNA into chromatin, limiting DNA accessibility to the cellular machineries which require DNA as a template. Histones thereby play a central role in transcription regulation, DNA repair, DNA replication and chromosomal stability. DNA accessibility is regulated via a complex set of post-translational modifications of histones, also called histone code, and nucleosome remodeling (By similarity)	0.64	0.01	0.06
gi|241928695	AT4G09160 - patellin-5; Carrier protein that may be involved in membrane- trafficking events associated with cell plate formation during cytokinesis. Binds to some hydrophobic molecules such as phosphoinositides and promotes their transfer between the different cellular sites (By similarity)	0.63	0.00	0.02
gi|241945302	AT1G54290 - protein translation factor SUI1-2; Probably involved in translation	0.63	0.01	0.05
gi|241923085	AAO2 - aldehyde oxidase 2; In higher plant aldehyde oxidases (AO) appear to be homo- and heterodimeric assemblies of AO subunits with probably different physiological functions. In vitro, AO-gamma uses heptaldehyde, benzaldehyde, naphthaldehyde and cinnamaldehyde as substrates; AO-beta uses indole-3-acetaldehyde (IAAld), indole-3-	0.63	0.00	0.02

	aldehyde (IAld) and naphtaldehyde; the AAO2-AAO3 dimer uses abscisic aldehyde			
gi|241939219	AT2G42490 - primary-amine oxidase	0.63	0.00	0.02
gi|241927409	AT2G39040 - peroxidase 24; Removal of H(2)O(2), oxidation of toxic reductants, biosynthesis and degradation of lignin, suberization, auxin catabolism, response to environmental stresses such as wounding, pathogen attack and oxidative stress. These functions might be dependent on each isozyme/isoform in each plant tissue	0.63	0.00	0.02
gi|241930598	PDIL5-3 - PDI-like 5-3; Acts as a protein-folding catalyst that interacts with nascent polypeptides to catalyze the formation, isomerization, and reduction or oxidation of disulfide bonds (By similarity)	0.63	0.00	0.02
gi|241946020	HXK1 - hexokinase 1; Fructose and glucose phosphorylating enzyme. May be involved in the phosphorylation of glucose during the export from mitochondrion to cytosol. Acts as sugar sensor which may regulate sugar-dependent gene repression or activation. Mediates the effects of sugar on plant growth and development independently of its catalytic activity or the sugar metabolism. May regulate the execution of program cell death in plant cells	0.63	0.00	0.03
gi|241941370	AT5G54580 - RNA recognition motif-containing protein	0.63	0.01	0.04
gi|21326129	UGD2 - UDP-glucose dehydrogenase 2; Involved in the biosynthesis of UDP-glucuronic acid (UDP-GlcA), providing nucleotide sugars for cell-wall polymers. Required for the	0.63	0.00	0.02

	formation of cell wall ingrowths on the outer cell walls of nematode-induced syncytia			
gi|241929284	IMPA-9 - importin alpha isoform 9; Binds specifically and directly to substrates containing either a simple or bipartite NLS motif. Promotes docking of import substrates to the nuclear envelope (By similarity)	0.63	0.02	0.08
gi|241943398	ASD1 - alpha-L-arabinofuranosidase 1; May be involved in the coordinated dissolution of the cell wall matrix during abscission and in the secondary cell wall formation in xylem vessels. Prefers arabinoxylan, but may also use pectic arabinans as substrates	0.62	0.00	0.01
gi|241932822	GUS3 - glucuronidase 3; Endoglycosidase which is a cell surface and extracellular matrix-degrading enzyme. Cleaves heparan sulfate proteoglycans (HSPGs) into heparan sulfate side chains and core proteoglycans (By similarity)	0.62	0.00	0.03
gi|241935468	XTH5 - xyloglucan:xyloglucosyl transferase; Catalyzes xyloglucan endohydrolysis (XEH) and/or endotransglycosylation (XET). Cleaves and religates xyloglucan polymers, an essential constituent of the primary cell wall, and thereby participates in cell wall construction of growing tissues (By similarity)	0.61	0.00	0.02
gi|241919157	SUS3 - sucrose synthase 3; Sucrose-cleaving enzyme that provides UDP-glucose and fructose for various metabolic pathways. Modulates metabolic homeostasis and direct carbon towards starch synthesis in developing seeds	0.61	0.01	0.07
gi|241927216	RXF12 - glycosyl hydrolase-like prottein 10	0.61	0.00	0.04

gi|241938524	AT1G63220 - calcium-dependent lipid-binding domain-containing protein	0.61	0.00	0.01
gi|241922409	AT4G22380 - Ribosomal protein L7Ae/L30e/S12e/Gadd45 family protein	0.61	0.01	0.05
gi|241931677	PRX52 - peroxidase 52; Removal of H(2)O(2), oxidation of toxic reductants, biosynthesis and degradation of lignin, suberization, auxin catabolism, response to environmental stresses such as wounding, pathogen attack and oxidative stress. These functions might be dependent on each isozyme/isoform in each plant tissue	0.60	0.00	0.02
gi|241936880	NPC4 - phospholipase C	0.60	0.00	0.02
gi|241932482	AT1G76550 - pyrophosphate--fructose-6-phosphate 1-phosphotransferase; Regulatory subunit of pyrophosphate--fructose 6-phosphate 1-phosphotransferase (PFP) (By similarity)	0.60	0.00	0.01
gi|241921631	AT4G35160 - O-methyltransferase family 2 protein	0.60	0.00	0.04
gi|241919015	SUS4 - sucrose synthase 4; Sucrose-cleaving enzyme that provides UDP-glucose and fructose for various metabolic pathways (By similarity)	0.60	0.00	0.02
gi|241942847	AT5G18910 - protein kinase family protein	0.60	0.01	0.04
gi|241945829	AT5G66390 - peroxidase 72; Removal of H(2)O(2), oxidation of toxic reductants, biosynthesis and degradation of lignin, suberization, auxin catabolism, response to environmental stresses such as wounding, pathogen attack and oxidative stress. These functions might be dependent on each isozyme/isoform in each plant tissue	0.60	0.01	0.05

gi|241916757	PRX52 - peroxidase 52; Removal of H(2)O(2), oxidation of toxic reductants, biosynthesis and degradation of lignin, suberization, auxin catabolism, response to environmental stresses such as wounding, pathogen attack and oxidative stress. These functions might be dependent on each isozyme/isoform in each plant tissue	0.59	0.00	0.02
gi|241945533	FLA6 - FASCICLIN-like arabinogalactan 6; May be a cell surface adhesion protein	0.59	0.01	0.06
gi|241933084	AT5G20950 - Glycosyl hydrolase family protein	0.59	0.00	0.02
gi|241941527	NUC-L1 - nucleolin; Involved in pre-rRNA processing and ribosome assembly. Is associated with intranucleolar chromatin and pre-ribosomal particles and plays a role in controlling activation and repression of a specific subset of rRNA genes located in distinctive nucleolar organizer regions. Binds specifically rDNA chromatin and may be required to maintain rDNA chromatin structure, but is probably not required for the overall histone methylation status of 45S rRNA genes	0.59	0.01	0.04
gi|241919822	RCI3 - peroxidase 3; Removal of H(2)O(2), oxidation of toxic reductants, biosynthesis and degradation of lignin, suberization, auxin catabolism, response to environmental stresses such as wounding, pathogen attack and oxidative stress. These functions might be dependent on each isozyme/isoform in each plant tissue	0.59	0.00	0.03
gi|241922599	ENODL20 - early nodulin-like protein 20	0.58	0.00	0.01
gi|241928935	AT1G78830 - curculin-like (mannose-binding) lectin-like protein	0.58	0.00	0.03

gi|241922542	TUB2 - tubulin beta chain 2; Tubulin is the major constituent of microtubules. It binds two moles of GTP, one at an exchangeable site on the beta chain and one at a non-exchangeable site on the alpha chain	0.58	0.01	0.04
gi|241932186	AT1G43760 - DNAse I-like superfamily protein	0.58	0.01	0.07
gi|241924484	AT1G28600 - GDSL esterase/lipase	0.58	0.00	0.02
gi|241924477	AT4G33420 - peroxidase; Removal of H(2)O(2), oxidation of toxic reductants, biosynthesis and degradation of lignin, suberization, auxin catabolism, response to environmental stresses such as wounding, pathogen attack and oxidative stress. These functions might be dependent on each isozyme/isoform in each plant tissue	0.58	0.00	0.03
gi|241941549	PAO1 - Polyamine oxidase 1; Flavoenzyme that catalyzes the oxidation of the secondary amino group of spermine, norspermine and N(1)-acetylspermine. Substrate preference is norspermine > spermine > N(1)-acetylspermine. No activity detected when putrescine, spermidine or N(1)-acetylspermidine are used as substrates. Plays an important role in the regulation of polyamine intracellular concentration	0.57	0.00	0.01
gi|241940766	ALDH11A3 - aldehyde dehydrogenase 11A3; Important as a means of generating NADPH for biosynthetic reactions	0.57	0.01	0.05
gi|241939966	AT4G27450 - aluminum induced protein with YGL and LRDR motifs	0.57	0.00	0.02
gi|241944550	AT2G35840 - putative sucrose-phosphatase 2; Catalyzes the final step of sucrose synthesis (By similarity)	0.56	0.01	0.04

gi|241937799	CASP5 - UPF0497 membrane protein; Regulates membrane-cell wall junctions and localized cell wall deposition. Required for establishment of the Casparian strip membrane domain (CSD) and the subsequent formation of Casparian strips, a cell wall modification of the root endodermis that determines an apoplastic barrier between the intraorganismal apoplasm and the extraorganismal apoplasm and prevents lateral diffusion	0.56	0.01	0.06
gi|241941460	ACBP6 - acyl-CoA-binding protein 6; Binds medium- and long-chain acyl-CoA esters with very high affinity. May function as an intracellular carrier of acyl- CoA esters. Confers resistance to cold and freezing. Interacts with phosphatidylcholine and derivatives, but not phosphatidic acid and lysophosphatidylcholine. May be involved in phospholipid metabolism	0.56	0.01	0.06
gi|8979720	CBSX3 - CBS domain-containing protein	0.55	0.00	0.02
gi|241946183	ERD15 - dehydration-induced protein ERD15; Central component of stress responses that interacts with poly(A)-binding proteins. Negative regulator of abscisic acid (ABA) responses, including resistance to drought and freezing as well as stomatal closure regulation. Mediates resistance to the bacterial necrotroph pathogen Erwinia carotovora subsp. carotovora and promotes the induction of marker genes for systemic acquired resistance (SAR)	0.55	0.01	0.05
gi|241924168	ABCG39 - ATP-binding cassette G39; May be a general defense protein (By similarity)	0.55	0.00	0.04
gi|241928281	AT1G16740 - Ribosomal protein L20; Binds directly to 23S ribosomal RNA and is necessary	0.54	0.01	0.07

	for the in vitro assembly process of the 50S ribosomal subunit. It is not involved in the protein synthesizing functions of that subunit (By similarity)			
gi|241921108	AT5G40020 - pathogenesis-related thaumatin-like protein	0.54	0.01	0.06
gi|241932799	MAPR2 - putative steroid-binding protein 3	0.54	0.00	0.03
gi|241922581	AT3G01190 - peroxidase 27; Removal of H(2)O(2), oxidation of toxic reductants, biosynthesis and degradation of lignin, suberization, auxin catabolism, response to environmental stresses such as wounding, pathogen attack and oxidative stress. These functions might be dependent on each isozyme/isoform in each plant tissue	0.54	0.01	0.05
gi|241921304	AT1G27480 - Lecithin-cholesterol acyltransferase-like 1	0.53	0.00	0.02
gi|241920002	TRM7 - TON1 Recruiting Motif 7	0.53	0.01	0.04
gi|241946418	AT5G06730 - peroxidase 54; Removal of H(2)O(2), oxidation of toxic reductants, biosynthesis and degradation of lignin, suberization, auxin catabolism, response to environmental stresses such as wounding, pathogen attack and oxidative stress. These functions might be dependent on each isozyme/isoform in each plant tissue	0.53	0.00	0.02
gi|241933634	AT1G48090 - calcium-dependent lipid-binding-like protein	0.52	0.00	0.03
gi|241923081	AAO1 - aldehyde oxidase 1; In higher plants aldehyde oxidases (AO) appear to be homo- and heterodimeric assemblies of AO subunits with probably different physiological functions. AO-alpha may be involved in the biosynthesis	0.51	0.00	0.01

	of auxin, and in biosynthesis of abscisic acid (ABA) in seeds. In vitro, AO-alpha uses heptaldehyde, protocatechualdehyde, benzaldehyde, indole-3-aldehyde (IAld), indole-3-acetaldehyde (IAAld), cinnamaldehyde and citral as substrates; AO-beta uses IAAld, IAld and naphtaldehyde as substrates			
gi|241944523	AT3G62160 - HXXXD-type acyl-transferase-like protein	0.51	0.00	0.02
gi|241926930	CYP710A1 - cytochrome P450, family 710, subfamily A; Required to form the C-22 double bond in the sterol side chain. Possesses in vitro C-22 desaturase activity toward beta- sitosterol and produces stigmasterol	0.50	0.00	0.03
gi|241916753	PRX52 - peroxidase 52; Removal of H(2)O(2), oxidation of toxic reductants, biosynthesis and degradation of lignin, suberization, auxin catabolism, response to environmental stresses such as wounding, pathogen attack and oxidative stress. These functions might be dependent on each isozyme/isoform in each plant tissue	0.50	0.00	0.02
gi|241925850	MAP65-7 - microtubule-associated protein 65-7	0.50	0.01	0.05
gi|241918930	PPDK - pyruvate, phosphate dikinase 1; Formation of phosphoenolpyruvate. May be involved in regulating the flux of carbon into starch and fatty acids of seeds and in the remobilization of nitrogen reserves in senescing leaves	0.50	0.06	0.14
gi|241918822	AT5G46900 - bifunctional inhibitor/lipid-transfer protein/seed storage 2S albumin-like protein	0.50	0.00	0.02
gi|241944413	XCP1 - xylem cysteine peptidase 1; Probable thiol protease (By similarity)	0.50	0.00	0.02

gi|241925490	FLA2 - FASCICLIN-like arabinogalactan 2; May be a cell surface adhesion protein	0.49	0.00	0.04
gi|241943944	AT4G10490 - oxidoreductase, 2OG-Fe(II) oxygenase family protein	0.49	0.01	0.04
gi|241930931	HDA3 - histone deacetylase 3; Probably mediates the deacetylation of lysine residues on the N-terminal part of the core histones (H2A, H2B, H3 and H4). Histone deacetylation gives a tag for epigenetic repression and plays an important role in transcriptional regulation, cell cycle progression and developmental events. Required for histone H3 'Lys-9' deacetylation. Involved in rRNA gene silencing in nucleolar dominance. Seems to be implicated in the regulation of genes involved in seeds development	0.49	0.00	0.01
gi|241933499	AT5G45910 - GDSL esterase/lipase	0.49	0.00	0.04
gi|241924273	RPS13A - ribosomal protein S13A	0.48	0.01	0.06
gi|241922056	AT1G59960 - putative Aldo/keto reductase	0.48	0.00	0.01
gi|241925966	CRL1 - CCR(Cinnamoyl coA:NADP oxidoreductase)-like 1	0.47	0.00	0.02
gi|241918821	AT2G45180 - protease inhibitor/seed storage/lipid transfer protein (LTP) family protein	0.47	0.00	0.01
gi|241929811	CHR11 - chromatin-remodeling protein 11; Possesses intrinsic ATP-dependent nucleosome-remodeling activity. Constitutes the catalytic subunit of several complexes capable of forming ordered nucleosome arrays on chromatin in vitro (By similarity)	0.46	0.01	0.04
gi|1658193	CYP51G1 - CYTOCHROME P450 51G1; Involved in sterol biosynthesis. Catalyzes the 14-alpha demethylation of obtusifoliol to 4	0.46	0.00	0.02

	alpha-methyl-5 alpha-ergosta- 8,14,24(28)-trien-3 beta-ol			
gi|241945902	IDN2 - INVOLVED IN DE NOVO 2	0.45	0.02	0.09
gi|1127575	BGLU13 - beta glucosidase 13	0.45	0.00	0.01
gi|241920962	NUC-L2 - nucleolin; Involved in pre-rRNA processing and ribosome assembly (By similarity)	0.45	0.00	0.02
gi|241929684	AT4G33420 - peroxidase; Removal of H(2)O(2), oxidation of toxic reductants, biosynthesis and degradation of lignin, suberization, auxin catabolism, response to environmental stresses such as wounding, pathogen attack and oxidative stress. These functions might be dependent on each isozyme/isoform in each plant tissue	0.44	0.00	0.02
gi|241928307	PRF5 - profilin 5; Binds to actin and affects the structure of the cytoskeleton. At high concentrations, profilin prevents the polymerization of actin, whereas it enhances it at low concentrations. By binding to PIP2, it inhibits the formation of IP3 and DG (By similarity)	0.44	0.00	0.01
gi|241938260	AT3G23840 - HXXXD-type acyl-transferase-like protein	0.44	0.00	0.02
gi|241938096	AT4G12510 - bifunctional inhibitor/lipid-transfer protein/seed storage 2S albumin-like protein	0.43	0.00	0.03
gi|241933544	PCAP1 - plasma-membrane associated cation-binding protein 1; May be involved in intracellular signaling through interaction with PtdInsPs and calmodulin (CaM); may keep PtdInsPs attached to the plasma membrane until Ca(2+)-CaM reaches a competitive concentration subsequent to an increase	0.43	0.00	0.01

	triggered by a stimulus, thus leading to PtdInsPs release and subsequent activation of InsPs-dependent signaling cascade. Interacts competitively at the N-terminus with calcium ions and CaM (in a calcium-dependent manner), and with the phosphatidylinositol phosphates PtdIns(3,4,5)P(3), PtdIns(3,4)P(2), PtdIns([...]			
gi|241916084	AT5G45910 - GDSL esterase/lipase	0.43	0.00	0.03
gi|241921372	AT4G12510 - bifunctional inhibitor/lipid-transfer protein/seed storage 2S albumin-like protein	0.43	0.00	0.02
gi|241918803	EXPB2 - expansin B2; May cause loosening and extension of plant cell walls by disrupting non-covalent bonding between cellulose microfibrils and matrix glucans. No enzymatic activity has been found (By similarity)	0.42	0.00	0.02
gi|241944083	AT2G28790 - pathogenesis-related thaumatin-like protein	0.42	0.00	0.02
gi|241916764	AT4G20820 - FAD-binding and BBE domain-containing protein	0.41	0.00	0.03
gi|241945226	AT5G01320 - pyruvate decarboxylase	0.40	0.00	0.04
gi|241917101	AT4G35160 - O-methyltransferase family 2 protein	0.40	0.00	0.03
gi|241933765	AT2G27730 - copper ion binding protein	0.38	0.00	0.03
gi|241938097	AT4G12510 - bifunctional inhibitor/lipid-transfer protein/seed storage 2S albumin-like protein	0.34	0.00	0.04
gi|241930262	FLA11 - FASCICLIN-like arabinogalactan-protein 11; May be a cell surface adhesion protein	0.34	0.00	0.03
gi|241942902	AT4G10500 - oxidoreductase, 2OG-Fe(II) oxygenase family protein	0.30	0.00	0.03
gi|241922750	AT5G36160 - tyrosine aminotransferase; Transaminase involved in tyrosine breakdown.	0.28	0.00	0.02

	Converts tyrosine to p-hydroxyphenylpyruvate. Can catalyze the reverse reaction, using L-glutamate in vitro. Can convert phenylalanine to phenylpyruvate and catalyze the reverse reaction in vitro			
gi|241928169	XPL1 - XIPOTL 1; Catalyzes N-methylation of phosphoethanolamine, phosphomonomethylethanolamine and phosphodimethylethanolamine, the three methylation steps required to convert phosphoethanolamine to phosphocholine	0.26	0.01	0.06
gi|241942899	AT4G10500 - oxidoreductase, 2OG-Fe(II) oxygenase family protein	0.18	0.00	0.01

[1]Average fold change ratio defined as the average intensity of Al treated divided by the average intensity of the control. [2]The variance of the average fold change ration (**σ**[2]). [3]The standard error (**σ**/√N). (For details of the statistical analysis associated with the data presented in this table the reader is referred to the subsection, ”Quantitative protein expression profiles in the sorghum root tip regions” of the Results section of the manuscript.) To convert the GI numbers in this table to the new Accession.Version identifiers use EFetch as described at: https://ncbiinsights.ncbi.nlm.nih.gov/2016/12/06/converting-gi-numbers-to-accession-version/”.

For details of the statistical analysis associated with the data presented in this table, the reader is referred to the subsection "Quantitative protein expression profiles in the sorghum root tip regions" of the Results section of the paper.
