# Supplementary material for: Quantitative iTRAQ Proteomics Revealed Possible Roles for Antioxidant Proteins in Sorghum Aluminum Tolerance
Source: Front Plant Sci. 2017 Jan 9;7:2043. doi: 10.3389/fpls.2016.02043 (PMC5220100; doi:10.3389/fpls.2016.02043)
Supplement: Table S2 — Differentially expressed proteins (fold changes) in 5D BR007. [file Table2.PDF]

Table S2. Differentially Expressed Proteins (Fold Change) in 5D BR007.

Protein ID	Protein Description	Relative Protein Expression[1]	Variance[2]	SE[3]
gi|613447702	XRN4 - exoribonuclease 4; Possesses 5'->3' exoribonuclease activity. Acts as an endogenous post-transcriptional gene silencing (PTGS) suppressor. Degrades miRNA target cleavage products that lack a 5'-cap structure. Antagonizes the negative feedback regulation on EIN3 by promoting EBF1 and EBF2 mRNA decay, which consequently allows the accumulation of EIN3 protein to trigger the ethylene response	12.87	24.22	2.84
gi|241922980	LCR69 - defensin-like protein 2; Confers broad-spectrum resistance to pathogens	9.73	12.34	2.03
gi|241945166	AT3G28500 - 60S acidic ribosomal protein P2-3; Plays an important role in the elongation step of protein synthesis (By similarity)	7.86	2.93	0.99
gi|241936642	SERK2 - somatic embryogenesis receptor kinase 2; Serine/threonine-kinase involved in brassinosteroid-dependent and -independent signaling pathways. Acts redundantly with SERK1 as a control point for sporophytic development controlling male gametophyte production	6.50	0.13	0.21
gi|241946897	BCS1 - cytochrome BC1 synthesis	5.07	0.35	0.34
gi|241930937	PEX6 - peroxin 6; Involved in peroxisomal-targeting signal one (PTS1) and peroxisomal-targeting signal two (PTS2) protein import. Required for jasmonate biosynthesis. Necessary for the developmental elimination of obsolete peroxisome matix proteins. May form heteromeric AAA ATPase complexes required for the import of proteins. May be involved in PEX5 recycling	4.85	1.05	0.59

gi|241920881	AT2G15220 - basic secretory protein family protein	4.48	0.15	0.22
gi|241933008	CAT1 - catalase 1; Occurs in almost all aerobically respiring organisms and serves to protect cells from the toxic effects of hydrogen peroxide (By similarity)	4.44	0.58	0.44
gi|241930936	cICDH - isocitrate dehydrogenase; May supply 2-oxoglutarate for amino acid biosynthesis and ammonia assimilation via the glutamine synthetase/glutamate synthase (GS/GOGAT) pathway. May be involved in the production of NADPH to promote redox signaling or homeostasis in response to oxidative stress	4.19	1.47	0.70
gi|241924606	EULS3 - Euonymus lectin S3	3.93	0.16	0.23
gi|241942022	WAT1 - Walls Are Thin 1; Required for secondary wall formation in fibers, especially in short days conditions. Promotes indole metabolism and transport (e.g. tryptophan, neoglucobrassicin and auxin (indole-3-acetic acid)). May prevent salicylic-acid (SA) accumulation	3.91	0.73	0.49
gi|241933868	UGT85A1 - cytokinin-O-glucosyltransferase 2; Involved in the O-glucosylation of trans-zeatin and dihydrozeatin. Also active in vitro on cis-zeatin. Not active on N-glucosylated substrates	3.84	1.09	0.60
gi|257659117	PPDK - pyruvate, phosphate dikinase 1; Formation of phosphoenolpyruvate. May be involved in regulating the flux of carbon into starch and fatty acids of seeds and in the remobilization of nitrogen reserves in senescing leaves	3.72	0.11	0.19
gi|241946622	UGT73B3 - UDP-glucosyl transferase 73B3; Possesses quercetin 3-O-glucosyltransferase activity in vitro. Also active in vitro on benzoates and benzoate derivatives. Involved in stress or defense responses	3.70	1.37	0.68
gi|241933790	AT2G24600 - ankyrin repeat-containing protein	3.67	0.53	0.42
gi|241938127	XRN4 - exoribonuclease 4; Possesses 5'->3' exoribonuclease activity. Acts as an endogenous post-	3.67	0.04	0.11

	transcriptional gene silencing (PTGS) suppressor. Degrades miRNA target cleavage products that lack a 5'-cap structure. Antagonizes the negative feedback regulation on EIN3 by promoting EBF1 and EBF2 mRNA decay, which consequently allows the accumulation of EIN3 protein to trigger the ethylene response			
gi|241930137	AT1G60690 - NAD(P)-linked oxidoreductase-like protein	3.60	0.08	0.17
gi|241927971	CYP72A15 - cytochrome P450, family 72, subfamily A, polypeptide 15	3.43	0.07	0.15
gi|241934667	AT4G33540 - metallo-beta-lactamase family protein	3.42	0.25	0.29
gi|241939895	FRUCT5 - beta-fructofuranosidase 5; 6-fructan exohydrolase that can use phlein, levan, neokestose, levanbiose, 6-kestose, and 1-kestose as substrates	3.37	0.02	0.07
gi|241932602	COR47 - dehydrin COR47	3.35	0.05	0.13
gi|77744849	TIL - temperature-induced lipocalin	3.32	0.40	0.37
gi|241929511	AT1G13930 - uncharacterized protein	3.27	0.26	0.30
gi|241926454	OSM34 - osmotin 34	3.25	0.03	0.10
gi|241918895	CYP704A2 - cytochrome P450, family 704, subfamily A, polypeptide 2	3.22	0.59	0.44
gi|15529117	EP3 - chitinase	3.21	0.84	0.53
gi|241934580	NAC074 - NAC domain containing protein 74	3.20	0.65	0.46
gi|241935975	PRX52 - peroxidase 52; Removal of H(2)O(2), oxidation of toxic reductants, biosynthesis and degradation of lignin, suberization, auxin catabolism, response to environmental stresses such as wounding, pathogen attack and oxidative stress. These functions might be dependent on each isozyme/isoform in each plant tissue	3.20	0.06	0.14
gi|241918915	AT5G47550 - cysteine proteinase inhibitor 5; Specific inhibitor of cysteine proteinases. Probably involved in the regulation of endogenous processes and in defense against pests and pathogens (By similarity)	3.16	0.47	0.40

gi|241938592	AT1G62770 - plant invertase/pectin methylesterase inhibitor domain-containing protein	3.13	0.19	0.25
gi|241921284	AT1G65010 - uncharacterized protein	3.12	0.15	0.22
gi|241947322	HCHIB - chitinase; Defense against chitin containing fungal pathogens. Seems particularly implicated in resistance to jasmonate-inducing pathogens such as A.brassicicola. In vitro antifungal activity against T.reesei, but not against A.solani, F.oxysporum, S.sclerotiorum, G.graminis and P.megasperma	3.06	0.26	0.29
gi|241915454	AT5G11330 - FAD/NAD(P)-binding oxidoreductase family protein	3.02	0.41	0.37
gi|241937607	CYP79B3 - tryptophan N-hydroxylase 2; Converts tryptophan to indole-3-acetaldoxime, a precursor for tryptophan derived glucosinolates and indole-3- acetic acid (IAA)	3.02	1.74	0.76
gi|241920119	AATP1 - AAA-ATPase 1	2.96	0.02	0.09
gi|22208467	UGT73C7 - UDP-glucosyl transferase 73C7	2.91	0.05	0.13
gi|241935615	PR4 - hevein-like protein; Fungal growth inhibitors. Neither CB-HEL nor CD-HEL have chitinase activity, but both have antimicrobial activities. CD-HEL has RNase, but no DNase activity	2.89	0.13	0.21
gi|241935200	APY1 - apyrase 1; Catalyzes the hydrolysis of phosphoanhydride bonds of nucleoside tri- and di-phosphates. Substrate preference is ATP > ADP. Functions with APY2 to reduce extracellular ATP level which is essential for pollen germination and normal plant development. Plays a role in the regulation of stomatal function by modulating extracellular ATP levels in guard cells	2.86	0.22	0.27
gi|241929697	SRG1 - protein SRG1	2.85	0.19	0.25
gi|241926425	FLR1 - FLOR1	2.84	0.15	0.22
gi|241935376	AT4G32110 - beta-1,3-N-Acetylglucosaminyltransferase family protein	2.83	0.05	0.13

gi|241938664	AT3G05950 - germin-like protein subfamily 1 member 7; May play a role in plant defense. Probably has no oxalate oxidase activity even if the active site is conserved	2.82	0.16	0.23
gi|241935976	PRX52 - peroxidase 52; Removal of H(2)O(2), oxidation of toxic reductants, biosynthesis and degradation of lignin, suberization, auxin catabolism, response to environmental stresses such as wounding, pathogen attack and oxidative stress. These functions might be dependent on each isozyme/isoform in each plant tissue	2.81	0.07	0.15
gi|241935381	AT3G48770 - ATP/DNA binding protein	2.80	0.99	0.58
gi|241933691	AT1G14130 - 2-oxoglutarate (2OG) and Fe(II)-dependent oxygenase-like protein	2.80	0.09	0.17
gi|241932635	EXPB4 - expansin B4; May cause loosening and extension of plant cell walls by disrupting non-covalent bonding between cellulose microfibrils and matrix glucans. No enzymatic activity has been found (By similarity)	2.80	0.10	0.19
gi|241944360	AATP1 - AAA-ATPase 1	2.71	0.08	0.16
gi|241917136	AT4G30380 - putative EG45-like domain-containing protein 1; Might have a systemic role in water and solute homeostasis (By similarity)	2.71	0.14	0.21
gi|241925310	PIP2B - aquaporin PIP2-2; Water channel required to facilitate the transport of water across cell membrane. Plays an predominant role in root water uptake process in conditions of reduced transpiration, and in osmotic fluid transport. Its function is impaired by Hg(2+). Inhibited by cytosolic acidosis which occurs during anoxia in roots	2.69	0.32	0.33
gi|241918333	AT4G09770 - TRAF-like family protein	2.69	0.08	0.16
gi|241915743	AT3G28580 - AAA-type ATPase family protein	2.68	0.05	0.13
gi|241923808	AT5G10770 - aspartyl protease family protein	2.67	0.25	0.29

gi|241926637	PA2 - peroxidase 2; Removal of H(2)O(2), oxidation of toxic reductants, biosynthesis and degradation of lignin, suberization, auxin catabolism, response to environmental stresses such as wounding, pathogen attack and oxidative stress. These functions might be dependent on each isozyme/isoform in each plant tissue	2.66	1.17	0.62
gi|241946458	ACT7 - actin 7; Actins are highly conserved proteins that are involved in various types of cell motility and are ubiquitously expressed in all eukaryotic cells. Essential component of cell cytoskeleton; plays an important role in cytoplasmic streaming, cell shape determination, cell division, organelle movement and extension growth. This is considered as one of the vegetative actins which is involved in the regulation of hormone-induced plant cell proliferation and callus formation	2.64	0.05	0.13
gi|241928586	HSP70 - heat shock protein 70; Component of the Mediator complex, a coactivator involved in the regulated transcription of nearly all RNA polymerase II-dependent genes. Mediator functions as a bridge to convey information from gene-specific regulatory proteins to the basal RNA polymerase II transcription machinery. The Mediator complex, having a compact conformation in its free form, is recruited to promoters by direct interactions with regulatory proteins and serves for the assembly of a functional preinitiation complex with RNA polymerase II and the general transcription factors (By similarity)	2.62	0.11	0.19
gi|241925900	AT2G22420 - peroxidase; Removal of H(2)O(2), oxidation of toxic reductants, biosynthesis and degradation of lignin, suberization, auxin catabolism, response to environmental stresses such as wounding, pathogen attack and oxidative stress. These functions	2.62	0.11	0.19

	might be dependent on each isozyme/isoform in each plant tissue			
gi|241943862	AT5G13200 - GEM-like protein 5	2.59	0.02	0.08
gi|15529115	BGL2 - beta-1,3-glucanase 2; Implicated in the defense of plants against pathogens	2.58	0.15	0.23
gi|241922062	GAD - glutamate decarboxylase; Catalyzes the production of GABA. The calmodulin-binding is calcium-dependent and it is proposed that this may, directly or indirectly, form a calcium regulated control of GABA biosynthesis	2.57	0.09	0.18
gi|241925556	PPC1 - phosphoenolpyruvate carboxylase 1; Through the carboxylation of phosphoenolpyruvate (PEP) it forms oxaloacetate, a four-carbon dicarboxylic acid source for the tricarboxylic acid cycle. Contributes probably to the adaptation to inorganic phosophate (Pi) deprivation	2.55	0.04	0.11
gi|241920004	AT4G16100 - uncharacterized protein	2.51	0.30	0.31
gi|241943041	AT2G28790 - pathogenesis-related thaumatin-like protein	2.50	0.20	0.26
gi|241918805	EXPB2 - expansin B2; May cause loosening and extension of plant cell walls by disrupting non-covalent bonding between cellulose microfibrils and matrix glucans. No enzymatic activity has been found (By similarity)	2.50	0.07	0.16
gi|241930496	PPC3 - phosphoenolpyruvate carboxylase 3; Through the carboxylation of phosphoenolpyruvate (PEP) it forms oxaloacetate, a four-carbon dicarboxylic acid source for the tricarboxylic acid cycle	2.48	0.27	0.30
gi|241926632	RCI3 - peroxidase 3; Removal of H(2)O(2), oxidation of toxic reductants, biosynthesis and degradation of lignin, suberization, auxin catabolism, response to environmental stresses such as wounding, pathogen	2.48	0.06	0.14

	attack and oxidative stress. These functions might be dependent on each isozyme/isoform in each plant tissue			
gi|241937908	AT5G11420 - uncharacterized protein	2.45	0.08	0.17
gi|241915826	AT4G35880 - aspartyl protease family protein	2.43	0.08	0.17
gi|58978057	PYL12 - PYR1-like 12; Receptor for abscisic acid (ABA) required for ABA- mediated responses such as stomatal closure and germination inhibition. Inhibits the activity of group-A protein phosphatases type 2C (PP2Cs) when activated by ABA (By similarity)	2.41	0.04	0.12
gi|241942135	AT5G61820 - uncharacterized protein	2.39	0.04	0.11
gi|241921860	PYL6 - PYR1-like 6; Receptor for abscisic acid (ABA) required for ABA- mediated responses such as stomatal closure and germination inhibition. Inhibits the activity of group-A protein phosphatases type 2C (PP2Cs) when activated by ABA (By similarity)	2.37	0.08	0.16
gi|241936497	AT1G58170 - Disease resistance-responsive (dirigent-like protein) family protein	2.35	0.18	0.24
gi|241922385	AT5G60710 - C3H4 type zinc finger protein	2.35	0.05	0.14
gi|241915136	AT3G12710 - DNA-3-methyladenine glycosylase I	2.31	0.25	0.29
gi|241926919	AT5G45920 - GDSL esterase/lipase	2.26	0.34	0.34
gi|241921563	AT1G60420 - putative nucleoredoxin 1; Probable thiol-disulfide oxidoreductase required for pollen tube growth and pollen function in the pistil. Seems not to be required for in vitro pollen tube growth. May be involved in the generation of lipid signaling molecules in pistil	2.26	0.02	0.08
gi|241939085	OPR1 - 12-oxophytodienoate reductase 1; Specifically cleaves olefinic bonds in alpha,beta- unsaturated carbonyls and may be involved in detoxification or modification of these reactive compounds. May be involved in the biosynthesis or metabolism of oxylipin signaling molecules. In vitro, reduces 9R,13R-12-oxophyodienoic acid (9R,13R-OPDA) to 9R,13R-OPC-8:0, but only poorly 9S,13S-OPDA, the natural	2.25	0.10	0.19

	precursor of jasmonic acid. Can detoxify the explosive 2,4,6-trinitrotoluene (TNT) in vitro and in vivo by catalyzing its nitroreduction to form hydroxylamino-dinitrotoluene (HADNT)			
gi|58978001	PYL12 - PYR1-like 12; Receptor for abscisic acid (ABA) required for ABA- mediated responses such as stomatal closure and germination inhibition. Inhibits the activity of group-A protein phosphatases type 2C (PP2Cs) when activated by ABA (By similarity)	2.25	0.09	0.18
gi|241929234	ABCC3 - ATP-binding cassette C3; Pump for glutathione S-conjugates. Mediates the transport of glutathione conjugates such as chlorodinitrobenzene-GS (DNB-GS), and of chlorophyll catabolites such as Bn-NCC-1. Transports also heavy metals such as cadmium (Cd)	2.24	0.32	0.33
gi|241926347	AT4G26830 - O-glycosyl hydrolase-17	2.24	0.09	0.18
gi|241915714	RD21B - esponsive to dehydration 21B	2.23	0.11	0.20
gi|241922940	AT4G28360 - Ribosomal protein L22p/L17e family protein	2.21	0.07	0.15
gi|241924543	AT3G26770 - Rossmann-fold NAD(P)-binding domain-containing protein	2.21	0.07	0.15
gi|241921461	GSTU18 - glutathione S-transferase TAU 18; May be involved in the conjugation of reduced glutathione to a wide number of exogenous and endogenous hydrophobic electrophiles and have a detoxification role against certain herbicides (By similarity)	2.20	0.03	0.11
gi|241931686	PPC3 - phosphoenolpyruvate carboxylase 3; Through the carboxylation of phosphoenolpyruvate (PEP) it forms oxaloacetate, a four-carbon dicarboxylic acid source for the tricarboxylic acid cycle	2.18	0.03	0.10
gi|241944359	AATP1 - AAA-ATPase 1	2.12	0.04	0.12
gi|241932218	AT1G67900 - phototropic-responsive NPH3-like protein; May act as a substrate-specific adapter of an E3	2.12	0.07	0.15

	ubiquitin-protein ligase complex (CUL3-RBX1-BTB) which mediates the ubiquitination and subsequent proteasomal degradation of target proteins (By similarity)			
gi|219965357	BTI1 - reticulon-like protein B1; Plays a role in the Agrobacterium-mediated plant transformation via its interaction with VirB2, the major component of the T-pilus	2.12	0.04	0.12
gi|241929989	THI1 - thiazole biosynthetic enzyme; Involved in biosynthesis of the thiamine precursor thiazole. Catalyzes the conversion of NAD and glycine to adenosine diphosphate 5-(2-hydroxyethyl)-4-methylthiazole-2-carboxylic acid (ADT), an adenylated thiazole intermediate. The reaction includes an iron-dependent sulfide transfer from a conserved cysteine residue of the protein to a thiazole intermediate. The enzyme can only undergo a single turnover, which suggests it is a suicide enzyme. May have additional roles in adaptation to various stress conditions and in DNA damage tolerance	2.12	0.01	0.06
gi|241914822	AT3G46920 - octicosapeptide/Phox/Bem1p domain-containing protein kinase	2.12	0.05	0.13
gi|241927967	CYP72A14 - cytochrome P450, family 72, subfamily A, polypeptide 14	2.10	0.18	0.24
gi|241937783	PLAT1 - PLAT domain protein 1	2.10	0.10	0.18
gi|241943277	OSM34 - osmotin 34	2.08	0.03	0.10
gi|241922091	AOS - allene oxide synthase	2.07	0.05	0.13
gi|241922107	RD20 - caleosin-related protein; Probable calcium-binding peroxygenase. May be involved in the degradation of storage lipid in oil bodies, in abiotic stress-related signaling pathway and in drought tolerance through stomatal control under water deficit conditions	2.07	0.07	0.15

gi|241936501	AT4G35160 - O-methyltransferase family 2 protein	2.06	0.17	0.24
gi|241924663	CYP93D1 - cytochrome P450, family 93, subfamily D, polypeptide 1	2.06	0.12	0.20
gi|241946443	AT1G71950 - Proteinase inhibitor, propeptide	2.06	0.08	0.16
gi|241945753	AT5G48540 - cysteine-rich repeat secretory protein 55	2.06	0.01	0.05
gi|241925690	AT5G15870 - glycosyl hydrolase family 81 protein	2.05	0.06	0.15
gi|241934760	APY1 - apyrase 1; Catalyzes the hydrolysis of phosphoanhydride bonds of nucleoside tri- and di-phosphates. Substrate preference is ATP > ADP. Functions with APY2 to reduce extracellular ATP level which is essential for pollen germination and normal plant development. Plays a role in the regulation of stomatal function by modulating extracellular ATP levels in guard cells	2.05	0.03	0.10
gi|241930176	CYP72A15 - cytochrome P450, family 72, subfamily A, polypeptide 15	2.04	0.02	0.08
gi|241918016	AGO10 - ARGONAUTE 10; Involved in RNA-mediated post-transcriptional gene silencing (PTGS). Main component of the RNA-induced silencing complex (RISC) that binds to a short guide RNA such as a microRNA (miRNA) or small interfering RNA (siRNA). RISC uses the mature miRNA or siRNA as a guide for slicer-directed cleavage of homologous mRNAs to repress gene expression. Required for reliable formation of primary and axillary shoot apical meristems. Specifies leaf adaxial identity by repressing the miR165 and miR166 microRNAs in the embryonic shoot apex, in the shoot apical meristem (SAM) and leaf. R [...]	2.04	0.51	0.41
gi|241946908	AT5G12010 - uncharacterized protein	2.04	0.45	0.39
gi|241939008	AT1G01540 - putative serine/threonine-protein kinase	2.03	0.02	0.09
gi|241945206	RRP41 - exosome complex component RRP41	2.03	0.32	0.32

gi|241916072	AT5G46030 - transcription elongation factor 1-like protein; Transcription elongation factor implicated in the maintenance of proper chromatin structure in actively transcribed regions (By similarity)	2.03	0.34	0.33
gi|241935198	CHIA - chitinase A	2.03	0.13	0.21
gi|241919029	SCPL51 - serine carboxypeptidase-like 51; Probable carboxypeptidase (By similarity)	2.02	0.04	0.12
gi|241945212	AT5G02550 - uncharacterized protein	2.02	0.14	0.22
gi|219766905	DMR6 - DOWNY MILDEW RESISTANT 6	2.02	0.08	0.17
gi|241938280	AGO7 - ARGONAUTE7; Involved in RNA-mediated post-transcriptional gene silencing (PTGS). Main component of the RNA-induced silencing complex (RISC) that binds to a short guide RNA such as a microRNA (miRNA) or small interfering RNA (siRNA). RISC uses the mature miRNA or siRNA as a guide for slicer-directed cleavage of homologous mRNAs to repress gene expression. Required for the processing of 21 nucleotide trans-acting siRNAs (ta-siRNAs) derived from TAS3a transcripts. Associates preferentially with the microRNA (miRNA) miR390 which guides the cleavage of TAS3 precursor RNA. Seems to act as mi [...]	2.02	0.02	0.09
gi|241925402	DL1E - DYNAMIN-like 1E; Microtubule-associated force-producing protein that is targeted to the tubulo-vesicular network of the forming cell plate during cytokinesis. Plays also a major role in plasma membrane maintenance and cell wall integrity with an implication in vesicular trafficking, polar cell expansion, and other aspects of plant growth and development	2.01	0.03	0.09
gi|241935100	ChiC - class V chitinase	2.01	0.06	0.14
gi|241937907	AT5G11420 - uncharacterized protein	2.01	0.02	0.08
gi|241921452	ERD9 - glutathione S-transferase; Involved in light signaling, mainly phyA-mediated photomorphogenesis	1.99	0.01	0.07

	and in the integration of various phytohormone signals to modulate various aspects of plant development by affecting glutathione pools. In vitro, possesses glutathione S- transferase activity toward 1-chloro-2,4-dinitrobenzene (CDNB) and benzyl isothiocyanate (BITC)			
gi|241929458	UMAMIT4 - EamA domain-containing protein	1.99	0.69	0.48
gi|241922444	COX19-2 - cytochrome c oxidase 19-2	1.99	0.10	0.18
gi|241925303	EXPB4 - expansin B4; May cause loosening and extension of plant cell walls by disrupting non-covalent bonding between cellulose microfibrils and matrix glucans. No enzymatic activity has been found (By similarity)	1.99	0.03	0.10
gi|241916987	AT4G27250 - Rossmann-fold NAD(P)-binding domain-containing protein	1.99	0.05	0.13
gi|241946594	CYSB - cystatin B; Specific inhibitor of cysteine proteinases. Probably involved in the regulation of endogenous processes and in defense against pests and pathogens (By similarity)	1.98	0.13	0.21
gi|241936499	AT5G42510 - disease resistance-responsive, dirigent domain-containing protein	1.98	0.21	0.26
gi|241916338	AT5G39570 - uncharacterized protein	1.98	0.08	0.16
gi|241929173	PFK3 - phosphofructokinase 3	1.97	0.06	0.14
gi|241929023	ADCL - 4-amino-4-deoxychorismate lyase	1.96	0.05	0.13
gi|241933700	QUA1 - QUASIMODO 1; Alpha-1-4-D-galacturonosyltransferase involved in homogalacturonan (HGA) synthesis, a class of pectin which plays a role in cell adhesion	1.96	0.15	0.22
gi|241926310	BCS1 - cytochrome BC1 synthesis	1.96	0.02	0.07
gi|241919492	AT1G51340 - MATE efflux family protein; Citrate transporter critical for aluminum tolerance. Responsible for citrate exudation into the rhizosphere to protect roots from aluminum toxicity	1.94	0.44	0.38

gi|241936498	AT1G58170 - Disease resistance-responsive (dirigent-like protein) family protein	1.94	0.02	0.08
gi|241927225	CSLB01 - cellulose synthase-like B1; Thought to be a Golgi-localized beta-glycan synthase that polymerize the backbones of noncellulosic polysaccharides (hemicelluloses) of plant cell wall	1.94	0.40	0.37
gi|241924475	PER1 - 1-Cys peroxiredoxin PER1; Antioxidant protein that seems to contribute to the inhibition of germination during stress	1.94	0.09	0.17
gi|241920114	GLP4 - germin-like protein 4; May play a role in plant defense. Probably has no oxalate oxidase activity even if the active site is conserved	1.94	0.03	0.10
gi|241921038	UGT74F2 - UDP-glucosyltransferase 74F2; Glycosyltransferase that glucosylates benzoic acid and derivatives. Substrate preference is benzoic acid > salicylic acid (SA) > 3-hydroxybenzoic acid > 4-hydroxybenzoic acid. Catalyzes the formation of both SA 2-O-beta-D-glucoside (SAG) and SA glucose ester (SGE). Has high affinity for the tryptophan precursor anthranilate. Catalyzes the formation of anthranilate glucose ester. Is the major source of this activity in the plant	1.92	0.00	0.02
gi|241945837	DHNAT1 - DHNA-CoA thioesterase 1	1.92	0.03	0.10
gi|241930880	LOX1 - lipoxygenase 1; 9S-lipoxygenase that can use linoleic acid or linolenic acid as substrates. Plant lipoxygenases may be involved in a number of diverse aspects of plant physiology including growth and development, pest resistance, and senescence or responses to wounding. Catalyzes the hydroperoxidation of lipids containing a cis,cis-1,4-pentadiene structure. Function as regulators of root development by controlling the emergence of lateral roots	1.92	0.01	0.04

gi|241928417	AT2G41790 - insulysin; Peptidase that might be involved in pathogen or wound response. Not required for peroxisome biogenesis, indole-3-butyric acid (IBA) metabolism, fatty acid beta-oxidation or degradation of glyoxylate cycle enzymes during seedling development	1.92	0.01	0.06
gi|241937039	AT4G03090 - sequence-specific DNA binding transcription factor	1.91	0.22	0.27
gi|241920584	AT1G54410 - dehydrin family protein	1.90	0.18	0.24
gi|241938393	NUB1 - homolog of human NUB1	1.90	0.42	0.37
gi|241931221	ATSPP - signal peptide peptidase; Intramembrane-cleaving aspartic protease (I-CLiP) that cleaves type II membrane signal peptides in the hydrophobic plane of the membrane (By similarity). Catalyzes intramembrane proteolysis of some signal peptides after they have been cleaved from a preprotein, resulting in the release of the fragment from the ER membrane into the cytoplasm (By similarity). Plays a critical role in the development and function of the reproductive tissues, especially in pollen development	1.90	0.37	0.35
gi|241922789	AT3G22600 - bifunctional inhibitor/lipid-transfer protein/seed storage 2S albumin-like protein	1.89	0.05	0.13
gi|241921269	ERD3 - putative methyltransferase PMT21	1.89	0.12	0.20
gi|241936795	TAT7 - tyrosine aminotransferase	1.89	0.16	0.23
gi|241942396	PRX52 - peroxidase 52; Removal of H(2)O(2), oxidation of toxic reductants, biosynthesis and degradation of lignin, suberization, auxin catabolism, response to environmental stresses such as wounding, pathogen attack and oxidative stress. These functions might be dependent on each isozyme/isoform in each plant tissue	1.89	0.08	0.16
gi|241945401	UGT88A1 - UDP-glucosyl transferase 88A1; Possesses low quercetin 3-O-glucosyltransferase, 7-O-glucosyltransferase, 3'-O-glucosyltransferase and 4'-O-glucosyltransferase activities in vitro	1.89	0.06	0.14

gi|241936589	SDR1 - (+)-neomenthol dehydrogenase; Involved in basal resistance against pathogens	1.88	0.02	0.07
gi|241922032	ACR8 - ACT domain repeat 8	1.88	0.04	0.11
gi|241945024	FZL - FZO-like	1.88	0.18	0.24
gi|58977980	PYL6 - PYR1-like 6; Receptor for abscisic acid (ABA) required for ABA- mediated responses such as stomatal closure and germination inhibition. Inhibits the activity of group-A protein phosphatases type 2C (PP2Cs) when activated by ABA (By similarity)	1.87	0.06	0.14
gi|241932166	UGT85A2 - UDP-glucosyl transferase 85A2	1.86	0.06	0.14
gi|241921462	GSTU18 - glutathione S-transferase TAU 18; May be involved in the conjugation of reduced glutathione to a wide number of exogenous and endogenous hydrophobic electrophiles and have a detoxification role against certain herbicides (By similarity)	1.86	0.12	0.20
gi|241945971	AT5G14550 - Core-2/I-branching beta-1,6-N-acetylglucosaminyltransferase family protein	1.85	0.09	0.18
gi|241924554	AT5G56420 - F-box/FBD/LRR-repeat protein	1.85	0.23	0.28
gi|241927268	AT3G09410 - putative pectinacetylesterase	1.85	0.00	0.03
gi|241928209	CKL2 - casein kinase 1-like protein 2	1.85	0.05	0.13
gi|219891408	UGT84A1 - UDP-glycosyltransferase-like protein; Glucosyltransferase that glucosylates 4-coumarate, ferulate, caffeate, sinapate and cinnamate. Can glucosylate the phytotoxic xenobiotic compound 2,4,5-trichlorophenol (TCP)	1.85	0.03	0.10
gi|241939963	AT5G12890 - UDP-glycosyltransferase-like protein	1.84	0.01	0.05
gi|241945937	RCI3 - peroxidase 3; Removal of H(2)O(2), oxidation of toxic reductants, biosynthesis and degradation of lignin, suberization, auxin catabolism, response to environmental stresses such as wounding, pathogen attack and oxidative stress. These functions might be dependent on each isozyme/isoform in each plant tissue	1.84	0.05	0.13

gi|241937306	ABCC4 - ATP-binding cassette C4; Involved in the regulation of stomatal aperture. May function as a high-capacity pump for folates	1.84	0.04	0.12
gi|241917737	RS27A - ribosomal protein S27; May be involved in the elimination of damaged mRNA after UV irradiation	1.83	0.27	0.30
gi|241941737	AT2G43120 - pirin-like protein	1.83	0.11	0.19
gi|241942939	SYP131 - syntaxin 1B/2/3; Vesicle trafficking protein that functions in the secretory pathway (By similarity)	1.82	0.01	0.07
gi|241930520	AT4G37560 - formamidase	1.82	0.02	0.07
gi|241944027	AT2G47710 - adenine nucleotide alpha hydrolases-like protein	1.82	0.04	0.12
gi|241940006	AT1G71695 - peroxidase 12; Removal of H(2)O(2), oxidation of toxic reductants, biosynthesis and degradation of lignin, suberization, auxin catabolism, response to environmental stresses such as wounding, pathogen attack and oxidative stress. These functions might be dependent on each isozyme/isoform in each plant tissue	1.81	0.01	0.05
gi|241938626	AT4G10540 - Subtilase family protein	1.80	0.03	0.10
gi|241918260	AT5G23760 - putative copper transport protein	1.80	0.02	0.08
gi|241937081	STS - stachyose synthase; Transglycosidase operating by a ping-pong reaction mechanism. Involved in the synthesis of raffinose, a major soluble carbohydrate in seeds, roots and tubers (By similarity)	1.79	0.24	0.28
gi|241937145	AT1G03230 - aspartyl protease-like protein	1.79	0.01	0.05
gi|241915296	AT5G24165 - uncharacterized protein	1.79	0.03	0.10
gi|241935380	AT3G48770 - ATP/DNA binding protein	1.78	0.02	0.09
gi|241931374	AT1G51560 - Pyridoxamine 5'-phosphate oxidase-like protein	1.78	0.03	0.09
gi|241936021	AT1G50180 - NB-ARC domain-containing disease resistance protein; Potential disease resistance protein (By similarity)	1.78	0.05	0.14
gi|241931067	AT5G04080 - uncharacterized protein	1.78	0.08	0.17

gi|241917392	AT1G27400 - 60S ribosomal protein L17-1	1.78	0.21	0.26
gi|241926905	GH9C3 - endoglucanase 19	1.76	0.01	0.07
gi|241921836	CP12-1 - CP12 domain-containing protein 1; Acts as a linker essential in the assembly of a core complex of PRK/GAPDH. Coordinates the reversible inactivation of chloroplast enzymes GAPDH and PRK during darkness in photosynthetic tissues	1.76	0.02	0.09
gi|241930480	AT4G28300 - uncharacterized protein	1.76	0.02	0.08
gi|241919109	BIGYIN - protein BIGYIN1	1.75	0.04	0.11
gi|241940252	TOM2A - tobamovirus multiplication 2A	1.75	0.02	0.08
gi|241945927	AT3G17020 - universal stress protein (USP) family protein	1.75	0.02	0.07
gi|241939010	AT1G20110 - RING/FYVE/PHD zinc finger-containing protein	1.75	0.03	0.11
gi|241932143	AT1G47480 - alpha/beta-hydrolase domain-containing protein; Carboxylesterase acting on esters with varying acyl chain length (By similarity)	1.75	0.01	0.05
gi|241921745	PYD4 - PYRIMIDINE 4	1.75	0.01	0.05
gi|241928175	ABCB11 - P-glycoprotein 11	1.75	0.03	0.10
gi|241917477	EXPA4 - expansin A4; Causes loosening and extension of plant cell walls by disrupting non-covalent bonding between cellulose microfibrils and matrix glucans. No enzymatic activity has been found (By similarity)	1.75	0.09	0.18
gi|241930114	ABCG40 - ATP-binding cassette G40; May be a general defense protein (By similarity). Functions as a pump to exclude Pb(2+) ions and/or Pb(2+)- containing toxic compounds from the cytoplasm. Contributes to Pb(2+) ions resistance. Confers some resistance to the terpene sclareol	1.74	0.03	0.09
gi|241921453	GSTU18 - glutathione S-transferase TAU 18; May be involved in the conjugation of reduced glutathione to a wide number of exogenous and endogenous	1.74	0.20	0.26

	hydrophobic electrophiles and have a detoxification role against certain herbicides (By similarity)			
gi|241915718	AT1G73620 - pathogenesis-related thaumatin-like protein	1.74	0.06	0.15
gi|241939722	AT3G05950 - germin-like protein subfamily 1 member 7; May play a role in plant defense. Probably has no oxalate oxidase activity even if the active site is conserved	1.73	0.07	0.15
gi|241920677	SERPIN1 - serpin-ZX; Inhibits metacaspase-9 cysteine protease	1.73	0.02	0.08
gi|241931083	GLP5 - germin-like protein 5; May play a role in plant defense. Probably has no oxalate oxidase activity even if the active site is conserved	1.72	0.02	0.08
gi|241933760	CCH - copper chaperone	1.72	0.03	0.10
gi|219725304	SSI2 - suppressor of SA insensitive 2; Converts stearoyl-ACP to oleoyl-ACP by introduction of a cis double bond between carbons Delta(9) and Delta(10) of the acyl chain. Required for the activation of certain jasmonic acid (JA)- mediated responses and the repression of the salicylic acid (SA) signaling pathway	1.72	0.07	0.15
gi|4680212	SP1L2 - SPIRAL1-like2; Acts redundantly with SPR1 in maintaining the cortical microtubules organization essential for anisotropic cell growth	1.72	0.08	0.17
gi|241942714	ATGSTF13 - Glutathione S-transferase-like protein; May be involved in the conjugation of reduced glutathione to a wide number of exogenous and endogenous hydrophobic electrophiles and have a detoxification role against certain herbicides (By similarity)	1.71	0.04	0.12
gi|241946476	LAX1 - like AUXIN RESISTANT 1; Carrier protein involved in proton-driven auxin influx. Mediates the formation of auxin gradient from developing leaves (site of auxin biosynthesis) to tips by contributing to the	1.71	0.05	0.12

	loading of auxin in vascular tissues and facilitating acropetal (base to tip) auxin transport within inner tissues of the root apex, and basipetal (tip to base) auxin transport within outer tissues of the root apex (By similarity)			
gi|241918799	EXPB2 - expansin B2; May cause loosening and extension of plant cell walls by disrupting non-covalent bonding between cellulose microfibrils and matrix glucans. No enzymatic activity has been found (By similarity)	1.71	0.03	0.10
gi|241944295	T6J19.6 - homolog of anti-oxidant 1	1.71	0.11	0.19
gi|241919028	SCPL51 - serine carboxypeptidase-like 51; Probable carboxypeptidase (By similarity)	1.71	0.22	0.27
gi|241941701	UGT88A1 - UDP-glucosyl transferase 88A1; Possesses low quercetin 3-O-glucosyltransferase, 7-O-glucosyltransferase, 3'-O-glucosyltransferase and 4'-O-glucosyltransferase activities in vitro	1.71	0.00	0.04
gi|241932874	AT1G31430 - pentatricopeptide repeat-containing protein	1.71	0.24	0.28
gi|241918032	AAT1 - amino acid transporter 1; High-affinity permease involved in the transport of the cationic amino acids (e.g. arginine, lysine, histidine, citrulline, valine, and glutamate). Transport mostly basic amino- acids, and, to a lower extent neutral and acidic amino-acids. May function as a proton symporter	1.70	0.04	0.11
gi|241945361	VOZ1 - vascular plant one zinc finger protein; Transcriptional activator acting positively in the phytochrome B signaling pathway. Functions redundantly with VOZ2 to promote flowering downstream of phytochrome B (phyB). Down- regulates 'FLOWERING LOCUS C' (FLC) and up-regulates 'FLOWERING LOCUS T' (FT). Binds to the 38-bp cis-acting region of the AVP1 gene. Interacts with phyB in	1.70	0.06	0.14

	the cytoplasm and is translocated to the nucleus at signal transmission, where it is subjected to degradation in a phytochrome-dependent manner			
gi|241919730	AT5G43950 - uncharacterized protein	1.70	0.25	0.29
gi|241946533	LSH5 - uncharacterized protein	1.70	0.06	0.14
gi|241917347	NFD2 - NUCLEAR FUSION DEFECTIVE 2	1.69	0.10	0.18
gi|241917045	BPM2 - BTB-POZ and MATH domain 2; May act as a substrate-specific adapter of an E3 ubiquitin-protein ligase complex (CUL3-RBX1-BTB) which mediates the ubiquitination and subsequent proteasomal degradation of target proteins	1.69	0.02	0.08
gi|241932045	ELM1 - uncharacterized protein	1.68	0.06	0.15
gi|241919487	TUA3 - tubulin alpha-3; Tubulin is the major constituent of microtubules. It binds two moles of GTP, one at an exchangeable site on the beta chain and one at a non-exchangeable site on the alpha chain	1.68	0.03	0.10
gi|241936226	FBA2 - fructose-bisphosphate aldolase 2	1.68	0.04	0.12
gi|241935756	VPS15 - vacuolar protein sorting 15	1.68	0.23	0.28
gi|241931078	GSTU7 - glutathione S-transferase tau 7; May be involved in the conjugation of reduced glutathione to a wide number of exogenous and endogenous hydrophobic electrophiles and have a detoxification role against certain herbicides (By similarity)	1.68	0.10	0.18
gi|241917554	AT4G38810 - EF-hand, calcium binding motif-containing protein	1.68	0.16	0.23
gi|241934474	AT3G12020 - kinesin motor protein-like protein	1.67	0.20	0.26
gi|241917020	VHA-A - vacuolar ATP synthase subunit A; Catalytic subunit of the peripheral V1 complex of vacuolar ATPase. V-ATPase vacuolar ATPase is responsible for acidifying a variety of intracellular compartments in eukaryotic cells	1.67	0.07	0.15
gi|241926255	NDPK1 - nucleoside diphosphate kinase 1; Major role in the synthesis of nucleoside triphosphates other than	1.67	0.01	0.07

	ATP. The ATP gamma phosphate is transferred to the NDP beta phosphate via a ping-pong mechanism, using a phosphorylated active-site intermediate			
gi|241932903	PIP1;4 - putative aquaporin PIP1-4; Water channel required to facilitate the transport of water across cell membrane. Essential for the water permeability of the plasma membrane and for the morphology of the root system. Its function is impaired by Hg(2+). Inhibited by cytosolic acidosis which occurs during anoxia in roots	1.67	0.06	0.14
gi|241939552	DHDPS2 - dihydrodipicolinate synthase; Catalyzes the condensation of (S)-aspartate-beta- semialdehyde [(S)-ASA] and pyruvate to 4-hydroxy- tetrahydrodipicolinate (HTPA) (By similarity)	1.67	0.02	0.08
gi|241927544	AT1G03180 - uncharacterized protein	1.67	0.04	0.11
gi|241938337	BAN - BANYULS; Involved in the biosynthesis of condensed tannins. Converts cyanidin into (-)-epicatechin as the major product	1.67	0.04	0.12
gi|241921861	PYL6 - PYR1-like 6; Receptor for abscisic acid (ABA) required for ABA- mediated responses such as stomatal closure and germination inhibition. Inhibits the activity of group-A protein phosphatases type 2C (PP2Cs) when activated by ABA (By similarity)	1.67	0.05	0.13
gi|241923619	TET8 - tetraspanin8; May be involved in the regulation of cell differentiation (By similarity)	1.66	0.00	0.03
gi|241927139	AT3G14880 - uncharacterized protein	1.66	0.08	0.17
gi|241914625	ATB2 - NAD(P)-linked oxidoreductase-like protein	1.66	0.03	0.10
gi|241940003	AT1G71695 - peroxidase 12; Removal of H(2)O(2), oxidation of toxic reductants, biosynthesis and degradation of lignin, suberization, auxin catabolism, response to environmental stresses such as wounding, pathogen attack and oxidative stress. These functions might be dependent on each isozyme/isoform in each plant tissue	1.66	0.03	0.09

gi|241929733	UXS4 - UDP-xylose synthase 4; Catalyzes the NAD-dependent decarboxylation of UDP- glucuronic acid to UDP-xylose. Necessary for the biosynthesis of the core tetrasaccharide in glycosaminoglycan biosynthesis (By similarity)	1.66	0.01	0.06
gi|241926135	AT1G20225 - TRX domain-containing protein	1.66	0.04	0.11
gi|241930836	AT5G09760 - Putative pectinesterase/pectinesterase inhibitor 51; Acts in the modification of cell walls via demethylesterification of cell wall pectin (By similarity)	1.66	0.07	0.15
gi|241930599	AT2G27730 - copper ion binding protein	1.65	0.00	0.04
gi|241937109	AT4G02540 - cysteine/histidine-rich C1 domain-containing protein	1.65	0.20	0.26
gi|241917923	AT5G09570 - Cox19-like CHCH family protein	1.65	0.11	0.19
gi|241915748	AT4G29890 - choline monooxygenase; Catalyzes the first step of the osmoprotectant glycine betaine synthesis (By similarity)	1.64	0.12	0.20
gi|241930087	AT4G17520 - plasminogen activator inhibitor 1 RNA-binding protein	1.64	0.01	0.07
gi|241931185	AT4G17260 - L-lactate dehydrogenase	1.64	0.05	0.13
gi|241918606	AT5G66150 - Glycosyl hydrolase family 38 protein	1.63	0.01	0.05
gi|241936195	AT1G64010 - serine protease inhibitor-like protein	1.63	0.07	0.15
gi|241931032	LBO1 - LATERAL BRANCHING OXIDOREDUCTASE 1	1.63	0.08	0.16
gi|241924411	AMI1 - amidase 1; Amidase involved in auxin biosynthesis. Converts indole- 3-acetamide into indole-3-acetic acid. Can also use L-asparagine, oleamide and 1-naphtalene-acetamide as substrates, but not indole-3-acetonitrile or indole-3-acetyl-L-aspartic acid	1.63	0.06	0.14
gi|241916473	UGT71C4 - UDP-glucosyl transferase 71C4; Possesses quercetin 3-O-glucosyltransferase and 7-O-glucosyltransferase activities in vitro. Also active in vitro on benzoates and benzoate derivatives	1.63	0.06	0.15
gi|241941251	PUB4 - plant U-box 4; Functions as an E3 ubiquitin ligase (By similarity)	1.63	0.05	0.13

gi|241943470	LP1 - lipid transfer protein 1; Plant non-specific lipid-transfer proteins transfer phospholipids as well as galactolipids across membranes. May play a role in wax or cutin deposition in the cell walls of expanding epidermal cells and certain secretory tissues	1.62	0.04	0.11
gi|241939218	AT1G14130 - 2-oxoglutarate (2OG) and Fe(II)-dependent oxygenase-like protein	1.62	0.02	0.07
gi|241917393	AT1G27400 - 60S ribosomal protein L17-1	1.62	0.07	0.15
gi|241923666	AT5G49690 - UDP-glycosyltransferase-like protein	1.62	0.02	0.07
gi|241920098	AT3G57490 - 40S ribosomal protein S2-4	1.62	0.10	0.18
gi|241917650	ADF6 - actin depolymerizing factor 6; Actin-depolymerizing protein. Severs actin filaments (F- actin) and binds to actin monomers	1.62	0.03	0.09
gi|241940921	AT5G23950 - calcium-dependent lipid-binding domain-containing protein	1.61	0.02	0.08
gi|241925200	RAB11c - RAB GTPase 11C; Intracellular vesicle trafficking and protein transport (By similarity)	1.61	0.10	0.18
gi|241920936	AT1G04430 - putative methyltransferase PMT8	1.61	0.07	0.16
gi|241924277	AT1G20110 - RING/FYVE/PHD zinc finger-containing protein	1.61	0.02	0.08
gi|241937255	AT2G45630 - D-isomer specific 2-hydroxyacid dehydrogenase-like protein	1.61	0.03	0.10
gi|241937821	EMB1873 - agmatine deiminase; Mediates the hydrolysis of agmatine into N- carbamoylputrescine in the arginine decarboxylase (ADC) pathway of putrescine biosynthesis, a basic polyamine	1.61	0.02	0.08
gi|241937344	AT1G06620 - 1-aminocyclopropane-1-carboxylate oxidase-1	1.60	0.02	0.07
gi|241930041	HIR1 - HYPERSENSITIVE-INDUCED RESPONSE PROTEIN 1	1.60	0.02	0.08
gi|241941805	ABCA2 - ATP-binding cassette A2	1.60	0.03	0.10
gi|241946875	AT4G28000 - AAA-type ATPase family protein	1.60	0.04	0.12

gi|241926232	SCPL49 - carboxypeptidase; Probable carboxypeptidase (By similarity)	1.60	0.01	0.05
gi|241921364	AT5G58740 - nudC domain-containing protein	1.60	0.05	0.13
gi|241936668	SNX1 - sorting nexin 1; Plays a role in vesicular protein sorting. Acts at the crossroads between the secretory and endocytic pathways. Is involved in the endosome to vacuole protein transport via its interaction with the BLOS1/2 proteins and, as component of the membrane-associated retromer complex, is also involved in endosome-to-Golgi retrograde transport. Required for the auxin- carrier protein PIN2 sorting to the lytic vacuolar pathway and the trafficking of several plasma membrane proteins. Also involved in the efficient sorting of seed storage protein globulin 12S	1.60	0.15	0.22
gi|241914900	HON4 - DNA-binding protein HMR1-like protein	1.60	0.09	0.17
gi|241921608	CYP71A25 - cytochrome P450 71A25	1.60	0.16	0.23
gi|219898271	RPS13A - ribosomal protein S13A	1.59	0.10	0.19
gi|241916918	CRL1 - CCR(Cinnamoyl coA:NADP oxidoreductase)-like 1	1.59	0.16	0.23
gi|241930399	ABCB15 - ATP-binding cassette B15	1.59	0.07	0.15
gi|241945767	DHAR2 - dehydroascorbate reductase 2; Exhibits glutathione-dependent thiol transferase and dehydroascorbate (DHA) reductase activities. Key component of the ascorbate recycling system. Involved in the redox homeostasis, especially in scavenging of ROS under oxidative stresses. Plays a role in ozone tolerance	1.59	0.02	0.08
gi|241940843	AT2G03200 - aspartyl protease-like protein	1.58	0.06	0.14
gi|241919699	PHT1;7 - phosphate transporter 1;7; High-affinity transporter for external inorganic phosphate (By similarity)	1.58	0.02	0.08
gi|241928760	BGLU42 - beta glucosidase 42	1.58	0.02	0.08

gi|118426387	GPT2 - glucose-6-phosphate/phosphate translocator 2; Glucose 6-phosphate (Glc6P) transporter. Transports also inorganic phosphate, 3-phosphoglycerate, triose phosphates and, to a leser extent, phosphoenolpyruvate. Responsible for the transport of Glc6P into plastids of heterotrophic tissues where it can be used as a carbon source for starch biosynthesis, as substrate for fatty acid biosynthesis or as substrate for NADPH generation via the oxidative pentose phosphate pathway (OPPP). Required for dynamic acclimation of photosynthesis	1.58	0.04	0.11
gi|241937673	AT4G25740 - 40S ribosomal protein S10-1	1.57	0.04	0.12
gi|241931077	GSTU8 - glutathione S-transferase TAU 8; May be involved in the conjugation of reduced glutathione to a wide number of exogenous and endogenous hydrophobic electrophiles and have a detoxification role against certain herbicides (By similarity)	1.57	0.03	0.10
gi|241914953	ANNAT7 - annexin 7	1.57	0.00	0.04
gi|241933953	VPS20.2 - vacuolar protein sorting-associated protein 20-2; Component of the ESCRT-III complex, which is required for multivesicular bodies (MVBs) formation and sorting of endosomal cargo proteins into MVBs. The ESCRT-III complex is probably involved in the concentration of MVB cargo (By similarity)	1.57	0.01	0.05
gi|241945324	XBCP3 - xylem bark cysteine peptidase 3	1.57	0.01	0.06
gi|241939407	TRX-M4 - thioredoxin M4; Thiol-disulfide oxidoreductase involved in the redox regulation of enzyme of the oxidative pentose phosphate pathway. Under reducing conditions, inhibits the glucose-6-phosphate dehydrogenase	1.57	0.01	0.06
gi|241944051	AT5G61310 - putative cytochrome c oxidase subunit 5C-3; This protein is one of the nuclear-coded polypeptide chains of cytochrome c oxidase, the	1.57	0.05	0.13

	terminal oxidase in mitochondrial electron transport (By similarity)			
gi|241919030	SCPL51 - serine carboxypeptidase-like 51; Probable carboxypeptidase (By similarity)	1.57	0.07	0.15
gi|241943126	AT5G64130 - cAMP-regulated phosphoprotein 19-related protein	1.57	0.01	0.06
gi|241928168	XPL1 - XIPOTL 1; Catalyzes N-methylation of phosphoethanolamine, phosphomonomethylethanolamine and phosphodimethylethanolamine, the three methylation steps required to convert phosphoethanolamine to phosphocholine	1.57	0.03	0.09
gi|241923592	AT2G32070 - putative CCR4-associated factor 1-7; Ubiquitous transcription factor required for a diverse set of processes. It is a component of the CCR4 complex involved in the control of gene expression (By similarity)	1.57	0.05	0.13
gi|241939983	AAE3 - ACYL-ACTIVATING ENZYME 3	1.56	0.00	0.01
gi|241938418	COS1 - COI1 SUPPRESSOR1; Catalyzes the formation of 6,7-dimethyl-8- ribityllumazine by condensation of 5-amino-6-(D- ribitylamino)uracil with 3,4-dihydroxy-2-butanone 4-phosphate. This is the penultimate step in the biosynthesis of riboflavin (By similarity)	1.56	0.01	0.04
gi|241932326	SCPL34 - carboxypeptidase D; Probable carboxypeptidase (By similarity)	1.56	0.15	0.23
gi|241932249	EP3 - chitinase	1.56	0.01	0.05
gi|241934563	AT1G80030 - molecular chaperone Hsp40/DnaJ-like protein	1.56	0.06	0.14
gi|241928202	AT2G19730 - 60S ribosomal protein L28-1	1.56	0.09	0.18
gi|241922873	RNL - RNAligase	1.56	0.07	0.15
gi|241940608	NUDX15 - nudix hydrolase 15; Coenzyme A diphosphatase which mediates the cleavage of oxidized CoA. Can use malonyl-CoA, hexanoyl-CoA, lauroyl-	1.55	0.06	0.14

	CoA, myristoyl-CoA and palmitoyl-CoA as substrates, but not isobutyryl- CoA or propionyl-CoA			
gi|241943292	AT1G34360 - translation initiation factor IF-3	1.55	0.06	0.14
gi|241945986	CYP71B37 - cytochrome P450 71B37	1.55	0.12	0.20
gi|219965359	BTI2 - reticulon-like protein B2; Plays a role in the Agrobacterium-mediated plant transformation via its interaction with VirB2, the major component of the T-pilus	1.55	0.06	0.15
gi|241945083	CXE17 - carboxyesterase 17; Carboxylesterase acting on esters with varying acyl chain length (By similarity)	1.55	0.01	0.06
gi|241940008	AT1G71695 - peroxidase 12; Removal of H(2)O(2), oxidation of toxic reductants, biosynthesis and degradation of lignin, suberization, auxin catabolism, response to environmental stresses such as wounding, pathogen attack and oxidative stress. These functions might be dependent on each isozyme/isoform in each plant tissue	1.55	0.04	0.12
gi|241944621	mtLPD1 - dihydrolipoyl dehydrogenase 1; Lipoamide dehydrogenase is a component of the glycine decarboxylase (GDC) or glycine cleavage system as well as of the alpha-ketoacid dehydrogenase complexes. LPD1 is probably the protein most often associated with the glycine decarboxylase complex while LPD2 is probably incorporated into alpha-ketoacid dehydrogenase complexes	1.55	0.01	0.07
gi|219898273	RPS13A - ribosomal protein S13A	1.55	0.04	0.12
gi|241942270	SVL1 - SHV3-like 1	1.55	0.04	0.12
gi|241930433	ERA1 - ENHANCED RESPONSE TO ABA 1; Catalyzes the transfer of a farnesyl moiety from farnesyl pyrophosphate to a cysteine at the fourth position from the C-terminus of several proteins. The beta subunit is responsible for peptide-binding (By similarity)	1.55	0.12	0.20
gi|241939289	EP3 - chitinase	1.55	0.01	0.05

gi|241931140	AT5G22320 - leucine-rich repeat-containing protein	1.54	0.07	0.16
gi|241920635	NSF - vesicle-fusing ATPase; Involved in vesicle-mediated transport. The ATPase activity of NSF serves to disassemble the SNARE complex, freeing the components for subsequent pairing and fusion events	1.54	0.00	0.03
gi|241924065	AT4G34880 - Amidase family protein	1.54	0.02	0.09
gi|241946839	DSEL - lipase class 3 family protein; Acylhydrolase that catalyzes the hydrolysis of 1,3- diacylglycerol (1,3-DAG) and 1-monoacylglycerol (1-MAG) at the sn- 1 position. High activity toward 1,3-DAG and 1-MAG, but low activity toward 1,2-diacylglycerol (1,2-DAG) and 1-lysophosphatidylcholine (1-LPC), and no activity toward phosphatidylcholine (PC), monogalactosyldiacylglycerol (MGDG), digalactosyldiacylglycerol (DGDG), triacylglycerol (TAG) and 2- monoacylglycerol (2-MAG). May be involved in the negative regulation of seedling establishment by inhibiting the breakdown, beta-oxidation and mobi [...]	1.54	0.03	0.10
gi|241937676	ARA12 - subtilisin-like protease; Serine protease. Has a substrate preference for the hydrophobic residues Phe and Ala and the basic residue Asp in the P1 position, and for Asp, Leu or Ala in the P1' position	1.54	0.03	0.10
gi|241916324	HVA22A - HVA22 homologue A	1.54	0.01	0.05
gi|241921870	LAC12 - laccase 12; Lignin degradation and detoxification of lignin-derived products (By similarity)	1.54	0.11	0.19
gi|241924322	FH6 - formin homolog 6; Might be involved in the organization and polarity of the actin cytoskeleton	1.53	0.07	0.15
gi|241919321	DSI-1VOC - dessication-induced 1VOC-like protein	1.53	0.05	0.13
gi|241926293	APR3 - APS reductase 3; Reduces sulfate for Cys biosynthesis. Substrate preference is adenosine-5'-phosphosulfate (APS) >> 3'- phosphoadenosine-5'-phosphosulfate (PAPS). Uses glutathione or DTT as source of protons	1.53	0.07	0.16

gi|241914875	AT5G04800 - 40S ribosomal protein S17-4	1.53	0.09	0.18
gi|241947020	LAF3 - LONG AFTER FAR-RED 3	1.53	0.04	0.11
gi|241937689	F10A8.5 - bidirectional amino acid transporter 1; May play a role in primary carbon metabolism and plant growth, by mediating the transport of GABA from the cytosol to mitochondria. When expressed in a heterologous system (yeast), imports Arg and Ala across the plasma membrane and exports Lys and Glu, but does not transport proline	1.53	0.05	0.13
gi|241922079	AT1G23780 - F-box protein SKIP22; Component of SCF(ASK-cullin-F-box) E3 ubiquitin ligase complexes, which may mediate the ubiquitination and subsequent proteasomal degradation of target proteins (By similarity)	1.53	0.06	0.14
gi|241933116	ARA1 - arabinose kinase; Arabinose kinase. Involved in the salvage pathway which converts free L-arabinose to UDP-L-arabinose. May play a role in arabinose transport	1.53	0.07	0.15
gi|241924078	PAP27 - purple acid phosphatase 27	1.53	0.00	0.02
gi|257632199	AT3G14360 - lipase class 3 family protein	1.52	0.02	0.09
gi|241921980	AT1G53540 - HSP20-like chaperone	1.52	0.03	0.10
gi|241932292	GRXC2 - glutaredoxin C2; Has a glutathione-disulfide oxidoreductase activity in the presence of NADPH and glutathione reductase. Reduces low molecular weight disulfides and proteins (By similarity)	1.52	0.03	0.11
gi|241917244	AT2G46690 - SAUR-like auxin-responsive protein	1.52	0.04	0.11
gi|241926550	AT1G70600 - 60S ribosomal protein L27a-3	1.52	0.09	0.17
gi|241942168	PAP27 - purple acid phosphatase 27	1.52	0.01	0.05
gi|241921951	HSP70 - heat shock protein 70; Component of the Mediator complex, a coactivator involved in the regulated transcription of nearly all RNA polymerase II-dependent genes. Mediator functions as a bridge to convey information from gene-specific regulatory	1.52	0.01	0.04

	proteins to the basal RNA polymerase II transcription machinery. The Mediator complex, having a compact conformation in its free form, is recruited to promoters by direct interactions with regulatory proteins and serves for the assembly of a functional preinitiation complex with RNA polymerase II and the general transcription factors (By similarity)			
gi|241935070	AT2G31810 - ACT domain-containing small subunit of acetolactate synthase protein; Regulatory subunit of acetohydroxy-acid synthase. Involved in the feed-back inhibition by branched-chain amino acids. Contains 2 repeats, each of them being able to activate partially the catalytic subunit. The enzyme reconstituted with the first repeat is inhibited by leucine, but not by valine or isoleucine and the enzyme reconstituted with the second repeat is not inhibited by any branched-chain amino acid	1.51	0.00	0.02
gi|241926932	HBP1 - haem-binding protein 1	1.51	0.01	0.06
gi|241933087	AT5G40450 - uncharacterized protein	1.51	0.02	0.08
gi|241937766	AT2G14095 - uncharacterized protein	1.51	0.08	0.16
gi|241916476	GT72B1 - hydroquinone glucosyltransferase; Bifunctional O-glycosyltransferase and N-glycosyltransferase that can detoxify xenobiotics. Possesses high activity to metabolize the peristent pollutants 2,4,5- trichlorophenol (TCP) and 3,4-dichloroaniline (DCA). Also active on benzoates and benzoate derivatives in vitro	1.51	0.03	0.11
gi|241938565	AT1G22610 - C2 domain-containing protein	1.51	0.07	0.15
gi|241935109	AT2G44130 - F-box/kelch-repeat protein	1.51	0.03	0.10
gi|241931076	GSTU8 - glutathione S-transferase TAU 8; May be involved in the conjugation of reduced glutathione to a wide number of exogenous and endogenous	1.51	0.04	0.12

	hydrophobic electrophiles and have a detoxification role against certain herbicides (By similarity)			
gi|241933017	APG5 - AUTOPHAGY 5; Required for autophagy. Conjugation to ATG12 is essential for plant nutrient recycling	1.51	0.04	0.11
gi|241943244	CHX19 - cation/H+ exchanger 19; May operate as a cation/H(+) antiporter (By similarity)	1.51	0.02	0.07
gi|241931518	AT4G35220 - Cyclase family protein	1.51	0.02	0.09
gi|241922218	AT1G28200.1 - GEM-like protein 1	1.50	0.01	0.05
gi|241934682	SAUL1 - U-box domain-containing protein 44; Functions as an E3 ubiquitin-protein ligase. Prevents premature senescence probably by targeting proteins involved in this process for degradation. Promotes the degradation of AAO3 and thus represses abscisic acid (ABA) biosynthesis	0.67	0.01	0.05
gi|241915410	BGAL1 - beta galactosidase 1	0.67	0.02	0.07
gi|241917415	AT1G34300 - lectin protein kinase-like protein	0.67	0.02	0.09
gi|241937700	MCM3 - MINICHROMOSOME MAINTENANCE 3; Acts as a factor that allows the DNA to undergo a single round of replication per cell cycle. Required for DNA replication and cell proliferation. May act as a component of the MCM complex which is the putative replicative helicase of the replication licensing system in eukaryotic cells	0.67	0.01	0.06
gi|241945451	AT1G15400 - uncharacterized protein	0.67	0.01	0.06
gi|241942310	AT5G53070 - Ribosomal protein L9/RNase H1	0.67	0.01	0.04
gi|241920230	TUA6 - Tubulin alpha-6; Tubulin is the major constituent of microtubules. It binds two moles of GTP, one at an exchangeable site on the beta chain and one at a non-exchangeable site on the alpha chain (By similarity)	0.66	0.00	0.01
gi|241917217	AT1G36240 - large subunit ribosomal protein L30e	0.66	0.01	0.06
gi|241945557	ERDL6 - ERD6-like 6; Sugar transporter (Potential)	0.66	0.01	0.06
gi|241932767	AT1G74270 - 60S ribosomal protein L35a-3	0.66	0.01	0.06

gi|241915490	ZAC - ADP-ribosylation factor GTPase-activating protein AGD12; GTPase-activating protein (GAP) for ADP ribosylation factor (ARF). Binds phosphatidylinositol 3-monophosohate (PI-3-P) and anionic phospholipids	0.66	0.00	0.03
gi|241915657	AT1G30690 - patellin-4; Carrier protein that may be involved in membrane- trafficking events associated with cell plate formation during cytokinesis. Binds to some hydrophobic molecules such as phosphoinositides and promotes their transfer between the different cellular sites (By similarity)	0.66	0.00	0.03
gi|241932891	FIB2 - fibrillarin 2; Component of the Mediator complex, a coactivator involved in the regulated transcription of nearly all RNA polymerase II-dependent genes. Mediator functions as a bridge to convey information from gene-specific regulatory proteins to the basal RNA polymerase II transcription machinery. The Mediator complex, having a compact conformation in its free form, is recruited to promoters by direct interactions with regulatory proteins and serves for the assembly of a functional preinitiation complex with RNA polymerase II and the general transcription factors	0.66	0.01	0.04
gi|241941527	NUC-L1 - nucleolin; Involved in pre-rRNA processing and ribosome assembly. Is associated with intranucleolar chromatin and pre-ribosomal particles and plays a role in controlling activation and repression of a specific subset of rRNA genes located in distinctive nucleolar organizer regions. Binds specifically rDNA chromatin and may be required to maintain rDNA chromatin structure, but is probably not required for the overall histone methylation status of 45S rRNA genes	0.66	0.01	0.06
gi|241927543	AT5G15180 - peroxidase 56; Removal of H(2)O(2), oxidation of toxic reductants, biosynthesis and	0.66	0.01	0.05

	degradation of lignin, suberization, auxin catabolism, response to environmental stresses such as wounding, pathogen attack and oxidative stress. These functions might be dependent on each isozyme/isoform in each plant tissue			
gi|241931429	AT1G50670 - ubiquitin thioesterase OTU1	0.66	0.00	0.04
gi|241939802	XYL4 - beta-D-xylosidase 4; Beta-D-xylosidase showing an optimal efficiency with the natural substrate xylobiose	0.66	0.00	0.02
gi|241943985	ACP4 - acyl carrier protein 4; Carrier of the growing fatty acid chain in fatty acid biosynthesis that plays a major role in the biosynthesis of fatty acids in leaves. Required for the biosynthesis of chloroplast photosynthetic membrane lipids such as monogalactosyldiacylglycerol, digalactosyldiacylglycerol and phosphatidylglycerol. Is essential for the biosynthesis of the cuticular wax and cutin polymers in leaves, and for the establishment of systemic acquired resistance (SAR)	0.66	0.01	0.04
gi|241941886	LTA2 - 2-oxoacid dehydrogenases acyltransferase family protein	0.66	0.00	0.03
gi|241946447	AT5G67090 - Subtilisin-like serine endopeptidase family protein	0.66	0.01	0.05
gi|241939408	AT5G10560 - putative beta-D-xylosidase 6	0.66	0.00	0.01
gi|241925636	AT2G25800 - uncharacterized protein	0.66	0.00	0.04
gi|241917638	RFNR2 - root FNR 2; Maintains the supply of reduced ferredoxin under non- photosynthetic conditions	0.65	0.00	0.03
gi|241944988	RBP-DR1 - RNA-binding protein-defense related 1	0.65	0.00	0.02
gi|241920768	AT5G15180 - peroxidase 56; Removal of H(2)O(2), oxidation of toxic reductants, biosynthesis and degradation of lignin, suberization, auxin catabolism, response to environmental stresses such as wounding, pathogen attack and oxidative stress. These functions	0.65	0.00	0.02

	might be dependent on each isozyme/isoform in each plant tissue			
gi|241925946	BGLU17 - beta glucosidase 17	0.65	0.01	0.05
gi|241937941	AT4G06744 - Leucine-rich repeat-containing protein	0.65	0.00	0.02
gi|241922455	AT3G48950 - glycoside hydrolase family 28 protein / polygalacturonase (pectinase) family protein	0.65	0.01	0.05
gi|241923284	PRMT3 - protein arginine methyltransferase 3; Methylates (mono and asymmetric dimethylation) the guanidino nitrogens of arginyl residues in some proteins (By similarity)	0.65	0.02	0.07
gi|241942846	CRK10 - cysteine-rich receptor-like protein kinase 10	0.65	0.01	0.07
gi|241935782	AT5G42510 - disease resistance-responsive, dirigent domain-containing protein	0.65	0.00	0.03
gi|241919092	DSEL - lipase class 3 family protein; Acylhydrolase that catalyzes the hydrolysis of 1,3- diacylglycerol (1,3-DAG) and 1-monoacylglycerol (1-MAG) at the sn- 1 position. High activity toward 1,3-DAG and 1-MAG, but low activity toward 1,2-diacylglycerol (1,2-DAG) and 1-lysophosphatidylcholine (1-LPC), and no activity toward phosphatidylcholine (PC), monogalactosyldiacylglycerol (MGDG), digalactosyldiacylglycerol (DGDG), triacylglycerol (TAG) and 2- monoacylglycerol (2-MAG). May be involved in the negative regulation of seedling establishment by inhibiting the breakdown, beta-oxidation and mobi [...]	0.65	0.00	0.03
gi|241924882	SYP131 - syntaxin 1B/2/3; Vesicle trafficking protein that functions in the secretory pathway (By similarity)	0.65	0.00	0.04
gi|241942126	AT3G59350 - protein kinase family protein	0.65	0.00	0.02
gi|241918012	SULTR1;3 - sulfate transporter 1.3; High-affinity H(+)/sulfate cotransporter that mediates the loading of sulfate into the sieve tube. Plays a central role in the regulation of sulfate assimilation	0.65	0.00	0.01

gi|241922807	PKP-ALPHA - Pyruvate kinase family protein; Required for plastidial pyruvate kinase activity. Involved in seed oil accumulation, embryo development and seed storage compounds mobilization upon germination	0.64	0.00	0.02
gi|241917616	AAO2 - aldehyde oxidase 2; In higher plant aldehyde oxidases (AO) appear to be homo- and heterodimeric assemblies of AO subunits with probably different physiological functions. In vitro, AO-gamma uses heptaldehyde, benzaldehyde, naphthaldehyde and cinnamaldehyde as substrates; AO-beta uses indole-3-acetaldehyde (IAAld), indole-3- aldehyde (IAld) and naphtaldehyde; the AAO2-AAO3 dimer uses abscisic aldehyde	0.64	0.01	0.05
gi|241942922	DiT1 - dicarboxylate transporter 1; 2-oxoglutarate/malate translocator involved with DIT2-1 in primary ammonia assimilation and in the re-assimilation of ammonia generated by the photorespiratory pathway. Imports 2- oxoglutarate into plastids as precursor for ammonia assimilation. 2-oxoglutarate is converted to glutamate, the end product of ammonia assimilation, which is exported to the cytosol by DIT2-1	0.64	0.00	0.04
gi|241924898	AT4G16720 - 60S ribosomal protein L15-1	0.64	0.02	0.07
gi|241925052	CEV1 - CONSTITUTIVE EXPRESSION OF VSP 1; Catalytic subunit of cellulose synthase terminal complexes ('rosettes'), required for beta-1,4-glucan microfibril crystallization, a major mechanism of the cell wall formation. Involved in the primary cell wall formation, especially in roots	0.64	0.00	0.02
gi|397912419	OMT1 - O-methyltransferase 1; Methylates OH residues of flavonoid compounds. Converts quercetin into isorhamnetin. Dihydroquercetin is not a substrate. Catalyzes the methylation of monolignols, the lignin precursors. Does not contribute to the phenylpropanoid	0.64	0.01	0.06

	pattern of the pollen tryphine, but is probably confined to isorhamnetin gylcoside biosynthesis			
gi|241933833	CLC-C - chloride channel C; Voltage-gated chloride channel	0.64	0.00	0.03
gi|241923204	ADT6 - arogenate dehydratase 6; Converts the prephenate produced from the shikimate- chorismate pathway into phenylalanine	0.64	0.00	0.03
gi|241914844	CNGC17 - cyclic nucleotide gated channel; Probable cyclic nucleotide-gated ion channel	0.64	0.02	0.07
gi|241934204	AT5G10770 - aspartyl protease family protein	0.63	0.00	0.04
gi|241927623	AT4G33420 - peroxidase; Removal of H(2)O(2), oxidation of toxic reductants, biosynthesis and degradation of lignin, suberization, auxin catabolism, response to environmental stresses such as wounding, pathogen attack and oxidative stress. These functions might be dependent on each isozyme/isoform in each plant tissue	0.63	0.01	0.05
gi|241919060	MTO1 - cystathionine gamma-synthase; Catalyzes the formation of L-cystathionine from O- succinyl-L-homoserine (OSHS) and L-cysteine, via a gamma-replacement reaction. In the absence of thiol, catalyzes gamma- elimination to form 2-oxobutanoate, succinate and ammonia (By similarity)	0.63	0.00	0.03
gi|241915637	VSR6 - VACUOLAR SORTING RECEPTOR 6; Vacuolar-sorting receptor (VSR) involved in clathrin-coated vesicles sorting from Golgi apparatus to vacuoles (By similarity)	0.63	0.02	0.07
gi|241931607	AOS - allene oxide synthase	0.63	0.00	0.03
gi|300078720	AVP1 - Pyrophosphate-energized vacuolar membrane proton pump 1; Contributes to the transtonoplast (from cytosol to vacuole lumen) H(+)-electrochemical potential difference. It establishes a proton gradient of similar and often greater magnitude than the H(+)-ATPase on the	0.63	0.01	0.05

	same membrane. In addition, facilitates auxin transport by modulating apoplastic pH and regulates auxin-mediated developmental processes. Confers tolerance to NaCl and to drought by increasing ion retention			
gi|241932372	AT1G22180 - sec.4-like phosphatidylinositol transfer protein	0.63	0.01	0.05
gi|241918021	AT3G44150 - uncharacterized protein	0.62	0.01	0.05
gi|241946332	AT2G41770 - uncharacterized protein	0.62	0.01	0.04
gi|241939240	AT1G65870 - Disease resistance-responsive (dirigent-like protein) family protein	0.62	0.00	0.03
gi|241916232	KASI - 3-ketoacyl-acyl carrier protein synthase I; Catalyzes the condensation reaction of fatty acid synthesis by the addition to an acyl acceptor of two carbons from malonyl-ACP. Specific for elongation from C-10 to unsaturated C-16 and C-18 fatty acids (By similarity)	0.62	0.00	0.01
gi|241940566	AT3G03773 - HSP20-like chaperon-like protein	0.62	0.01	0.05
gi|241938452	RRP4 - ribosomal RNA processing 4	0.62	0.00	0.03
gi|241927477	BXL3 - beta-xylosidase 3; Involved in the hydrolysis of arabinan. Can hydrolyze (1,3)-alpha-, (1,2)-alpha-linked side group residues and non- reducing terminal L-arabinofuranose residues of debranched (1,5)- alpha-L-arabinan backbone. Acts also as a beta-D-xylosidase, releasing D-xylose from arabinoxylan and xylan	0.62	0.00	0.03
gi|241916010	MUR1 - MURUS 1; Catalyzes the conversion of GDP-D-mannose to GDP-4- dehydro-6-deoxy-D-mannose	0.62	0.00	0.03
gi|241918942	KAI2 - KARRIKIN INSENSITIVE 2; Involved in seed germination and seedling development. Essential for plant responses to karrikins, a class of butenolide compounds, structurally similar to strigolactones, released from burning vegetation that stimulate seed germination and enhance seedling photomorphogenesis. KAI2 is not required for	0.62	0.00	0.03

	strigolactone-mediated responses, but MAX2 is necessary for responses to karrikins and strigolactones			
gi|241924477	AT4G33420 - peroxidase; Removal of H(2)O(2), oxidation of toxic reductants, biosynthesis and degradation of lignin, suberization, auxin catabolism, response to environmental stresses such as wounding, pathogen attack and oxidative stress. These functions might be dependent on each isozyme/isoform in each plant tissue	0.61	0.00	0.03
gi|241944467	AT1G19715 - mannose-binding lectin-like protein	0.61	0.00	0.02
gi|241919262	EMB3147 - EMBRYO DEFECTIVE 3147	0.61	0.00	0.02
gi|241935378	RWP1 - REDUCED LEVELS OF WALL-BOUND PHENOLICS 1; Involved in the synthesis of aromatics of the suberin polymer. Specifically affects the accumulation of the ferulate constituent of suberin in roots and seeds, but has no effect on the content of p-coumarate or sinapate	0.61	0.00	0.03
gi|241947360	LOS1 - elongation factor EF-2	0.61	0.01	0.07
gi|241917078	sks4 - SKU5 similar 4	0.61	0.01	0.05
gi|241920962	NUC-L2 - nucleolin; Involved in pre-rRNA processing and ribosome assembly (By similarity)	0.61	0.01	0.06
gi|241917485	TT5 - TRANSPARENT TESTA 5; Catalyzes the intramolecular cyclization of bicyclic chalcones into tricyclic (S)-flavanones. Responsible for the isomerization of 4,2',4',6'-tetrahydroxychalcone (also termed chalcone) into naringenin	0.61	0.01	0.05
gi|241917725	AT5G22020 - strictosidine synthase family protein	0.60	0.00	0.03
gi|241946582	CAM2 - calmodulin 2	0.60	0.01	0.05
gi|241945925	LPD1 - lipoamide dehydrogenase 1	0.60	0.00	0.02
gi|241920569	TUB8 - tubulin beta 8; Tubulin is the major constituent of microtubules. It binds two moles of GTP, one at an exchangeable site on the beta chain and one at a non-exchangeable site on the alpha chain	0.60	0.00	0.02

gi|241919543	ARLA1C - ADP-ribosylation factor-like A1C	0.60	0.00	0.03
gi|241930931	HDA3 - histone deacetylase 3; Probably mediates the deacetylation of lysine residues on the N-terminal part of the core histones (H2A, H2B, H3 and H4). Histone deacetylation gives a tag for epigenetic repression and plays an important role in transcriptional regulation, cell cycle progression and developmental events. Required for histone H3 'Lys-9' deacetylation. Involved in rRNA gene silencing in nucleolar dominance. Seems to be implicated in the regulation of genes involved in seeds development	0.60	0.00	0.03
gi|241928541	LAC12 - laccase 12; Lignin degradation and detoxification of lignin-derived products (By similarity)	0.60	0.00	0.02
gi|241918315	CYP86B1 - cytochrome P450 86B1; Involved in very long chain fatty acids (VLCFA) omega- hydroxylation. Required for the synthesis of saturated VLCFA alpha, omega-bifunctional suberin monomers	0.60	0.00	0.04
gi|241929393	HCT - hydroxycinnamoyl-CoA shikimate/quinate hydroxycinnamoyl transferase; Acyltransferase involved in the biosynthesis of lignin. Accepts caffeoyl-CoA and p-coumaroyl-CoA as substrates and transfers the acyl group on both shikimate and quinate acceptors	0.60	0.01	0.04
gi|241920407	GAMMA-H2AX - gamma histone variant H2AX; Variant histone H2A which replaces conventional H2A in a subset of nucleosomes. Nucleosomes wrap and compact DNA into chromatin, limiting DNA accessibility to the cellular machineries which require DNA as a template. Histones thereby play a central role in transcription regulation, DNA repair, DNA replication and chromosomal stability. DNA accessibility is regulated via a complex set of post-translational modifications of histones, also called histone code, and	0.60	0.00	0.03

	nucleosome remodeling. Required for checkpoint-mediated arrest of cell cycle progression in respon [...]			
gi|241921476	GSTU18 - glutathione S-transferase TAU 18; May be involved in the conjugation of reduced glutathione to a wide number of exogenous and endogenous hydrophobic electrophiles and have a detoxification role against certain herbicides (By similarity)	0.60	0.02	0.08
gi|241942542	GLDP2 - glycine decarboxylase P-protein 2; The glycine decarboxylase (GDC) or glycine cleavage system catalyzes the degradation of glycine. The P protein binds the alpha-amino group of glycine through its pyridoxal phosphate cofactor; CO(2) is released and the remaining methylamine moiety is then transferred to the lipoamide cofactor of the H protein (By similarity)	0.60	0.00	0.03
gi|241940146	LHT1 - Lysine histidine transporter 1; Amino acid-proton symporter. Transporter with a broad specificity for histidine, lysine, glutamic acid, alanine, serine, proline and glycine. Involved in both apoplastic transport of amino acids in leaves and their uptake by roots	0.60	0.01	0.07
gi|241926569	PLA2A - phospholipase A 2A	0.59	0.00	0.02
gi|241933762	ATC - centroradialis; May form complexes with phosphorylated ligands by interfering with kinases and their effectors (By similarity). Can substitute for TERMINAL FLOWER 1 (in vitro)	0.59	0.01	0.06
gi|241920389	TUA6 - Tubulin alpha-6; Tubulin is the major constituent of microtubules. It binds two moles of GTP, one at an exchangeable site on the beta chain and one at a non-exchangeable site on the alpha chain (By similarity)	0.59	0.01	0.04
gi|241937749	AT2G03200 - aspartyl protease-like protein	0.59	0.01	0.07
gi|21326129	UGD2 - UDP-glucose dehydrogenase 2; Involved in the biosynthesis of UDP-glucuronic acid (UDP-GlcA), providing nucleotide sugars for cell-wall polymers.	0.59	0.01	0.05

	Required for the formation of cell wall ingrowths on the outer cell walls of nematode-induced syncytia			
gi|144583703	AT4G35160 - O-methyltransferase family 2 protein	0.59	0.01	0.04
gi|241944347	BLH6 - BEL1-like homeodomain 6	0.59	0.01	0.05
gi|241933765	AT2G27730 - copper ion binding protein	0.58	0.00	0.04
gi|241945532	FLA11 - FASCICLIN-like arabinogalactan-protein 11; May be a cell surface adhesion protein	0.58	0.01	0.05
gi|241919320	AT5G48540 - cysteine-rich repeat secretory protein 55	0.58	0.01	0.04
gi|241933634	AT1G48090 - calcium-dependent lipid-binding-like protein	0.58	0.00	0.03
gi|241945347	PDCB3 - plasmodesmata callose-binding protein 3	0.58	0.00	0.02
gi|241916757	PRX52 - peroxidase 52; Removal of H(2)O(2), oxidation of toxic reductants, biosynthesis and degradation of lignin, suberization, auxin catabolism, response to environmental stresses such as wounding, pathogen attack and oxidative stress. These functions might be dependent on each isozyme/isoform in each plant tissue	0.58	0.00	0.03
gi|241928144	IPT1 - isopentenyltransferase 1; Involved in cytokinin biosynthesis. Catalyzes the transfer of an isopentenyl group from dimethylallyl diphosphate (DMAPP) to ATP, ADP and AMP. Adenine, adenosine, isopentenylpyrophosphate and 1-hydroxy-2-methyl-2-(E)-butenyl 4- diphosphate (HMBDP) are not used as substrates	0.58	0.04	0.12
gi|241915496	AT5G10770 - aspartyl protease family protein	0.58	0.00	0.03
gi|241919390	BGAL8 - beta-galactosidase 8	0.58	0.00	0.02
gi|6561805	UGT85A3 - UDP-glucosyl transferase 85A3	0.58	0.00	0.04
gi|241920467	LOX5 - lipoxygenase 5; 9S-lipoxygenase that can use linoleic acid or linolenic acid as substrates. Plant lipoxygenases may be involved in a number of diverse aspects of plant physiology including growth and development, pest resistance, and senescence or responses to wounding. Catalyzes the	0.58	0.00	0.01

	hydroperoxidation of lipids containing a cis,cis-1,4-pentadiene structure. Function as regulators of root development by controlling the emergence of lateral roots			
gi|241922275	AGT2 - alanine:glyoxylate aminotransferase 2	0.58	0.00	0.02
gi|241942301	COX15 - cytochrome c oxidase 15; May be involved in the biosynthesis of heme A (By similarity)	0.57	0.01	0.05
gi|241920096	ADPG1 - Polygalacturonase ADPG1; Polygalacturonase involved in cell separation in the final stages of pod shatter and in anther dehiscence. Not involved in floral organ abscission	0.57	0.01	0.07
gi|30090026	AT5G60920.1 - COBRA; Involved in determining the orientation of cell expansion, probably by playing an important role in cellulose deposition. May act by recruiting cellulose synthesizing complexes to discrete positions on the cell surface	0.57	0.00	0.03
gi|241933653	FBA6 - fructose-bisphosphate aldolase 6	0.57	0.04	0.11
gi|241931437	TUB8 - tubulin beta 8; Tubulin is the major constituent of microtubules. It binds two moles of GTP, one at an exchangeable site on the beta chain and one at a non-exchangeable site on the alpha chain	0.57	0.03	0.10
gi|241922542	TUB2 - tubulin beta chain 2; Tubulin is the major constituent of microtubules. It binds two moles of GTP, one at an exchangeable site on the beta chain and one at a non-exchangeable site on the alpha chain	0.57	0.01	0.05
gi|241937361	ACC1 - acetyl-CoA carboxylase 1; Multifunctional enzyme that catalyzes the carboxylation of acetyl-CoA, forming malonyl-CoA, which is used in the plastid for fatty acid synthesis and in the cytosol in various biosynthetic pathways including fatty acid elongation. Required for very long chain fatty acids elongation. Necessary for embryo and plant development. Plays a central function in embryo morphogenesis, especially in	0.57	0.00	0.02

	apical meristem development. Involved in cell proliferation and tissue patterning. May act as a repressor of cytokinin response			
gi|241930833	LigB - 4,5-DOPA dioxygenase extradiol-like protein; May be involved in the metabolism of aromatic compounds	0.57	0.00	0.04
gi|241933016	UGP2 - UDP-glucose pyrophosphorylase 2; Plays a central role as a glucosyl donor in cellular metabolic pathways (By similarity)	0.57	0.00	0.04
gi|241946020	HXK1 - hexokinase 1; Fructose and glucose phosphorylating enzyme. May be involved in the phosphorylation of glucose during the export from mitochondrion to cytosol. Acts as sugar sensor which may regulate sugar-dependent gene repression or activation. Mediates the effects of sugar on plant growth and development independently of its catalytic activity or the sugar metabolism. May regulate the execution of program cell death in plant cells	0.57	0.00	0.03
gi|241923082	AO4 - aldehyde oxidase 4; Involved in the accumulation of benzoic acid (BA) in siliques	0.57	0.00	0.04
gi|241938333	AT1G22480 - plastocyanin-like domain-containing protein	0.57	0.00	0.03
gi|241933962	HTA11 - histone H2A; Variant histone H2A which may replace conventional H2A in a subset of nucleosomes. Nucleosomes wrap and compact DNA into chromatin, limiting DNA accessibility to the cellular machineries which require DNA as a template. Histones thereby play a central role in transcription regulation, DNA repair, DNA replication and chromosomal stability. DNA accessibility is regulated via a complex set of post-translational modifications of histones, also called histone code, and nucleosome remodeling (By similarity)	0.57	0.00	0.02

gi|241939966	AT4G27450 - aluminum induced protein with YGL and LRDR motifs	0.56	0.00	0.04
gi|241916766	AT5G44400 - FAD-binding and BBE domain-containing protein	0.56	0.00	0.02
gi|241922866	HA11 - H(+)-ATPase 11; The plasma membrane H(+) ATPase of plants and fungi generates a proton gradient that drives the active transport of nutrients by H(+)-symport. The resulting external acidification and/or internal alkinization may mediate growth responses (By similarity)	0.56	0.00	0.01
gi|2766448	CYP98A3 - cytochrome P450 98A3; Cytochrome P450 which catalyzes 3'-hydroxylation of p- coumaric esters of shikimic/quinic acids to form lignin monomers. Can use p-coumarate, p-coumaraldehyde, p-coumaroyl methyl ester, 5-O-(4-coumaroyl) D-quinate and 5-O-(4-coumaroyl) shikimate as substrates, but not p-coumaryl alcohol, p-coumaroyl CoA, 1-O-p- coumaroyl-beta-D-glucose, p-hydroxy-cinnamyl alcohol, cinnamate, caffeate or ferulate. Has a weak activity on tri(p-coumaroyl)spermidine, but none on triferuloylspermidine. Hydroxylates preferentially the 5-O-isomer, but can also convert the 4-O- and 3-O-i [...]	0.56	0.01	0.05
gi|241928395	AT5G04885 - Glycosyl hydrolase family protein	0.56	0.00	0.01
gi|241919248	AT1G09160 - putative protein phosphatase 2C 5	0.56	0.01	0.05
gi|241930598	PDIL5-3 - PDI-like 5-3; Acts as a protein-folding catalyst that interacts with nascent polypeptides to catalyze the formation, isomerization, and reduction or oxidation of disulfide bonds (By similarity)	0.55	0.00	0.03
gi|241925966	CRL1 - CCR(Cinnamoyl coA:NADP oxidoreductase)-like 1	0.55	0.01	0.06
gi|241933673	AT5G42500 - disease resistance-responsive, dirigent domain-containing protein	0.55	0.00	0.03
gi|241933166	EHD1 - EPS15 homology domain 1	0.55	0.01	0.05

gi|241943311	HA11 - H(+)-ATPase 11; The plasma membrane H(+) ATPase of plants and fungi generates a proton gradient that drives the active transport of nutrients by H(+)-symport. The resulting external acidification and/or internal alkinization may mediate growth responses (By similarity)	0.55	0.00	0.01
gi|241920539	RXF12 - glycosyl hydrolase-like prottein 10	0.55	0.00	0.02
gi|241935866	ACLA-3 - ATP-citrate lyase A-3; ATP citrate-lyase is the primary enzyme responsible for the synthesis of cytosolic acetyl-CoA, used for the elongation of fatty acids and biosynthesis of isoprenoids, flavonoids and malonated derivatives. May supply substrate to the cytosolic acetyl-CoA carboxylase, which generates the malonyl-CoA used for the synthesis of a multitude of compounds, including very long chain fatty acids and flavonoids. Required for normal growth and development and elongation of C18 fatty acids to C20 to C24 fatty acids in seeds. In contrast to all known animal ACL enzymes having [...]	0.54	0.00	0.03
gi|241918822	AT5G46900 - bifunctional inhibitor/lipid-transfer protein/seed storage 2S albumin-like protein	0.54	0.02	0.08
gi|241944733	AT5G45910 - GDSL esterase/lipase	0.54	0.01	0.07
gi|241940007	AT1G71695 - peroxidase 12; Removal of H(2)O(2), oxidation of toxic reductants, biosynthesis and degradation of lignin, suberization, auxin catabolism, response to environmental stresses such as wounding, pathogen attack and oxidative stress. These functions might be dependent on each isozyme/isoform in each plant tissue	0.54	0.00	0.04
gi|241945533	FLA6 - FASCICLIN-like arabinogalactan 6; May be a cell surface adhesion protein	0.53	0.01	0.04
gi|241945053	AT1G03220 - aspartyl protease-like protein	0.53	0.00	0.04

gi|241928478	TUB6 - beta-6 tubulin; Tubulin is the major constituent of microtubules. It binds two moles of GTP, one at an exchangeable site on the beta chain and one at a non-exchangeable site on the alpha chain (By similarity)	0.53	0.00	0.03
gi|241936880	NPC4 - phospholipase C	0.53	0.01	0.04
gi|241939702	GlcNA.1UT1 - N-acetylglucosamine-1-phosphate uridylyltransferase 1; Uridylyltransferase involved in the biosynthesis of UDP- glucosamine, an essential precursor for glycoprotein and glycolipid synthesis. Can use both UDP-glucosamine and the 4- epimer UDP-galactosamine as substrates, but no other sugars or NTPs	0.53	0.00	0.03
gi|241916753	PRX52 - peroxidase 52; Removal of H(2)O(2), oxidation of toxic reductants, biosynthesis and degradation of lignin, suberization, auxin catabolism, response to environmental stresses such as wounding, pathogen attack and oxidative stress. These functions might be dependent on each isozyme/isoform in each plant tissue	0.53	0.00	0.04
gi|241926030	AT5G47530 - putative auxin-responsive protein	0.53	0.01	0.05
gi|241941045	AT4G37530 - peroxidase 51; Removal of H(2)O(2), oxidation of toxic reductants, biosynthesis and degradation of lignin, suberization, auxin catabolism, response to environmental stresses such as wounding, pathogen attack and oxidative stress. These functions might be dependent on each isozyme/isoform in each plant tissue	0.53	0.00	0.02
gi|241922103	RKL1 - receptor-like kinase 1	0.53	0.00	0.03
gi|241931677	PRX52 - peroxidase 52; Removal of H(2)O(2), oxidation of toxic reductants, biosynthesis and degradation of lignin, suberization, auxin catabolism, response to environmental stresses such as wounding, pathogen attack and oxidative stress. These functions might be dependent on each isozyme/isoform in each plant tissue	0.53	0.00	0.01

gi|241945942	FLA2 - FASCICLIN-like arabinogalactan 2; May be a cell surface adhesion protein	0.53	0.00	0.04
gi|241917606	AT3G22600 - bifunctional inhibitor/lipid-transfer protein/seed storage 2S albumin-like protein	0.52	0.00	0.03
gi|241941580	AT2G22230 - 3R-hydroxymyristoyl ACP dehydrase	0.52	0.00	0.03
gi|241930877	AT3G09410 - putative pectinacetylesterase	0.52	0.01	0.06
gi|241921420	SSL4 - strictosidine synthase-like 4	0.52	0.00	0.04
gi|241932535	4CL3 - 4-coumarate:CoA ligase 3; Produces CoA thioesters of a variety of hydroxy- and methoxy-substituted cinnamic acids, which are used to synthesize several phenylpropanoid-derived compounds, including anthocyanins, flavonoids, isoflavonoids, coumarins, lignin, suberin and wall-bound phenolics	0.52	0.01	0.05
gi|241923083	AAO3 - abscisic-aldehyde oxidase; In higher plants aldehyde oxidases (AO) appear to be homo- and heterodimeric assemblies of AO subunits with probably different physiological functions. AO-delta seems to be involved in the last step of abscisic acid biosynthesis, at least in leaves and seeds. In vitro, AO-delta oxidizes abscisic aldehyde to abscisic acid (ABA). In vitro, AO-delta also uses indole-3- aldehyde (IAld), benzaldehyde, 1-naphthaldehyde and cinnamaldehyde as substrate; the AAO2-AAO3 dimer also uses abscisic aldehyde as substrate	0.52	0.00	0.01
gi|241923085	AAO2 - aldehyde oxidase 2; In higher plant aldehyde oxidases (AO) appear to be homo- and heterodimeric assemblies of AO subunits with probably different physiological functions. In vitro, AO-gamma uses heptaldehyde, benzaldehyde, naphthaldehyde and cinnamaldehyde as substrates; AO-beta uses indole-3-acetaldehyde (IAAld), indole-3- aldehyde (IAld) and	0.52	0.00	0.04

	naphtaldehyde; the AAO2-AAO3 dimer uses abscisic aldehyde			
gi|241943857	AT3G63460 - protein transport protein SEC31	0.51	0.04	0.11
gi|241925772	AT2G19380 - RNA recognition motif (RRM)-containing protein	0.51	0.02	0.09
gi|241922547	EXPB2 - expansin B2; May cause loosening and extension of plant cell walls by disrupting non-covalent bonding between cellulose microfibrils and matrix glucans. No enzymatic activity has been found (By similarity)	0.51	0.01	0.04
gi|241947396	AT5G43310 - COP1-interacting protein-like protein	0.51	0.00	0.02
gi|241933084	AT5G20950 - Glycosyl hydrolase family protein	0.51	0.00	0.04
gi|241927471	AT4G33420 - peroxidase; Removal of H(2)O(2), oxidation of toxic reductants, biosynthesis and degradation of lignin, suberization, auxin catabolism, response to environmental stresses such as wounding, pathogen attack and oxidative stress. These functions might be dependent on each isozyme/isoform in each plant tissue	0.51	0.00	0.03
gi|241922723	AIR9 - AUXIN-INDUCED IN ROOT CULTURES 9	0.51	0.00	0.02
gi|241921469	ERD9 - glutathione S-transferase; Involved in light signaling, mainly phyA-mediated photomorphogenesis and in the integration of various phytohormone signals to modulate various aspects of plant development by affecting glutathione pools. In vitro, possesses glutathione S- transferase activity toward 1-chloro-2,4-dinitrobenzene (CDNB) and benzyl isothiocyanate (BITC)	0.50	0.00	0.02
gi|241938524	AT1G63220 - calcium-dependent lipid-binding domain-containing protein	0.50	0.00	0.03
gi|241941168	CCoAOMT1 - caffeoyl coenzyme A O-methyltransferase 1; Methylates caffeoyl-CoA to feruloyl-CoA. Has a very	0.50	0.01	0.06

	low activity with caffeic acid and esculetin. Involved in scopoletin biosynthesis in roots			
gi|241927409	AT2G39040 - peroxidase 24; Removal of H(2)O(2), oxidation of toxic reductants, biosynthesis and degradation of lignin, suberization, auxin catabolism, response to environmental stresses such as wounding, pathogen attack and oxidative stress. These functions might be dependent on each isozyme/isoform in each plant tissue	0.50	0.00	0.04
gi|241937192	AT2G46890 - uncharacterized protein	0.50	0.01	0.06
gi|241928695	AT4G09160 - patellin-5; Carrier protein that may be involved in membrane- trafficking events associated with cell plate formation during cytokinesis. Binds to some hydrophobic molecules such as phosphoinositides and promotes their transfer between the different cellular sites (By similarity)	0.50	0.00	0.02
gi|241926133	AT1G76010 - Alba DNA/RNA-binding protein	0.49	0.00	0.04
gi|241937790	AT5G15140 - aldose 1-epimerase	0.49	0.01	0.05
gi|241923991	LYM2 - lysM domain GPI-anchored protein 2 precursor; Chitin elicitor-binding protein involved in the perception of chitin oligosaccharide elicitor	0.48	0.01	0.05
gi|267026650	BFRUCT4 - beta-fructofuranosidase; Possible role in the continued mobilization of sucrose to sink organs. Regulates root elongation	0.48	0.01	0.05
gi|241924683	AT5G45490 - putative disease resistance protein; Possible disease resistance protein (By similarity)	0.48	0.00	0.04
gi|241922750	AT5G36160 - tyrosine aminotransferase; Transaminase involved in tyrosine breakdown. Converts tyrosine to p-hydroxyphenylpyruvate. Can catalyze the reverse reaction, using L-glutamate in vitro. Can convert phenylalanine to phenylpyruvate and catalyze the reverse reaction in vitro	0.48	0.00	0.03

gi|241941549	PAO1 - Polyamine oxidase 1; Flavoenzyme that catalyzes the oxidation of the secondary amino group of spermine, norspermine and N(1)- acetylspermine. Substrate preference is norspermine > spermine > N(1)-acetylspermine. No activity detected when putrescine, spermidine or N(1)-acetylspermidine are used as substrates. Plays an important role in the regulation of polyamine intracellular concentration	0.48	0.00	0.02
gi|241940766	ALDH11A3 - aldehyde dehydrogenase 11A3; Important as a means of generating NADPH for biosynthetic reactions	0.48	0.01	0.06
gi|241925983	AT2G24580 - putative sarcosine oxidase	0.47	0.00	0.03
gi|241921012	PHT1;7 - phosphate transporter 1;7; High-affinity transporter for external inorganic phosphate (By similarity)	0.47	0.00	0.04
gi|241945305	RCI3 - peroxidase 3; Removal of H(2)O(2), oxidation of toxic reductants, biosynthesis and degradation of lignin, suberization, auxin catabolism, response to environmental stresses such as wounding, pathogen attack and oxidative stress. These functions might be dependent on each isozyme/isoform in each plant tissue	0.47	0.00	0.03
gi|241916339	AT1G18160 - protein kinase family protein	0.47	0.02	0.08
gi|241937843	AT2G43670 - carbohydrate-binding X8 domain-containing protein	0.47	0.03	0.09
gi|241923857	AIR12 - auxin-responsive-like protein	0.46	0.00	0.01
gi|241932482	AT1G76550 - pyrophosphate--fructose-6-phosphate 1-phosphotransferase; Regulatory subunit of pyrophosphate--fructose 6- phosphate 1-phosphotransferase (PFP) (By similarity)	0.46	0.00	0.03
gi|241918220	AT4G24130 - uncharacterized protein	0.45	0.00	0.03
gi|4680214	AT4G37900 - uncharacterized protein	0.45	0.01	0.05
gi|241930710	HTB9 - histone H2B; Core component of nucleosome. Nucleosomes wrap and compact DNA into chromatin,	0.45	0.01	0.06

	limiting DNA accessibility to the cellular machineries which require DNA as a template. Histones thereby play a central role in transcription regulation, DNA repair, DNA replication and chromosomal stability. DNA accessibility is regulated via a complex set of post-translational modifications of histones, also called histone code, and nucleosome remodeling (By similarity)			
gi|241932651	UCC1 - uclacyanin 1	0.44	0.01	0.05
gi|241919015	SUS4 - sucrose synthase 4; Sucrose-cleaving enzyme that provides UDP-glucose and fructose for various metabolic pathways (By similarity)	0.44	0.01	0.05
gi|241922599	ENODL20 - early nodulin-like protein 20	0.44	0.00	0.02
gi|241946491	AT2G39795 - glycoprotein-like protein	0.44	0.00	0.03
gi|241921304	AT1G27480 - Lecithin-cholesterol acyltransferase-like 1	0.43	0.00	0.04
gi|241945829	AT5G66390 - peroxidase 72; Removal of H(2)O(2), oxidation of toxic reductants, biosynthesis and degradation of lignin, suberization, auxin catabolism, response to environmental stresses such as wounding, pathogen attack and oxidative stress. These functions might be dependent on each isozyme/isoform in each plant tissue	0.43	0.00	0.03
gi|1658193	CYP51G1 - CYTOCHROME P450 51G1; Involved in sterol biosynthesis. Catalyzes the 14-alpha demethylation of obtusifoliol to 4 alpha-methyl-5 alpha-ergosta- 8,14,24(28)-trien-3 beta-ol	0.43	0.00	0.01
gi|241918972	PDCB3 - plasmodesmata callose-binding protein 3	0.43	0.00	0.03
gi|241938364	BFN1 - bifunctional nuclease i; Hydrolyzes only single stranded DNA and RNA without apparent specificity for bases during senescence. Endonuclease that recognizes and cleaves all types of mismatches with high efficiency, including heteroduplex double-stranded	0.43	0.01	0.05

	DNA. Maybe involved in programmed cell death (PCD) and senescence			
gi|241919878	CCT - CENTER CITY; Component of the Mediator complex, a coactivator involved in the regulated transcription of nearly all RNA polymerase II-dependent genes. Mediator functions as a bridge to convey information from gene-specific regulatory proteins to the basal RNA polymerase II transcription machinery. The Mediator complex, having a compact conformation in its free form, is recruited to promoters by direct interactions with regulatory proteins and serves for the assembly of a functional preinitiation complex with RNA polymerase II and the general transcription factors. Flowering regulator w [...]	0.43	0.01	0.06
gi|241920686	ARA12 - subtilisin-like protease; Serine protease. Has a substrate preference for the hydrophobic residues Phe and Ala and the basic residue Asp in the P1 position, and for Asp, Leu or Ala in the P1' position	0.42	0.00	0.02
gi|241933499	AT5G45910 - GDSL esterase/lipase	0.42	0.00	0.03
gi|241937714	ATFP8 - Ras-related protein RABD1; Protein transport. Regulator of membrane traffic from the Golgi apparatus towards the endoplasmic reticulum (ER)	0.42	0.01	0.07
gi|241924484	AT1G28600 - GDSL esterase/lipase	0.42	0.00	0.02
gi|241916084	AT5G45910 - GDSL esterase/lipase	0.42	0.00	0.03
gi|241931327	AT5G64420 - DNA polymerase phi subunit	0.42	0.00	0.03
gi|241923081	AAO1 - aldehyde oxidase 1; In higher plants aldehyde oxidases (AO) appear to be homo- and heterodimeric assemblies of AO subunits with probably different physiological functions. AO-alpha may be involved in the biosynthesis of auxin, and in biosynthesis of abscisic acid (ABA) in seeds. In vitro, AO-alpha uses heptaldehyde, protocatechualdehyde, benzaldehyde, indole-3-aldehyde (IAld), indole-3-acetaldehyde (IAAld),	0.42	0.00	0.03

	cinnamaldehyde and citral as substrates; AO-beta uses IAAld, IAld and naphtaldehyde as substrates			
gi|241929684	AT4G33420 - peroxidase; Removal of H(2)O(2), oxidation of toxic reductants, biosynthesis and degradation of lignin, suberization, auxin catabolism, response to environmental stresses such as wounding, pathogen attack and oxidative stress. These functions might be dependent on each isozyme/isoform in each plant tissue	0.42	0.01	0.06
gi|241945799	AT3G52250 - duplicated SANT DNA-binding domain-containing protein	0.41	0.00	0.03
gi|241918821	AT2G45180 - protease inhibitor/seed storage/lipid transfer protein (LTP) family protein	0.41	0.01	0.05
gi|241946466	FD3 - ferredoxin 3; Ferredoxins are iron-sulfur proteins that transfer electrons in a wide variety of metabolic reactions	0.41	0.00	0.02
gi|1127575	BGLU13 - beta glucosidase 13	0.40	0.00	0.03
gi|241935468	XTH5 - xyloglucan:xyloglucosyl transferase; Catalyzes xyloglucan endohydrolysis (XEH) and/or endotransglycosylation (XET). Cleaves and religates xyloglucan polymers, an essential constituent of the primary cell wall, and thereby participates in cell wall construction of growing tissues (By similarity)	0.40	0.00	0.04
gi|241938498	AT5G23110 - C3HC4-type RING finger domain-containing protein	0.40	0.01	0.05
gi|241938260	AT3G23840 - HXXXD-type acyl-transferase-like protein	0.39	0.01	0.06
gi|241940984	BAG1 - BCL-2-associated athanogene 1; Co-chaperone that regulates diverse cellular pathways, such as programmed cell death and stress responses (By similarity)	0.39	0.01	0.06
gi|241932905	AT3G53140 - putative O-diphenol-O-methyl transferase	0.36	0.03	0.09
gi|241918204	AT5G42510 - disease resistance-responsive, dirigent domain-containing protein	0.36	0.01	0.06

gi|241925490	FLA2 - FASCICLIN-like arabinogalactan 2; May be a cell surface adhesion protein	0.36	0.00	0.02
gi|241916764	AT4G20820 - FAD-binding and BBE domain-containing protein	0.34	0.00	0.03
gi|241921372	AT4G12510 - bifunctional inhibitor/lipid-transfer protein/seed storage 2S albumin-like protein	0.34	0.00	0.02
gi|241918803	EXPB2 - expansin B2; May cause loosening and extension of plant cell walls by disrupting non-covalent bonding between cellulose microfibrils and matrix glucans. No enzymatic activity has been found (By similarity)	0.34	0.00	0.03
gi|241929219	AT5G19950 - uncharacterized protein	0.34	0.02	0.09
gi|241944523	AT3G62160 - HXXXD-type acyl-transferase-like protein	0.34	0.00	0.02
gi|241946418	AT5G06730 - peroxidase 54; Removal of H(2)O(2), oxidation of toxic reductants, biosynthesis and degradation of lignin, suberization, auxin catabolism, response to environmental stresses such as wounding, pathogen attack and oxidative stress. These functions might be dependent on each isozyme/isoform in each plant tissue	0.33	0.00	0.02
gi|241942899	AT4G10500 - oxidoreductase, 2OG-Fe(II) oxygenase family protein	0.30	0.01	0.04
gi|241930797	RWP1 - REDUCED LEVELS OF WALL-BOUND PHENOLICS 1; Involved in the synthesis of aromatics of the suberin polymer. Specifically affects the accumulation of the ferulate constituent of suberin in roots and seeds, but has no effect on the content of p-coumarate or sinapate	0.29	0.00	0.01
gi|241944083	AT2G28790 - pathogenesis-related thaumatin-like protein	0.28	0.00	0.04
gi|241930262	FLA11 - FASCICLIN-like arabinogalactan-protein 11; May be a cell surface adhesion protein	0.28	0.00	0.02

gi|241938097	AT4G12510 - bifunctional inhibitor/lipid-transfer protein/seed storage 2S albumin-like protein	0.28	0.01	0.06
gi|241928307	PRF5 - profilin 5; Binds to actin and affects the structure of the cytoskeleton. At high concentrations, profilin prevents the polymerization of actin, whereas it enhances it at low concentrations. By binding to PIP2, it inhibits the formation of IP3 and DG (By similarity)	0.26	0.01	0.04
gi|241933544	PCAP1 - plasma-membrane associated cation-binding protein 1; May be involved in intracellular signaling through interaction with PtdInsPs and calmodulin (CaM); may keep PtdInsPs attached to the plasma membrane until Ca(2+)-CaM reaches a competitive concentration subsequent to an increase triggered by a stimulus, thus leading to PtdInsPs release and subsequent activation of InsPs-dependent signaling cascade. Interacts competitively at the N-terminus with calcium ions and CaM (in a calcium-dependent manner), and with the phosphatidylinositol phosphates PtdIns(3,4,5)P(3), PtdIns(3,4)P(2).	0.25	0.00	0.01

[1]Average fold change ratio defined as the average intensity of Al treated divided by the average intensity of the control. [2]The variance of the average fold change ration ($\boldsymbol{\sigma}^2$). [3]The standard error ($\boldsymbol{\sigma}/\sqrt{N}$). (For details of the statistical analysis associated with the data presented in this table the reader is referred to the subsection, ”Quantitative protein expression profiles in the sorghum root tip regions” of the Results section of the manuscript.) To convert the GI numbers in this table to the new Accession.Version identifiers use EFetch as described at: https://ncbiinsights.ncbi.nlm.nih.gov/2016/12/06/converting-gi-numbers-to-accession-version/”.

For details of the statistical analysis associated with the data presented in this table, the reader is referred to the subsection "Quantitative protein expression profiles in the sorghum root tip regions" of the Results section of the paper.
