# Supplementary material for: Quantitative iTRAQ Proteomics Revealed Possible Roles for Antioxidant Proteins in Sorghum Aluminum Tolerance
Source: Front Plant Sci. 2017 Jan 9;7:2043. doi: 10.3389/fpls.2016.02043 (PMC5220100; doi:10.3389/fpls.2016.02043)
Supplement: Table S3 — Differentially expressed proteins (fold changes) in 3D SC566. [file Table3.PDF]

Table S3. Differentially Expressed Proteins (Fold Change) in 3D SC566.

Protein ID	Protein Description	Relative Protein Expression[1]	Variance[2]	SE[3]
gi|613447702	XRN4 - exoribonuclease 4; Possesses 5'->3' exoribonuclease activity. Acts as an endogenous post-transcriptional gene silencing (PTGS) suppressor. Degrades miRNA target cleavage products that lack a 5'-cap structure. Antagonizes the negative feedback regulation on EIN3 by promoting EBF1 and EBF2 mRNA decay, which consequently allows the accumulation of EIN3 protein to trigger the ethylene response	6.79	10.99	1.91
gi|241947322	HCHIB - chitinase; Defense against chitin containing fungal pathogens. Seems particularly implicated in resistance to jasmonate-inducing pathogens such as A.brassicicola. In vitro antifungal activity against T.reesei, but not against A.solani, F.oxysporum, S.sclerotiorum, G.graminis and P.megasperma	5.24	2.45	0.90
gi|241917136	AT4G30380 - putative EG45-like domain-containing protein 1; Might have a systemic role in water and solute homeostasis (By similarity)	4.94	5.23	1.32
gi|241931049	BG3 - beta-1,3-glucanase 3	4.60	1.15	0.62
gi|241935663	ARPN - plantacyanin; Forms a concentration gradient along the pollen tube growth path, with a lower level in the stigma papilla cell wall and a higher level in the transmitting tract extracellular matix of the style	4.51	2.40	0.89
gi|241929418	LCR83 - putative defensin-like protein 70	3.98	0.39	0.36
gi|241914628	PR1 - pathogenesis-related gene 1; Partially responsible for acquired pathogen resistance	3.47	0.22	0.27
gi|241943277	OSM34 - osmotin 34	3.21	0.66	0.47
gi|241932472	AT5G10770 - aspartyl protease family protein	3.06	0.76	0.50

gi|241926454	OSM34 - osmotin 34	3.01	0.09	0.17
gi|241933008	CAT1 - catalase 1; Occurs in almost all aerobically respiring organisms and serves to protect cells from the toxic effects of hydrogen peroxide (By similarity)	2.73	0.09	0.18
gi|241920566	OSM34 - osmotin 34	2.68	0.32	0.33
gi|241943109	BGL2 - beta-1,3-glucanase 2; Implicated in the defense of plants against pathogens	2.56	0.26	0.30
gi|15529121	EP3 - chitinase	2.56	0.27	0.30
gi|241935615	PR4 - hevein-like protein; Fungal growth inhibitors. Neither CB-HEL nor CD-HEL have chitinase activity, but both have antimicrobial activities. CD-HEL has RNase, but no DNase activity	2.42	0.10	0.18
gi|241946897	BCS1 - cytochrome BC1 synthesis	2.40	0.91	0.55
gi|241935976	PRX52 - peroxidase 52; Removal of H(2)O(2), oxidation of toxic reductants, biosynthesis and degradation of lignin, suberization, auxin catabolism, response to environmental stresses such as wounding, pathogen attack and oxidative stress. These functions might be dependent on each isozyme/isoform in each plant tissue	2.33	0.02	0.08
gi|241931078	GSTU7 - glutathione S-transferase tau 7; May be involved in the conjugation of reduced glutathione to a wide number of exogenous and endogenous hydrophobic electrophiles and have a detoxification role against certain herbicides (By similarity)	2.25	0.21	0.26
gi|241927239	LPR1 - Low Phosphate Root1	2.22	0.17	0.24
gi|28569609	OMT1 - O-methyltransferase 1; Methylates OH residues of flavonoid compounds. Converts quercetin into isorhamnetin. Dihydroquercetin is not a substrate. Catalyzes the methylation of monolignols, the lignin precursors. Does not contribute to the phenylpropanoid pattern of the pollen tryphine, but is probably confined to isorhamnetin gylcoside biosynthesis	2.20	0.10	0.18

gi|241943470	LP1 - lipid transfer protein 1; Plant non-specific lipid-transfer proteins transfer phospholipids as well as galactolipids across membranes. May play a role in wax or cutin deposition in the cell walls of expanding epidermal cells and certain secretory tissues	2.18	0.13	0.21
gi|241923890	RH36 - RNA helicase 36	2.11	0.18	0.24
gi|241924929	CHIA - chitinase A	2.10	0.01	0.04
gi|241929783	AT3G05545 - RING/U-box domain-containing protein	2.10	0.03	0.10
gi|241924606	EULS3 - Euonymus lectin S3	2.04	0.06	0.14
gi|241921861	PYL6 - PYR1-like 6; Receptor for abscisic acid (ABA) required for ABA- mediated responses such as stomatal closure and germination inhibition. Inhibits the activity of group-A protein phosphatases type 2C (PP2Cs) when activated by ABA (By similarity)	2.02	0.06	0.14
gi|241940843	AT2G03200 - aspartyl protease-like protein	2.00	0.12	0.20
gi|241928915	GLP5 - germin-like protein 5; May play a role in plant defense. Probably has no oxalate oxidase activity even if the active site is conserved	2.00	0.18	0.25
gi|241942022	WAT1 - Walls Are Thin 1; Required for secondary wall formation in fibers, especially in short days conditions. Promotes indole metabolism and transport (e.g. tryptophan, neoglucobrassicin and auxin (indole-3-acetic acid)). May prevent salicylic-acid (SA) accumulation	1.99	0.02	0.09
gi|241930936	cICDH - isocitrate dehydrogenase; May supply 2-oxoglutarate for amino acid biosynthesis and ammonia assimilation via the glutamine synthetase/glutamate synthase (GS/GOGAT) pathway. May be involved in the production of NADPH to promote redox signaling or homeostasis in response to oxidative stress	1.98	0.07	0.16
gi|241920119	AATP1 - AAA-ATPase 1	1.97	0.01	0.07
gi|241922547	EXPB2 - expansin B2; May cause loosening and extension of plant cell walls by disrupting non-covalent	1.93	0.02	0.08

	bonding between cellulose microfibrils and matrix glucans. No enzymatic activity has been found (By similarity)			
gi|241935376	AT4G32110 - beta-1,3-N-Acetylglucosaminyltransferase family protein	1.93	0.04	0.12
gi|241925991	AT3G25290 - putative auxin-responsive protein	1.92	0.16	0.23
gi|241925000	AT3G28510 - AAA-type ATPase family protein	1.91	0.03	0.10
gi|241926425	FLR1 - FLOR1	1.90	0.04	0.11
gi|241934439	FLS1 - flavonol synthase 1; Catalyzes the formation of flavonols from dihydroflavonols. It can act on dihydrokaempferol to produce kaempferol, on dihydroquercetin to produce quercitin and on dihydromyricetin to produce myricetin. In vitro catalyzes the oxidation of both enantiomers of naringenin to give both cis- and trans-dihydrokaempferol	1.89	0.05	0.13
gi|241938127	XRN4 - exoribonuclease 4; Possesses 5'->3' exoribonuclease activity. Acts as an endogenous post-transcriptional gene silencing (PTGS) suppressor. Degrades miRNA target cleavage products that lack a 5'-cap structure. Antagonizes the negative feedback regulation on EIN3 by promoting EBF1 and EBF2 mRNA decay, which consequently allows the accumulation of EIN3 protein to trigger the ethylene response	1.87	0.08	0.16
gi|58978027	PYL12 - PYR1-like 12; Receptor for abscisic acid (ABA) required for ABA- mediated responses such as stomatal closure and germination inhibition. Inhibits the activity of group-A protein phosphatases type 2C (PP2Cs) when activated by ABA (By similarity)	1.86	0.17	0.24
gi|241921441	DMR6 - DOWNY MILDEW RESISTANT 6	1.85	0.08	0.16
gi|241926637	PA2 - peroxidase 2; Removal of H(2)O(2), oxidation of toxic reductants, biosynthesis and degradation of lignin, suberization, auxin catabolism, response to	1.81	0.01	0.07

	environmental stresses such as wounding, pathogen attack and oxidative stress. These functions might be dependent on each isozyme/isoform in each plant tissue			
gi|241930937	PEX6 - peroxin 6; Involved in peroxisomal-targeting signal one (PTS1) and peroxisomal-targeting signal two (PTS2) protein import. Required for jasmonate biosynthesis. Necessary for the developmental elimination of obsolete peroxisome matix proteins. May form heteromeric AAA ATPase complexes required for the import of proteins. May be involved in PEX5 recycling	1.81	0.02	0.09
gi|241929566	FPF1 - FLOWERING PROMOTING FACTOR 1; Modulates the competence to flowering of apical meristems. Involved in a GA-dependent response in apical meristems during the transition to flowering	1.80	0.02	0.09
gi|241922789	AT3G22600 - bifunctional inhibitor/lipid-transfer protein/seed storage 2S albumin-like protein	1.79	0.02	0.07
gi|241946869	scpl50 - serine carboxypeptidase-like 50; Probable carboxypeptidase (By similarity)	1.79	0.07	0.15
gi|241919699	PHT1;7 - phosphate transporter 1;7; High-affinity transporter for external inorganic phosphate (By similarity)	1.78	0.18	0.25
gi|241944382	PGIP1 - Polygalacturonase inhibitor 1; Inhibitor of fungal polygalacturonase. It is an important factor for plant resistance to phytopathogenic fungi	1.77	0.08	0.16
gi|119852231	ATCAD4 - cinnamyl alcohol dehydrogenase 4; Involved in lignin biosynthesis in the floral stem. Catalyzes the final step specific for the production of lignin monomers. Catalyzes the NADPH-dependent reduction of coniferaldehyde, 5-hydroxyconiferaldehyde, sinapaldehyde, 4- coumaraldehyde and caffeyl aldehyde to their respective alcohols	1.75	0.02	0.07

gi|241942135	AT5G61820 - uncharacterized protein	1.75	0.01	0.07
gi|241925267	PHT1;3 - phosphate transporter 1;3; High-affinity transporter for external inorganic phosphate (By similarity)	1.75	0.35	0.34
gi|241937783	PLAT1 - PLAT domain protein 1	1.73	0.04	0.11
gi|241921778	AT1G79620 - leucine-rich repeat protein kinase-like protein	1.72	0.08	0.16
gi|241931679	PRX52 - peroxidase 52; Removal of H(2)O(2), oxidation of toxic reductants, biosynthesis and degradation of lignin, suberization, auxin catabolism, response to environmental stresses such as wounding, pathogen attack and oxidative stress. These functions might be dependent on each isozyme/isoform in each plant tissue	1.71	0.03	0.09
gi|241938280	AGO7 - ARGONAUTE7; Involved in RNA-mediated post-transcriptional gene silencing (PTGS). Main component of the RNA-induced silencing complex (RISC) that binds to a short guide RNA such as a microRNA (miRNA) or small interfering RNA (siRNA). RISC uses the mature miRNA or siRNA as a guide for slicer-directed cleavage of homologous mRNAs to repress gene expression. Required for the processing of 21 nucleotide trans-acting siRNAs (ta-siRNAs) derived from TAS3a transcripts. Associates preferentially with the microRNA (miRNA) miR390 which guides the cleavage of TAS3 precursor RNA. Seems to act as mi [...]	1.71	0.01	0.07
gi|241917861	ARPN - plantacyanin; Forms a concentration gradient along the pollen tube growth path, with a lower level in the stigma papilla cell wall and a higher level in the transmitting tract extracellular matix of the style	1.70	0.01	0.07
gi|241932218	AT1G67900 - phototropic-responsive NPH3-like protein; May act as a substrate-specific adapter of an	1.69	0.19	0.25

	E3 ubiquitin-protein ligase complex (CUL3-RBX1-BTB) which mediates the ubiquitination and subsequent proteasomal degradation of target proteins (By similarity)			
gi|58978057	PYL12 - PYR1-like 12; Receptor for abscisic acid (ABA) required for ABA- mediated responses such as stomatal closure and germination inhibition. Inhibits the activity of group-A protein phosphatases type 2C (PP2Cs) when activated by ABA (By similarity)	1.69	0.02	0.09
gi|241921714	BMY3 - beta-amylase 3	1.68	0.10	0.18
gi|669030678	BGLU42 - beta glucosidase 42	1.67	0.03	0.10
gi|241918895	CYP704A2 - cytochrome P450, family 704, subfamily A, polypeptide 2	1.67	0.03	0.09
gi|241928058	CHIA - chitinase A	1.66	0.04	0.11
gi|241929173	PFK3 - phosphofructokinase 3	1.65	0.02	0.08
gi|241935975	PRX52 - peroxidase 52; Removal of H(2)O(2), oxidation of toxic reductants, biosynthesis and degradation of lignin, suberization, auxin catabolism, response to environmental stresses such as wounding, pathogen attack and oxidative stress. These functions might be dependent on each isozyme/isoform in each plant tissue	1.65	0.04	0.11
gi|241939008	AT1G01540 - putative serine/threonine-protein kinase	1.65	0.02	0.09
gi|241945724	UPI - UNUSUAL SERINE PROTEASE INHIBITOR	1.64	0.03	0.10
gi|241939376	PAL1 - PHE ammonia lyase 1; This is a key enzyme of plant metabolism catalyzing the first reaction in the biosynthesis from L-phenylalanine of a wide variety of natural products based on the phenylpropane skeleton	1.64	0.01	0.07
gi|15529117	EP3 - chitinase	1.63	0.03	0.09
gi|241916167	AT1G25420 - Regulator of Vps4 activity in the MVB pathway protein	1.63	0.14	0.21
gi|58977980	PYL6 - PYR1-like 6; Receptor for abscisic acid (ABA) required for ABA- mediated responses such as	1.62	0.01	0.07

	stomatal closure and germination inhibition. Inhibits the activity of group-A protein phosphatases type 2C (PP2Cs) when activated by ABA (By similarity)			
gi|241924078	PAP27 - purple acid phosphatase 27	1.62	0.00	0.04
gi|241926327	ICL - isocitrate lyase; Involved in storage lipid mobilization during the growth of higher plant seedling	1.61	0.02	0.08
gi|241915296	AT5G24165 - uncharacterized protein	1.61	0.01	0.06
gi|241928760	BGLU42 - beta glucosidase 42	1.61	0.01	0.07
gi|241936911	CHIA - chitinase A	1.61	0.01	0.04
gi|241938592	AT1G62770 - plant invertase/pectin methylesterase inhibitor domain-containing protein	1.61	0.08	0.16
gi|241937628	AT5G05960 - bifunctional inhibitor/lipid-transfer protein/seed storage 2S albumin-like protein	1.60	0.01	0.06
gi|219906464	AT1G71695 - peroxidase 12; Removal of H(2)O(2), oxidation of toxic reductants, biosynthesis and degradation of lignin, suberization, auxin catabolism, response to environmental stresses such as wounding, pathogen attack and oxidative stress. These functions might be dependent on each isozyme/isoform in each plant tissue	1.60	0.00	0.03
gi|241926897	AT4G39830 - putative L-ascorbate oxidase	1.60	0.17	0.24
gi|241921452	ERD9 - glutathione S-transferase; Involved in light signaling, mainly phyA-mediated photomorphogenesis and in the integration of various phytohormone signals to modulate various aspects of plant development by affecting glutathione pools. In vitro, possesses glutathione S- transferase activity toward 1-chloro-2,4-dinitrobenzene (CDNB) and benzyl isothiocyanate (BITC)	1.59	0.01	0.05
gi|241934461	AT2G05790 - O-Glycosyl hydrolases family 17 protein	1.59	0.04	0.11
gi|241945727	AT2G38870 - serine protease inhibitor, potato inhibitor I-type protein	1.59	0.01	0.06

gi|241932527	AOX1A - alternative oxidase 1A; Catalyzes the cyanide-resistant oxidation of ubiquinol and the reduction of molecular oxygen to water, but does not translocate protons and consequently is not linked to oxidative phosphorylation. Increases respiration when the cytochrome respiratory pathway is restricted, or in response to low temperatures	1.59	0.02	0.08
gi|241927225	CSLB01 - cellulose synthase-like B1; Thought to be a Golgi-localized beta-glycan synthase that polymerize the backbones of noncellulosic polysaccharides (hemicelluloses) of plant cell wall	1.58	0.02	0.08
gi|241944359	AATP1 - AAA-ATPase 1	1.58	0.13	0.21
gi|241927971	CYP72A15 - cytochrome P450, family 72, subfamily A, polypeptide 15	1.58	0.02	0.08
gi|241922195	AT3G25290 - putative auxin-responsive protein	1.58	0.01	0.07
gi|241933071	AT2G44530 - ribose-phosphate pyrophosphokinase 5	1.57	0.02	0.09
gi|241931083	GLP5 - germin-like protein 5; May play a role in plant defense. Probably has no oxalate oxidase activity even if the active site is conserved	1.57	0.09	0.17
gi|241940008	AT1G71695 - peroxidase 12; Removal of H(2)O(2), oxidation of toxic reductants, biosynthesis and degradation of lignin, suberization, auxin catabolism, response to environmental stresses such as wounding, pathogen attack and oxidative stress. These functions might be dependent on each isozyme/isoform in each plant tissue	1.57	0.01	0.05
gi|241946335	PNC1 - peroxisomal adenine nucleotide carrier 1; Peroxisomal adenine nucleotide transporter catalyzing the counterexchange of ATP with AMP. ATP is needed by reactions that generate acyl-CoA for peroxisomal fatty acid beta-oxidation during postgerminative growth. Required for the beta-oxidation reactions involved in auxin biosynthesis and for the conversion of seed-	1.57	0.08	0.17

	reserved triacylglycerols into sucrose that is necessary for growth before the onset of photosynthesis			
gi|241918237	fah1 - ferulic acid 5-hydroxylase 1	1.57	0.01	0.06
gi|241919403	AT2G45600 - alpha/beta-hydrolase domain-containing protein; Carboxylesterase acting on esters with varying acyl chain length (By similarity)	1.57	0.02	0.08
gi|241921616	AT1G22410 - class-II DAHP synthetase-like protein	1.57	0.02	0.09
gi|241915743	AT3G28580 - AAA-type ATPase family protein	1.56	0.04	0.11
gi|241945452	AT1G80170 - putative polygalacturonase	1.56	0.00	0.03
gi|241932522	AT2G38250 - DNA-binding protein; Probable transcription factor that may play a role in the induction of CAM4 in response to pathogen and salt	1.56	0.18	0.24
gi|257659117	PPDK - pyruvate, phosphate dikinase 1; Formation of phosphoenolpyruvate. May be involved in regulating the flux of carbon into starch and fatty acids of seeds and in the remobilization of nitrogen reserves in senescing leaves	1.55	0.02	0.08
gi|241915454	AT5G11330 - FAD/NAD(P)-binding oxidoreductase family protein	1.55	0.21	0.26
gi|241929275	GLP5 - germin-like protein 5; May play a role in plant defense. Probably has no oxalate oxidase activity even if the active site is conserved	1.55	0.01	0.07
gi|241921615	AT1G78280 - transferase	1.55	0.10	0.19
gi|241933868	UGT85A1 - cytokinin-O-glucosyltransferase 2; Involved in the O-glucosylation of trans-zeatin and dihydrozeatin. Also active in vitro on cis-zeatin. Not active on N-glucosylated substrates	1.55	0.05	0.14
gi|241920333	PRA7 - PRA1 family protein F2; May be involved in both secretory and endocytic intracellular trafficking in the endosomal/prevacuolar compartments (By similarity)	1.54	0.05	0.13
gi|241946533	LSH5 - uncharacterized protein	1.54	0.06	0.14

gi|241931342	GG1 - Ggamma-subunit 1; Guanine nucleotide-binding proteins (G proteins) are involved as a modulator or transducer in various transmembrane signaling systems. The beta and gamma chains are required for the GTPase activity, for replacement of GDP by GTP, and for G protein- effector interaction. Involved in the abscisic acid (ABA) and ethylene signaling pathways. Regulates acropetal transport of auxin (IAA) in roots and hypocotyls, and thus modulates root architecture (e.g. lateral root formation). The heterotrimeric G-protein controls defense responses to necrotrophic and vascular fungi prob [...]	1.54	0.02	0.08
gi|241945166	AT3G28500 - 60S acidic ribosomal protein P2-3; Plays an important role in the elongation step of protein synthesis (By similarity)	1.53	0.02	0.08
gi|241937676	ARA12 - subtilisin-like protease; Serine protease. Has a substrate preference for the hydrophobic residues Phe and Ala and the basic residue Asp in the P1 position, and for Asp, Leu or Ala in the P1' position	1.53	0.01	0.05
gi|241922385	AT5G60710 - C3H4 type zinc finger protein	1.53	0.00	0.04
gi|241926310	BCS1 - cytochrome BC1 synthesis	1.53	0.05	0.13
gi|241920185	NUP1 - uncharacterized protein	1.53	0.03	0.09
gi|241936479	AT2G03200 - aspartyl protease-like protein	1.53	0.04	0.12
gi|241933017	APG5 - AUTOPHAGY 5; Required for autophagy. Conjugation to ATG12 is essential for plant nutrient recycling	1.52	0.03	0.10
gi|241926527	AT4G27250 - Rossmann-fold NAD(P)-binding domain-containing protein	1.52	0.00	0.03
gi|241919179	APY2 - apyrase 2; Catalyzes the hydrolysis of phosphoanhydride bonds of nucleoside tri- and di-phosphates. Substrate preference is ATP > ADP. Functions with APY1 to reduce extracellular ATP level which is essential for pollen germination and normal	1.52	0.00	0.04

	plant development. Plays a role in the regulation of stomatal function by modulating extracellular ATP levels in guard cells			
gi|241926402	AT1G61260 - uncharacterized protein	1.52	0.03	0.10
gi|241938975	AT3G23600 - dienelactone hydrolase family protein	1.52	0.08	0.16
gi|241925153	AT1G31690 - putative copper amine oxidase	1.51	0.05	0.13
gi|241921860	PYL6 - PYR1-like 6; Receptor for abscisic acid (ABA) required for ABA- mediated responses such as stomatal closure and germination inhibition. Inhibits the activity of group-A protein phosphatases type 2C (PP2Cs) when activated by ABA (By similarity)	1.51	0.04	0.12
gi|241931478	4CL2 - 4-coumarate:CoA ligase 2; Produces CoA thioesters of a variety of hydroxy- and methoxy-substituted cinnamic acids, which are used to synthesize several phenylpropanoid-derived compounds, including anthocyanins, flavonoids, isoflavonoids, coumarins, lignin, suberin and wall-bound phenolics	1.51	0.03	0.10
gi|241941033	PAP27 - purple acid phosphatase 27	1.51	0.02	0.08
gi|241945937	RCI3 - peroxidase 3; Removal of H(2)O(2), oxidation of toxic reductants, biosynthesis and degradation of lignin, suberization, auxin catabolism, response to environmental stresses such as wounding, pathogen attack and oxidative stress. These functions might be dependent on each isozyme/isoform in each plant tissue	1.51	0.02	0.07
gi|241928452	AT3G15290 - 3-hydroxybutyryl-CoA dehydrogenase	1.50	0.05	0.13
gi|241931072	AT1G24620 - putative calcium-binding protein CML25; Potential calcium sensor (By similarity)	1.50	0.07	0.15
gi|241939966	AT4G27450 - aluminum induced protein with YGL and LRDR motifs	0.67	0.00	0.02
gi|259045656	AT3G15670 - late embryogenesis abundant domain-containing protein	0.66	0.01	0.05

gi|241938206	MLP423 - MLP-like protein 423	0.66	0.01	0.05
gi|241919268	PDC2 - pyruvate decarboxylase-2	0.66	0.00	0.02
gi|241935235	ADH1 - alcohol dehydrogenase 1	0.66	0.00	0.03
gi|241921265	ERD9 - glutathione S-transferase; Involved in light signaling, mainly phyA-mediated photomorphogenesis and in the integration of various phytohormone signals to modulate various aspects of plant development by affecting glutathione pools. In vitro, possesses glutathione S- transferase activity toward 1-chloro-2,4-dinitrobenzene (CDNB) and benzyl isothiocyanate (BITC)	0.66	0.03	0.09
gi|241921372	AT4G12510 - bifunctional inhibitor/lipid-transfer protein/seed storage 2S albumin-like protein	0.66	0.01	0.05
gi|241926133	AT1G76010 - Alba DNA/RNA-binding protein	0.66	0.00	0.02
gi|241921476	GSTU18 - glutathione S-transferase TAU 18; May be involved in the conjugation of reduced glutathione to a wide number of exogenous and endogenous hydrophobic electrophiles and have a detoxification role against certain herbicides (By similarity)	0.64	0.00	0.04
gi|241928307	PRF5 - profilin 5; Binds to actin and affects the structure of the cytoskeleton. At high concentrations, profilin prevents the polymerization of actin, whereas it enhances it at low concentrations. By binding to PIP2, it inhibits the formation of IP3 and DG (By similarity)	0.63	0.02	0.08
gi|241922076	XF1 - squalene monooxygenase	0.63	0.00	0.04
gi|666089	scpl26 - serine carboxypeptidase-like 26; Probable carboxypeptidase (By similarity)	0.63	0.00	0.03
gi|241918994	AT2G44310 - calcium-binding EF-hand-containing protein	0.63	0.00	0.02
gi|241940433	TCH4 - Touch 4; Catalyzes xyloglucan endohydrolysis (XEH) and/or endotransglycosylation (XET). Cleaves and religates xyloglucan polymers, an essential constituent of the primary cell wall, and thereby	0.63	0.01	0.07

	participates in cell wall construction of growing tissues. Its induction in case of mechanical stress, suggests that it may contribute in the adaptive changes in morphogenesis by being recruited to alter tissues tensil strength, or flexibility, enabling adaptation to mechanically stressful environments			
gi|241939789		0.62	0.04	0.11
gi|241930262	FLA11 - FASCICLIN-like arabinogalactan-protein 11; May be a cell surface adhesion protein	0.62	0.01	0.04
gi|241920100	AT3G08030 - uncharacterized protein	0.62	0.01	0.07
gi|241927492	AT3G62550 - putative adenine nucleotide alpha hydrolase domain-containing universal stress protein	0.62	0.00	0.03
gi|241933760	CCH - copper chaperone	0.61	0.00	0.02
gi|241921770	AT1G06720 - BMS1 and NUC121 domain-containing protein	0.60	0.01	0.07
gi|241938963	AT3G44590 - 60S acidic ribosomal protein P2-4; Plays an important role in the elongation step of protein synthesis (By similarity)	0.59	0.03	0.10
gi|241922581	AT3G01190 - peroxidase 27; Removal of H(2)O(2), oxidation of toxic reductants, biosynthesis and degradation of lignin, suberization, auxin catabolism, response to environmental stresses such as wounding, pathogen attack and oxidative stress. These functions might be dependent on each isozyme/isoform in each plant tissue	0.59	0.01	0.05
gi|241922582	AT3G01190 - peroxidase 27; Removal of H(2)O(2), oxidation of toxic reductants, biosynthesis and degradation of lignin, suberization, auxin catabolism, response to environmental stresses such as wounding, pathogen attack and oxidative stress. These functions might be dependent on each isozyme/isoform in each plant tissue	0.59	0.01	0.05
gi|241924273	RPS13A - ribosomal protein S13A	0.58	0.00	0.02

gi|241921045	AT3G11930 - adenine nucleotide alpha hydrolases-like protein	0.57	0.01	0.05
gi|241924364	AT3G29970 - B12D protein	0.55	0.02	0.09
gi|241920962	NUC-L2 - nucleolin; Involved in pre-rRNA processing and ribosome assembly (By similarity)	0.52	0.00	0.03
gi|241943389	AT4G32390 - Nucleotide-sugar transporter family protein	0.52	0.02	0.08
gi|241921527	BRU6 - indole-3-acetic acid-amido synthetase GH3.2; Catalyzes the synthesis of indole-3-acetic acid (IAA)-amino acid conjugates, providing a mechanism for the plant to cope with the presence of excess auxin. Strongly reactive with Glu, Gln, Trp, Asp, Ala, Leu, Phe, Gly, Tyr, Met, Ile and Val. Little or no product formation with His, Ser, Thr, Arg, Lys, or Cys. Also active on pyruvic and butyric acid analogs of IAA, PAA and the synthetic auxin naphthaleneacetic acid (NAA). The two chlorinated synthetic auxin herbicides 2,4-D and 3,6-dichloro-o-anisic acid (dicamba) cannot be used as substrates	0.50	0.01	0.05
gi|241933765	AT2G27730 - copper ion binding protein	0.49	0.01	0.04
gi|241935828	AT4G35160 - O-methyltransferase family 2 protein	0.44	0.00	0.03
gi|241944083	AT2G28790 - pathogenesis-related thaumatin-like protein	0.44	0.00	0.02
gi|241934404	AT1G01800 - Rossmann-fold NAD(P)-binding domain-containing protein	0.44	0.00	0.03
gi|241934593	AT4G35160 - O-methyltransferase family 2 protein	0.43	0.01	0.05
gi|241934554	AVP1 - Pyrophosphate-energized vacuolar membrane proton pump 1; Contributes to the transtonoplast (from cytosol to vacuole lumen) H(+)-electrochemical potential difference. It establishes a proton gradient of similar and often greater magnitude than the H(+)-ATPase on the same membrane. In addition, facilitates auxin transport by modulating apoplastic pH and	0.40	0.00	0.02

	regulates auxin-mediated developmental processes. Confers tolerance to NaCl and to drought by increasing ion retention			
gi|241914659	MUR1 - MURUS 1; Catalyzes the conversion of GDP-D-mannose to GDP-4- dehydro-6-deoxy-D-mannose	0.37	0.01	0.04
gi|241939049	PDIL1-2 - PDI-like 1-2; Acts as a protein-folding catalyst that interacts with nascent polypeptides to catalyze the formation, isomerization, and reduction or oxidation of disulfide bonds (By similarity)	0.36	0.01	0.06
gi|241945226	AT5G01320 - pyruvate decarboxylase	0.34	0.00	0.02
gi|241921631	AT4G35160 - O-methyltransferase family 2 protein	0.27	0.00	0.03
gi|241915713	RD21B - esponsive to dehydration 21B	0.27	0.00	0.02
gi|241915714	RD21B - esponsive to dehydration 21B	0.21	0.00	0.02

[1]Average fold change ratio defined as the average intensity of Al treated divided by the average intensity of the control. [2]The variance of the average fold change ration ($\boldsymbol{\sigma}^2$). [3]The standard error ($\boldsymbol{\sigma}/\sqrt{N}$). (For details of the statistical analysis associated with the data presented in this table the reader is referred to the subsection, ”Quantitative protein expression profiles in the sorghum root tip regions” of the Results section of the manuscript.) To convert the GI numbers in this table to the new Accession.Version identifiers use EFetch as described at: https://ncbiinsights.ncbi.nlm.nih.gov/2016/12/06/converting-gi-numbers-to-accession-version/”.

For details of the statistical analysis associated with the data presented in this table, the reader is referred to the subsection "Quantitative protein expression profiles in the sorghum root tip regions" of the Results section of the paper.
