# Supplementary material for: Quantitative iTRAQ Proteomics Revealed Possible Roles for Antioxidant Proteins in Sorghum Aluminum Tolerance
Source: Front Plant Sci. 2017 Jan 9;7:2043. doi: 10.3389/fpls.2016.02043 (PMC5220100; doi:10.3389/fpls.2016.02043)
Supplement: Table S4 — Differentially expressed proteins (fold changes) in 5D SC566. [file Table4.PDF]

Table S4. Differentially Expressed Proteins (Fold Change) in 5D SC566.

Protein ID	Protein Description	Relative Protein Expression[1]	Variance[2]	SE[3]
gi|241933962	HTA11 - histone H2A; Variant histone H2A which may replace conventional H2A in a subset of nucleosomes. Nucleosomes wrap and compact DNA into chromatin, limiting DNA accessibility to the cellular machineries which require DNA as a template. Histones thereby play a central role in transcription regulation, DNA repair, DNA replication and chromosomal stability. DNA accessibility is regulated via a complex set of post-translational modifications of histones, also called histone code, and nucleosome remodeling (By similarity)	5.54	4.21	1.18
gi|241943470	LP1 - lipid transfer protein 1; Plant non-specific lipid-transfer proteins transfer phospholipids as well as galactolipids across membranes. May play a role in wax or cutin deposition in the cell walls of expanding epidermal cells and certain secretory tissues	3.34	0.13	0.20
gi|241929418	LCR83 - putative defensin-like protein 70	3.30	0.83	0.52
gi|241914792	AT2G19750 - 40S ribosomal protein S30	3.10	0.23	0.28
gi|241930937	PEX6 - peroxin 6; Involved in peroxisomal-targeting signal one (PTS1) and peroxisomal-targeting signal two (PTS2) protein import. Required for jasmonate biosynthesis. Necessary for the developmental elimination of obsolete peroxisome matix proteins. May form heteromeric AAA ATPase complexes required for the import of proteins. May be involved in PEX5 recycling	3.09	0.10	0.18
gi|241928801	AT5G48490 - bifunctional inhibitor/lipid-transfer protein/seed storage 2S albumin-like protein	2.94	0.24	0.28

gi|257659117	PPDK - pyruvate, phosphate dikinase 1; Formation of phosphoenolpyruvate. May be involved in regulating the flux of carbon into starch and fatty acids of seeds and in the remobilization of nitrogen reserves in senescing leaves	2.71	0.02	0.08
gi|241937673	AT4G25740 - 40S ribosomal protein S10-1	2.68	0.25	0.29
gi|241947322	HCHIB - chitinase; Defense against chitin containing fungal pathogens. Seems particularly implicated in resistance to jasmonate-inducing pathogens such as A.brassicicola. In vitro antifungal activity against T.reesei, but not against A.solani, F.oxysporum, S.sclerotiorum, G.graminis and P.megasperma	2.53	0.27	0.30
gi|241933947	AT2G31410 - uncharacterized protein	2.43	0.05	0.12
gi|241926425	FLR1 - FLOR1	2.39	0.01	0.05
gi|241918978	SP1L1 - SPIRAL1-like1; Acts redundantly with SPR1 in maintaining the cortical microtubules organization essential for anisotropic cell growth (By similarity)	2.30	0.10	0.19
gi|241915296	AT5G24165 - uncharacterized protein	2.29	0.01	0.06
gi|241935912	AT2G29060 - scarecrow-like protein 34; Probable transcription factor involved in plant development (By similarity)	2.26	0.14	0.22
gi|241930936	cICDH - isocitrate dehydrogenase; May supply 2-oxoglutarate for amino acid biosynthesis and ammonia assimilation via the glutamine synthetase/glutamate synthase (GS/GOGAT) pathway. May be involved in the production of NADPH to promote redox signaling or homeostasis in response to oxidative stress	2.21	0.06	0.14
gi|241931125	AT4G25740 - 40S ribosomal protein S10-1	2.17	0.07	0.15
gi|241945810	TPR5 - tetratricopeptide repeat 5	2.14	0.07	0.16
gi|241919179	APY2 - apyrase 2; Catalyzes the hydrolysis of phosphoanhydride bonds of nucleoside tri- and di-phosphates. Substrate preference is ATP > ADP. Functions with APY1 to reduce extracellular ATP level	2.12	0.13	0.21

	which is essential for pollen germination and normal plant development. Plays a role in the regulation of stomatal function by modulating extracellular ATP levels in guard cells			
gi|241926371	AT2G14095 - uncharacterized protein	2.10	0.20	0.26
gi|241943126	AT5G64130 - cAMP-regulated phosphoprotein 19-related protein	2.06	0.03	0.09
gi|241917737	RS27A - ribosomal protein S27; May be involved in the elimination of damaged mRNA after UV irradiation	2.02	0.05	0.13
gi|241928202	AT2G19730 - 60S ribosomal protein L28-1	2.01	0.12	0.20
gi|241926396	STP1 - sugar transporter 1; Major hexose transporter. Mediates an active uptake of hexoses, by sugar/hydrogen symport. Can transport glucose, 3-O-methylglucose, fructose, xylose, mannose, galactose, fucose, 2- deoxyglucose and arabinose. Confers sensitivity to galactose in seedlings	2.01	0.08	0.17
gi|241924037	AT1G15810 - S15/NS1, RNA-binding protein	2.00	0.11	0.19
gi|241929561	AT3G53770 - late embryogenesis abundant 3 (LEA3) family protein	1.95	0.11	0.19
gi|241922062	GAD - glutamate decarboxylase; Catalyzes the production of GABA. The calmodulin-binding is calcium-dependent and it is proposed that this may, directly or indirectly, form a calcium regulated control of GABA biosynthesis	1.92	0.00	0.04
gi|241933415	RPL10B - ribosomal protein L10 B	1.92	0.09	0.18
gi|241945727	AT2G38870 - serine protease inhibitor, potato inhibitor I-type protein	1.91	0.16	0.23
gi|21326116	AT5G48760 - 60S ribosomal protein L13a-4	1.90	0.05	0.13
gi|241920167	AT1G53645 - hydroxyproline-rich glycoprotein-like protein	1.89	0.02	0.08
gi|241941625	AT5G57410 - Afadin/alpha-actinin-binding protein	1.89	0.08	0.17

gi|241917233	AT5G14050 - U3 small nucleolar RNA-associated protein 18-like protein; Involved in nucleolar processing of pre-18S ribosomal RNA (By similarity)	1.87	0.04	0.12
gi|241918915	AT5G47550 - cysteine proteinase inhibitor 5; Specific inhibitor of cysteine proteinases. Probably involved in the regulation of endogenous processes and in defense against pests and pathogens (By similarity)	1.85	0.09	0.18
gi|241935200	APY1 - apyrase 1; Catalyzes the hydrolysis of phosphoanhydride bonds of nucleoside tri- and di-phosphates. Substrate preference is ATP > ADP. Functions with APY2 to reduce extracellular ATP level which is essential for pollen germination and normal plant development. Plays a role in the regulation of stomatal function by modulating extracellular ATP levels in guard cells	1.85	0.09	0.17
gi|241932353	EXO - EXORDIUM	1.84	0.02	0.08
gi|241941872	HMGA - high mobility group A	1.82	0.03	0.09
gi|241944468	AT5G48760 - 60S ribosomal protein L13a-4	1.82	0.15	0.23
gi|241917078	sks4 - SKU5 similar 4	1.82	0.05	0.13
gi|241917120	AT1G15270 - translation machinery associated protein TMA7	1.81	0.10	0.18
gi|241939131	AT4G39860 - uncharacterized protein	1.81	0.03	0.09
gi|241924205	AT1G11480 - eukaryotic translation initiation factor-like protein	1.76	0.07	0.15
gi|241937018	AT1G27330 - Ribosome associated membrane protein RAMP4	1.76	0.05	0.13
gi|15529117	EP3 - chitinase	1.75	0.08	0.16
gi|241933790	AT2G24600 - ankyrin repeat-containing protein	1.73	0.03	0.10
gi|241934510	AT2G41190 - transmembrane amino acid transporter-like protein	1.72	0.06	0.15
gi|241924929	CHIA - chitinase A	1.71	0.03	0.11
gi|241927004	NADP-ME4 - NADP-malic enzyme 4; The chloroplastic ME isoform decarboxylates malate shuttled from	1.70	0.02	0.07

	neighboring mesophyll cells. The CO(2) released is then refixed by ribulose-bisphosphate carboxylase. This pathway eliminates the photorespiratory loss of CO(2) that occurs in most plants (By similarity)			
gi|241927914	AT4G32460 - uncharacterized protein	1.69	0.02	0.08
gi|241945724	UPI - UNUSUAL SERINE PROTEASE INHIBITOR	1.68	0.00	0.03
gi|241917702	AT4G34290 - SWIB/MDM2 domain-containing protein	1.67	0.05	0.13
gi|241920099	AT3G57490 - 40S ribosomal protein S2-4	1.67	0.11	0.19
gi|241941650	AT3G21215 - RNA recognition motif-containing protein	1.66	0.02	0.08
gi|241930087	AT4G17520 - plasminogen activator inhibitor 1 RNA-binding protein	1.66	0.01	0.06
gi|241938963	AT3G44590 - 60S acidic ribosomal protein P2-4; Plays an important role in the elongation step of protein synthesis (By similarity)	1.65	0.05	0.13
gi|241928446	AT2G21580 - 40S ribosomal protein S25-2	1.65	0.01	0.06
gi|241927362	AT4G15930 - dynein light chain LC8-type	1.65	0.11	0.19
gi|241929209	AT2G37990 - ribosome biogenesis regulatory protein-like protein; Involved in ribosome biogenesis (By similarity)	1.65	0.03	0.09
gi|241916215	AT3G16780 - 60S ribosomal protein L19-2	1.65	0.03	0.09
gi|241943862	AT5G13200 - GEM-like protein 5	1.65	0.08	0.17
gi|241920098	AT3G57490 - 40S ribosomal protein S2-4	1.64	0.08	0.17
gi|241928209	CKL2 - casein kinase 1-like protein 2	1.64	0.04	0.11
gi|241933080	AT2G14880 - SWIB/MDM2 domain-containing protein	1.64	0.03	0.11
gi|241924587	PRX52 - peroxidase 52; Removal of H(2)O(2), oxidation of toxic reductants, biosynthesis and degradation of lignin, suberization, auxin catabolism, response to environmental stresses such as wounding, pathogen attack and oxidative stress. These functions might be dependent on each isozyme/isoform in each plant tissue	1.64	0.07	0.16
gi|253787046	AT3G53740 - 60S ribosomal protein L36-2	1.64	0.07	0.15
gi|241946259	AT3G62810 - complex 1-LYR domain-containing protein	1.63	0.08	0.16

gi|241924585	PRX52 - peroxidase 52; Removal of H(2)O(2), oxidation of toxic reductants, biosynthesis and degradation of lignin, suberization, auxin catabolism, response to environmental stresses such as wounding, pathogen attack and oxidative stress. These functions might be dependent on each isozyme/isoform in each plant tissue	1.62	0.03	0.11
gi|669030678	BGLU42 - beta glucosidase 42	1.62	0.05	0.13
gi|241928713	FTM1 - FLORAL TRANSITION AT THE MERISTEM1; Converts stearoyl-ACP to oleoyl-ACP by introduction of a cis double bond between carbons Delta(9) and Delta(10) of the acyl chain (By similarity)	1.61	0.02	0.07
gi|241921914	MOS11 - modifier of snc1, 11	1.61	0.00	0.03
gi|241930715	AT3G53740 - 60S ribosomal protein L36-2	1.59	0.04	0.12
gi|241928845	BBC1 - breast basic conserved 1	1.59	0.02	0.08
gi|241935975	PRX52 - peroxidase 52; Removal of H(2)O(2), oxidation of toxic reductants, biosynthesis and degradation of lignin, suberization, auxin catabolism, response to environmental stresses such as wounding, pathogen attack and oxidative stress. These functions might be dependent on each isozyme/isoform in each plant tissue	1.59	0.09	0.17
gi|241937908	AT5G11420 - uncharacterized protein	1.59	0.11	0.19
gi|241918870	PUB13 - plant U-box 13; Functions as an E3 ubiquitin ligase (By similarity)	1.58	0.02	0.08
gi|241941680	AT1G79720 - aspartyl protease-like protein	1.58	0.00	0.03
gi|241943043	AT2G28790 - pathogenesis-related thaumatin-like protein	1.57	0.00	0.03
gi|241928841	AT1G23280 - MAK16 protein-like protein	1.57	0.02	0.09
gi|241940544	AT4G10970 - uncharacterized protein	1.57	0.11	0.19
gi|241946819	KCS4 - 3-ketoacyl-CoA synthase 4	1.57	0.04	0.12
gi|241917405	AT3G23390 - 60S ribosomal protein L36a	1.56	0.05	0.13
gi|241931120	AT1G32530 - MND1-interacting protein 1	1.56	0.03	0.10
gi|241941003	AT2G43460 - 60S ribosomal protein L38	1.56	0.13	0.21

gi|241928751	PTAC4 - plastid transcriptionally active 4; Required for plastid vesicle formation and thylakoid membrane biogenesis, but not for functional assembly of thylakoid protein complexes	1.55	0.02	0.08
gi|241945651	AT4G17520 - plasminogen activator inhibitor 1 RNA-binding protein	1.55	0.04	0.11
gi|241930888	AT3G07570 - Cytochrome b561/ferric reductase transmembrane with DOMON related domain	1.55	0.01	0.06
gi|241930917	EXO70F1 - exocyst complex component 7	1.55	0.02	0.08
gi|241940976	AT5G61170 - 40S ribosomal protein S19-3	1.55	0.02	0.08
gi|241934628	AT3G58180 - deoxyhypusine hydroxylase; Catalyzes the hydroxylation of the N(6)-(4-aminobutyl)- L-lysine intermediate to form hypusine, an essential post-translational modification only found in mature eIF-5A factor (By similarity)	1.54	0.11	0.19
gi|241917462	TIM10 - mitochondrial import inner membrane translocase subunit Tim10	1.54	0.10	0.18
gi|241935820	GSTU18 - glutathione S-transferase TAU 18; May be involved in the conjugation of reduced glutathione to a wide number of exogenous and endogenous hydrophobic electrophiles and have a detoxification role against certain herbicides (By similarity)	1.54	0.01	0.05
gi|241926357	AT2G43460 - 60S ribosomal protein L38	1.54	0.01	0.07
gi|241922908	AT3G57490 - 40S ribosomal protein S2-4	1.54	0.04	0.12
gi|241926125	ACP2 - acyl carrier protein 2; Carrier of the growing fatty acid chain in fatty acid biosynthesis (By similarity)	1.54	0.06	0.14
gi|241927239	LPR1 - Low Phosphate Root1	1.53	0.01	0.07
gi|241917173	AT2G44065 - ribosomal protein L2-like protein	1.53	0.01	0.07
gi|241929513	CHR8 - chromatin remodeling 8	1.53	0.01	0.05
gi|241919147	AUD1 - UDP-glucuronic acid decarboxylase; Catalyzes the NAD-dependent decarboxylation of UDP- glucuronic acid to UDP-xylose. Necessary for the biosynthesis of	1.53	0.05	0.13

	the core tetrasaccharide in glycosaminoglycan biosynthesis			
gi|241942843	EMB3010 - embryo defective 3010; May play an important role in controlling cell growth and proliferation through the selective translation of particular classes of mRNA (By similarity)	1.52	0.05	0.13
gi|241946063	AT1G67430 - 60S ribosomal protein L17-2	1.52	0.04	0.11
gi|241930880	LOX1 - lipoxygenase 1; 9S-lipoxygenase that can use linoleic acid or linolenic acid as substrates. Plant lipoxygenases may be involved in a number of diverse aspects of plant physiology including growth and development, pest resistance, and senescence or responses to wounding. Catalyzes the hydroperoxidation of lipids containing a cis,cis-1,4-pentadiene structure. Function as regulators of root development by controlling the emergence of lateral roots	1.52	0.01	0.04
gi|241918359	AT1G04480 - 60S ribosomal protein L23	1.52	0.03	0.10
gi|241924945	TRX1 - thioredoxin H1; Thiol-disulfide oxidoreductase involved in the redox regulation of a number of cytosolic enzymes. Activates the cytosolic malate dehydrogenase (MDH) probably by reducing an interchain disulfid bond of the inactive MDH homodimer. Possesses insulin disulfide bonds reducing activity	1.52	0.08	0.16
gi|241932937	ASG5 - ALTERED SEED GERMINATION 5	1.52	0.10	0.18
gi|241940972	AT3G54630 - kinetochore protein NDC80	1.52	0.07	0.16
gi|241926327	ICL - isocitrate lyase; Involved in storage lipid mobilization during the growth of higher plant seedling	1.51	0.11	0.19
gi|241922004	DRP4C - Dynamin related protein 4C; Putative microtubule-associated force-producing protein, able to bind and hydrolyze GTP (By similarity)	1.51	0.02	0.08

gi|241930736	CRR2 - CHLORORESPIRATORY REDUCTION 2; Required for the intergenic processing between chloroplast rsp7 and ndhB transcripts	1.51	0.01	0.07
gi|168805244	FAD2 - fatty acid desaturase 2; ER (microsomal) omega-6 fatty acid desaturase introduces the second double bond in the biosynthesis of 18:3 fatty acids, important constituents of plant membranes. It is thought to use cytochrome b5 as an electron donor and to act on fatty acids esterified to phosphatidylcholine and, possibly, other phospholipids	1.51	0.04	0.12
gi|390176211	NADP-ME4 - NADP-malic enzyme 4; The chloroplastic ME isoform decarboxylates malate shuttled from neighboring mesophyll cells. The CO(2) released is then refixed by ribulose-bisphosphate carboxylase. This pathway eliminates the photorespiratory loss of CO(2) that occurs in most plants (By similarity)	1.50	0.02	0.09
gi|219898273	RPS13A - ribosomal protein S13A	1.50	0.03	0.10
gi|241941998	DIC2 - dicarboxylate carrier 2; PUMPS are mitochondrial transporter proteins that create proton leaks across the inner mitochondrial membrane, thus uncoupling oxidative phosphorylation. This leads to a decrease in the efficiency of oxidative phosphorylation and an increase in heat production. May be involved in protecting plant cells against oxidative stress damage (By similarity). Recombinant PUMP4, reconstituted into liposomes, transports a wide range of dicarboxylic acids including malate, oxaloacetate and succinate as well as phosphate, sulfate and thiosulfate. However, it is unknown if t [...]	1.50	0.02	0.09
gi|241930393	AT4G17520 - plasminogen activator inhibitor 1 RNA-binding protein	1.50	0.02	0.09
gi|241926717	AT3G57320 - uncharacterized protein	1.50	0.11	0.20
gi|241930039	AT5G20970 - heat shock family protein	0.67	0.03	0.09

gi|241937376	EXPA11 - expansin 11; Causes loosening and extension of plant cell walls by disrupting non-covalent bonding between cellulose microfibrils and matrix glucans. No enzymatic activity has been found (By similarity)	0.67	0.00	0.03
gi|241932049	AT5G46930 - plant invertase/pectin methylesterase inhibitor domain-containing protein	0.67	0.01	0.04
gi|241932789	AT4G33400 - Vacuolar import/degradation, Vid27-related protein	0.67	0.01	0.05
gi|241916411	AOP1 - oxidoreductase AOP1	0.67	0.00	0.01
gi|241927529	AT5G19290 - esterase/lipase/thioesterase family protein	0.66	0.01	0.04
gi|8979720	CBSX3 - CBS domain-containing protein	0.66	0.00	0.03
gi|241934339	PIP1;4 - putative aquaporin PIP1-4; Water channel required to facilitate the transport of water across cell membrane. Essential for the water permeability of the plasma membrane and for the morphology of the root system. Its function is impaired by Hg(2+). Inhibited by cytosolic acidosis which occurs during anoxia in roots	0.66	0.01	0.05
gi|257632199	AT3G14360 - lipase class 3 family protein	0.66	0.00	0.03
gi|241929989	THI1 - thiazole biosynthetic enzyme; Involved in biosynthesis of the thiamine precursor thiazole. Catalyzes the conversion of NAD and glycine to adenosine diphosphate 5-(2-hydroxyethyl)-4-methylthiazole-2-carboxylic acid (ADT), an adenylated thiazole intermediate. The reaction includes an iron-dependent sulfide transfer from a conserved cysteine residue of the protein to a thiazole intermediate. The enzyme can only undergo a single turnover, which suggests it is a suicide enzyme. May have additional roles in adaptation to various stress conditions and in DNA damage tolerance	0.66	0.01	0.05
gi|241939266	MLS - malate synthase	0.66	0.00	0.03

gi|241940359	AT1G56130 - putative LRR receptor-like serine/threonine-protein kinase	0.66	0.00	0.03
gi|241944550	AT2G35840 - putative sucrose-phosphatase 2; Catalyzes the final step of sucrose synthesis (By similarity)	0.65	0.00	0.03
gi|241915410	BGAL1 - beta galactosidase 1	0.65	0.01	0.06
gi|241918994	AT2G44310 - calcium-binding EF-hand-containing protein	0.65	0.00	0.04
gi|241946839	DSEL - lipase class 3 family protein; Acylhydrolase that catalyzes the hydrolysis of 1,3- diacylglycerol (1,3-DAG) and 1-monoacylglycerol (1-MAG) at the sn- 1 position. High activity toward 1,3-DAG and 1-MAG, but low activity toward 1,2-diacylglycerol (1,2-DAG) and 1-lysophosphatidylcholine (1-LPC), and no activity toward phosphatidylcholine (PC), monogalactosyldiacylglycerol (MGDG), digalactosyldiacylglycerol (DGDG), triacylglycerol (TAG) and 2- monoacylglycerol (2-MAG). May be involved in the negative regulation of seedling establishment by inhibiting the breakdown, beta-oxidation and mobi [...]	0.65	0.01	0.05
gi|241932651	UCC1 - uclacyanin 1	0.65	0.00	0.04
gi|241928923	AT3G27890.1 - NADPH:quinone oxidoreductase; The enzyme apparently serves as a quinone reductase in connection with conjugation reactions of hydroquinones involved in detoxification pathways	0.65	0.00	0.04
gi|241933760	CCH - copper chaperone	0.65	0.00	0.03
gi|241925698	AMY1 - alpha-amylase; Possesses alpha-amylase activity in vitro, but seems not required for breakdown of transitory starch in leaves	0.64	0.01	0.05
gi|241931547	AT5G12960 - uncharacterized protein	0.64	0.00	0.04
gi|241939472	TIP2;3 - aquaporin TIP2-3; Transports methylammonium or ammonium in yeast cells, preferentially at high medium pH. May participate in	0.64	0.01	0.05

	vacuolar compartmentation and detoxification of ammonium			
gi|241919268	PDC2 - pyruvate decarboxylase-2	0.63	0.00	0.03
gi|241916095	AT3G02645 - uncharacterized protein	0.63	0.01	0.05
gi|241926130	UCC1 - uclacyanin 1	0.62	0.02	0.08
gi|241934435	AREB3 - ABA-responsive element binding protein 3; Binds to the embryo specification element and the ABA-responsive element (ABRE) of the Dc3 gene promoter. Could participate in abscisic acid-regulated gene expression during seed development	0.62	0.00	0.04
gi|241925338	SAG12 - senescence-associated gene 12	0.62	0.00	0.02
gi|241935235	ADH1 - alcohol dehydrogenase 1	0.62	0.00	0.03
gi|241923158	SCPL19 - serine carboxypeptidase-like 19; Involved in plants secondary metabolism. Functions as acyltransferase to form the sinapate ester sinapoylcholine also known as sinapine. Able to convert in vitro benzoylglucose into benzoylcholine	0.62	0.00	0.04
gi|241930956		0.62	0.01	0.05
gi|241945004	ANNAT3 - annexin 3	0.61	0.00	0.02
gi|241918897	PLDBETA1 - phospholipase D; Hydrolyzes glycerol-phospholipids at the terminal phosphodiesteric bond. Plays an important role in various cellular processes, including phytohormone action, vesicular trafficking, secretion, cytoskeletal arrangement, meiosis, tumor promotion, pathogenesis, membrane deterioration and senescence. Can use phosphatidylserine or N-acylphosphatidylethanolamine as substrates	0.61	0.02	0.08
gi|241921045	AT3G11930 - adenine nucleotide alpha hydrolases-like protein	0.61	0.01	0.05
gi|241921420	SSL4 - strictosidine synthase-like 4	0.61	0.01	0.04
gi|241932738	PYL11 - PYR1-like 11; Receptor for abscisic acid (ABA) required for ABA- mediated responses such as stomatal closure and germination inhibition. Inhibits the activity of	0.60	0.00	0.04

	group-A protein phosphatases type 2C (PP2Cs) when activated by ABA (By similarity)			
gi|241932639	TIP2;3 - aquaporin TIP2-3; Transports methylammonium or ammonium in yeast cells, preferentially at high medium pH. May participate in vacuolar compartmentation and detoxification of ammonium	0.60	0.01	0.05
gi|241935937	ASN1 - glutamine-dependent asparagine synthase 1; Essential for nitrogen assimilation, distribution and remobilization within the plant via the phloem	0.60	0.00	0.02
gi|241939412	AT3G61260 - remorin-like protein	0.60	0.00	0.03
gi|241926724	AT3G26770 - Rossmann-fold NAD(P)-binding domain-containing protein	0.60	0.01	0.04
gi|241942691	AT1G79620 - leucine-rich repeat protein kinase-like protein	0.60	0.00	0.03
gi|241921265	ERD9 - glutathione S-transferase; Involved in light signaling, mainly phyA-mediated photomorphogenesis and in the integration of various phytohormone signals to modulate various aspects of plant development by affecting glutathione pools. In vitro, possesses glutathione S- transferase activity toward 1-chloro-2,4-dinitrobenzene (CDNB) and benzyl isothiocyanate (BITC)	0.60	0.00	0.03
gi|241915713	RD21B - esponsive to dehydration 21B	0.60	0.01	0.06
gi|241918572	AT1G64710 - putative alcohol dehydrogenase	0.60	0.00	0.04
gi|241931153	ZIP2 - zinc transporter 2; Mediates zinc uptake. May also transport copper and cadmium ions	0.59	0.00	0.02
gi|241943398	ASD1 - alpha-L-arabinofuranosidase 1; May be involved in the coordinated dissolution of the cell wall matrix during abscission and in the secondary cell wall formation in xylem vessels. Prefers arabinoxylan, but may also use pectic arabinans as substrates	0.59	0.00	0.02

gi|241918315	CYP86B1 - cytochrome P450 86B1; Involved in very long chain fatty acids (VLCFA) omega- hydroxylation. Required for the synthesis of saturated VLCFA alpha, omega-bifunctional suberin monomers	0.59	0.01	0.04
gi|241924584	PRX52 - peroxidase 52; Removal of H(2)O(2), oxidation of toxic reductants, biosynthesis and degradation of lignin, suberization, auxin catabolism, response to environmental stresses such as wounding, pathogen attack and oxidative stress. These functions might be dependent on each isozyme/isoform in each plant tissue	0.58	0.00	0.02
gi|241921012	PHT1;7 - phosphate transporter 1;7; High-affinity transporter for external inorganic phosphate (By similarity)	0.57	0.00	0.03
gi|241946128	WIT1 - WPP domain-interacting protein 1; Together with WIT2, required for the nuclear envelope docking of RANGAP proteins in root tips	0.57	0.01	0.04
gi|241937145	AT1G03230 - aspartyl protease-like protein	0.57	0.01	0.06
gi|241932314	PIP2A - aquaporin PIP2-1; Water channel required to facilitate the transport of water across cell membrane. Probably involved in root water uptake. Its function is impaired by Hg(2+)	0.56	0.00	0.04
gi|241925187	DIR6 - dirigent protein 6	0.56	0.00	0.04
gi|241925144	AT1G73040 - jacalin-like lectin domain-containing protein	0.56	0.01	0.05
gi|241919092	DSEL - lipase class 3 family protein; Acylhydrolase that catalyzes the hydrolysis of 1,3- diacylglycerol (1,3-DAG) and 1-monoacylglycerol (1-MAG) at the sn- 1 position. High activity toward 1,3-DAG and 1-MAG, but low activity toward 1,2-diacylglycerol (1,2-DAG) and 1-lysophosphatidylcholine (1-LPC), and no activity toward phosphatidylcholine (PC), monogalactosyldiacylglycerol (MGDG), digalactosyldiacylglycerol (DGDG), triacylglycerol (TAG) and 2- monoacylglycerol (2-MAG).	0.56	0.00	0.02

	May be involved in the negative regulation of seedling establishment by inhibiting the breakdown, beta-oxidation and mobi [...]			
gi|241941843	AT1G52820 - putative 2-oxoglutarate-dependent dioxygenase	0.56	0.01	0.05
gi|241939917	MTK - S-methyl-5-thioribose kinase; Catalyzes the phosphorylation of methylthioribose into methylthioribose-1-phosphate in the methionine cycle. Contributes to the maintenance of AdoMet homeostasis and is required to sustain high rates of ethylene synthesis	0.55	0.00	0.03
gi|241925946	BGLU17 - beta glucosidase 17	0.55	0.00	0.03
gi|241946494	AKR4C10 - Aldo-keto reductase family 4 member C10; Oxidoreductase that may act on a broad range of substrates such as ketosteroids, aldehydes, ketones and sugars (By similarity)	0.54	0.00	0.03
gi|241927712	ABCB12 - P-glycoprotein 12	0.54	0.01	0.06
gi|241930797	RWP1 - REDUCED LEVELS OF WALL-BOUND PHENOLICS 1; Involved in the synthesis of aromatics of the suberin polymer. Specifically affects the accumulation of the ferulate constituent of suberin in roots and seeds, but has no effect on the content of p-coumarate or sinapate	0.53	0.00	0.01
gi|241923857	AIR12 - auxin-responsive-like protein	0.53	0.01	0.04
gi|241916653	DELTA-TIP - aquaporin TIP2-1; Aquaporin required to facilitate the transport of water from the vacuolar compartment to the cytoplasm. Does not promote glycerol permability. Its function is impaired by Hg(2+). Transports urea in yeast cells and Xenopus laevis oocytes in a pH- independent manner. Transports methylammonium or ammonium in yeast cells and Xenopus laevis oocytes, preferentially at high medium	0.52	0.00	0.03

	pH. May participate in vacuolar compartmentation and detoxification of ammonium			
gi|241944106	AT1G13750 - putative inactive purple acid phosphatase 1	0.51	0.00	0.04
gi|241921626	COPT5 - copper transporter 5; Involved in the transport of copper	0.51	0.00	0.04
gi|241919699	PHT1;7 - phosphate transporter 1;7; High-affinity transporter for external inorganic phosphate (By similarity)	0.51	0.01	0.07
gi|241934239	FDH - Formate dehydrogenase	0.50	0.01	0.05
gi|241919321	DSI-1VOC - dessication-induced 1VOC-like protein	0.50	0.01	0.06
gi|241940766	ALDH11A3 - aldehyde dehydrogenase 11A3; Important as a means of generating NADPH for biosynthetic reactions	0.49	0.00	0.04
gi|241934432	AMY1 - alpha-amylase; Possesses alpha-amylase activity in vitro, but seems not required for breakdown of transitory starch in leaves	0.48	0.01	0.05
gi|241944467	AT1G19715 - mannose-binding lectin-like protein	0.48	0.00	0.03
gi|241938626	AT4G10540 - Subtilase family protein	0.48	0.01	0.04
gi|144583703	AT4G35160 - O-methyltransferase family 2 protein	0.48	0.00	0.04
gi|241920962	NUC-L2 - nucleolin; Involved in pre-rRNA processing and ribosome assembly (By similarity)	0.48	0.00	0.02
gi|241922056	AT1G59960 - putative Aldo/keto reductase	0.47	0.00	0.02
gi|241928617	SOUL-1 - SOUL heme-binding-like protein	0.47	0.00	0.04
gi|241935828	AT4G35160 - O-methyltransferase family 2 protein	0.45	0.01	0.05
gi|241922951	AT3G07720 - galactose oxidase/kelch repeat-containing protein	0.42	0.00	0.03
gi|241945226	AT5G01320 - pyruvate decarboxylase	0.41	0.00	0.04
gi|241936910	CHIA - chitinase A	0.40	0.00	0.04
gi|241922750	AT5G36160 - tyrosine aminotransferase; Transaminase involved in tyrosine breakdown. Converts tyrosine to p-hydroxyphenylpyruvate. Can catalyze the reverse reaction, using L-glutamate in vitro. Can convert	0.38	0.00	0.01

	phenylalanine to phenylpyruvate and catalyze the reverse reaction in vitro			
gi|241921631	AT4G35160 - O-methyltransferase family 2 protein	0.36	0.01	0.05
gi|241934554	AVP1 - Pyrophosphate-energized vacuolar membrane proton pump 1; Contributes to the transtonoplast (from cytosol to vacuole lumen) H(+)-electrochemical potential difference. It establishes a proton gradient of similar and often greater magnitude than the H(+)-ATPase on the same membrane. In addition, facilitates auxin transport by modulating apoplastic pH and regulates auxin-mediated developmental processes. Confers tolerance to NaCl and to drought by increasing ion retention	0.35	0.00	0.02
gi|241915714	RD21B - esponsive to dehydration 21B	0.35	0.00	0.04
gi|241922581	AT3G01190 - peroxidase 27; Removal of H(2)O(2), oxidation of toxic reductants, biosynthesis and degradation of lignin, suberization, auxin catabolism, response to environmental stresses such as wounding, pathogen attack and oxidative stress. These functions might be dependent on each isozyme/isoform in each plant tissue	0.34	0.00	0.04
gi|241944083	AT2G28790 - pathogenesis-related thaumatin-like protein	0.32	0.00	0.04
gi|241942899	AT4G10500 - oxidoreductase, 2OG-Fe(II) oxygenase family protein	0.23	0.00	0.03
gi|241942902	AT4G10500 - oxidoreductase, 2OG-Fe(II) oxygenase family protein	0.17	0.00	0.01

[1]Average fold change ratio defined as the average intensity of Al treated divided by the average intensity of the control. [2]The variance of the average fold change ration ($\boldsymbol{\sigma}^2$). [3]The standard error ($\boldsymbol{\sigma}/\sqrt{N}$). (For details of the statistical analysis associated with the data presented in this table the reader is referred to the subsection, ”Quantitative protein expression profiles in the sorghum root tip regions” of the Results section of the manuscript.) To convert the GI numbers in this table to the new Accession.Version identifiers use EFetch as described at: https://ncbiinsights.ncbi.nlm.nih.gov/2016/12/06/converting-gi-numbers-to-accession-version/”.

For details of the statistical analysis associated with the data presented in this table, the reader is referred to the subsection "Quantitative protein expression profiles in the sorghum root tip regions" of the Results section of the paper.
