# Supplementary material for: Quantitative iTRAQ Proteomics Revealed Possible Roles for Antioxidant Proteins in Sorghum Aluminum Tolerance
Source: Front Plant Sci. 2017 Jan 9;7:2043. doi: 10.3389/fpls.2016.02043 (PMC5220100; doi:10.3389/fpls.2016.02043)
Supplement: Table S5 — Relative protein expression (fold changes) of antioxidant enzymes in BR007 and SC566 under Al treatment. [file Table5.PDF]

Table S5. Relative Protein Expression (Fold Change) of Antioxidant Enzymes in BR007 and SC566.

Protein ID	Gene	SC566 3D	SC566 5D	BR007 3D	BR007 5D
gi|241935975	PRX52 - peroxidase 52	1.65±0.11	1.59±0.17	2.49±0.15	3.20±0.14
gi|241935976	PRX52 - peroxidase 52	2.33±0.08	N	2.26±0.16	2.81±0.15
gi|241916757	PRX52 - peroxidase 52	N	N	0.60±0.02	0.58±0.03
gi|241916753	PRX52 - peroxidase 52	N	N	0.50±0.02	0.53±0.04
gi|241924585	PRX52 - peroxidase 52	N	1.62±0.11	N	N
gi|241942396	PRX52 - peroxidase 52	N	N	N	1.89±0.16
gi|241931679	PRX52 - peroxidase 52	1.71±0.09	N	N	N
gi|241931677	PRX52 - peroxidase 52	N	N	0.60±0.02	0.53±0.01
gi|241940007	PRX12 - peroxidase 12	N	N	N	0.54±0.04
gi|241940006	PRX12 - peroxidase 12	N	N	N	1.81±0.05
gi|241940008	PRX12 - peroxidase 12	1.57±0.05	N	1.54±0.07	1.55±0.12
gi|219906464	PRX12 - peroxidase 12	1.60±0.03	N	N	N
gi|241945937	RCI3 - peroxidase 3	1.51±0.07	N	N	1.84±0.13
gi|241945305	RCI3-peroxidase 3	N	N	0.64±0.02	0.47±0.03
gi|241926632	RCI3 - peroxidase 3	N	N	2.40±0.08	2.48±0.14
gi|241919822	RCI3-peroxidase 3	N	N	0.59±0.03	N
gi|241920768	PRX 56- peroxidase 56	N	N	N	0.65±0.02
gi|241927543	PRX 56- peroxidase 56	N	N	N	0.66±0.05
gi|241922581	PRX27 - peroxidase 27	0.59±0.05	0.34±0.04	0.54±0.05	N
gi|241927623	PRX - peroxidase	N	N	N	0.63±0.05
gi|241927471	PRX - peroxidase	N	N	0.65±0.05	0.51±0.03
gi|241929684	PRX - peroxidase	N	N	0.44±0.02	0.42±0.06
gi|241925900	PRX - peroxidase	N	N	1.74±0.04	2.62±0.19
gi|241926637	PA2 - peroxidase 2	1.81±0.07	N	2.09±0.31	2.66±0.62
gi|241926633	PRX5 - peroxidase 5	N	N	3.11±0.35	N
gi|241927409	PRX24- peroxidase 24	N	N	0.63±0.02	0.50±0.04
gi|241941045	PRX51- peroxidase 51	N	N	N	0.53±0.02
gi|241945829	PRX72 - peroxidase 72	N	N	0.60±0.05	0.43±0.03
gi|241946418	PRX54 - peroxidase 54	N	N	0.53±0.02	0.33±0.02

gi|241937302	APX3 - L-ascorbate peroxidase	N	N	1.82±0.04	N
gi|241933008	CAT1(catalase)	2.73±0.18	N	2.56±0.16	4.44±0.44

Note: N indicates that there was no significant change compared to control. Numbers are ratios of Al^{3+} average/control average.
