# Supplementary figures and images for: Quantitative iTRAQ Proteomics Revealed Possible Roles for Antioxidant Proteins in Sorghum Aluminum Tolerance
Source: Front Plant Sci. 2017 Jan 9;7:2043. doi: 10.3389/fpls.2016.02043 (PMC5220100; doi:10.3389/fpls.2016.02043)

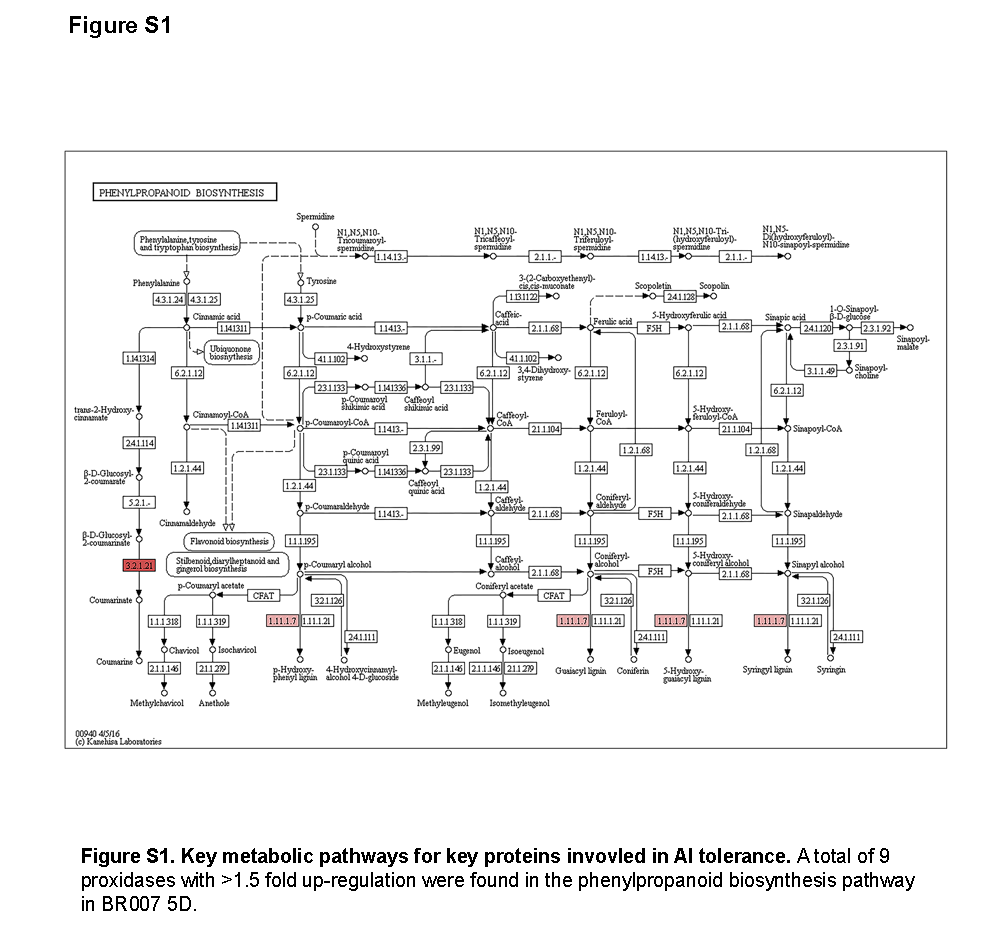

Supplement: Figure S1 — Key metabolic pathways for key proteins involved in Al tolerance. A total of nine peroxidases with >1.5-fold upregulation were found in the phenylpropanoid biosynthesis pathway in BR007 5D. [file Image1.tif]

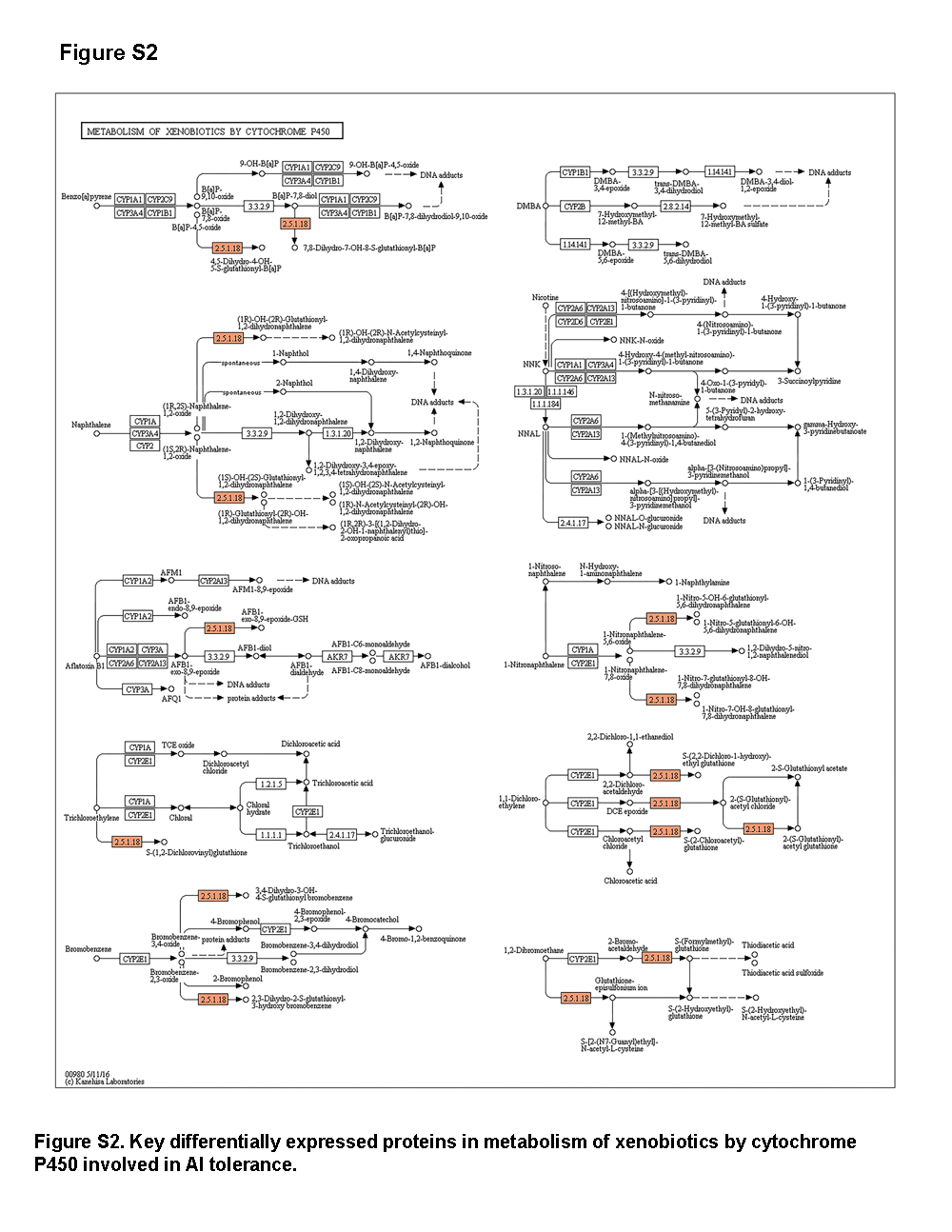

Supplement: Figure S2 — Key differentially expressed proteins in metabolism of xenobiotics by cytochrome P450 involved in Al tolerance. [file Image2.tif]
